# Supplementary material for: Oxygen evolution reaction on IrO2(110) is governed by Walden-type mechanisms
Source: Nat Commun. 2025 Jul 3;16:6137. doi: 10.1038/s41467-025-61367-z (PMC12229486; doi:10.1038/s41467-025-61367-z)
Supplement: Supplementary file 1 — Supplementary Information [file 41467_2025_61367_MOESM1_ESM.pdf]

Supporting Information

Oxygen evolution reaction on IrO_2(110) is governed by Walden-type mechanisms

Muhammad Usama[1,2], Samad Razzaq[1], Christof Hättig[2,3], Stephan N. Steinmann[4], Kai S. Exner[1,2,5,*]

[1] *University Duisburg-Essen, Faculty of Chemistry, Theoretical Inorganic Chemistry, Universitätsstraße 5, 45141 Essen, Germany*

[2] *Cluster of Excellence RESOLV, Bochum, Germany*

[3] *Lehrstuhl für Theoretische Chemie, Ruhr-Universität Bochum, D-44780 Bochum*

[4] *CNRS, ENS de Lyon, LCH, UMR 5182, 69342, Lyon cedex 07, France*

[5] *Center for Nanointegration (CENIDE) Duisburg-Essen, Duisburg, Germany*

* Corresponding author: kai.exner@uni-due.de ORCID: 0000-0003-2934-6075

Keywords

oxygen evolution reaction; IrO_2; descriptor approach; reaction mechanism; Walden inversion

1 Computational Details

To comprehend the elementary steps of the oxygen evolution reaction (OER) over IrO_2(110), we apply electronic structure calculations in the density functional theory (DFT) approximation as implemented in the Vienna Ab Initio Simulation Package (VASP)[1–3]. To visualize and construct the unit cell and slab models, the VESTA software is used[4].

Initially, we optimize the bulk structure of rutile IrO_2 and obtain unit cell dimensions of $a = b =$ 4.50 Å and $c = 3.15$ Å. These values closely resemble experimental reports from the literature ($a = b = 4.51$ Å, $c = 3.16$ Å).[5] Subsequently we construct a (2x1) surface slab model along the (110) direction with the aid of VESTA. Benchmark calculations were performed to optimize the plane-wave basis set cutoff energy (ENCUT), the k-point mesh, and the lattice constants of the crystal structure. In our study, we use ENCUT = 440 eV and a 7 x 7 x 1 Γ-centered k-point mesh to sample the Brillouin zone for the numerical integration in the reciprocal space. A 5-layered asymmetric slab model is chosen for the determination of free energies for adsorbate structures on-top of the (2x1) IrO_2(110) surface. While the atoms in the lower two layers are fixed, all atoms of the upper three layers including adsorbates are allowed to freely relax. To minimize interactions between periodic images, a vacuum gap of at least 12 Å is incorporated along the surface normal direction.

For the description of the electronic structure, the Perdew-Burke-Ernzerhof (PBE)[6] and revised PBE (RPBE)[7] exchange correlation functionals are employed, along with the DFT-D3 method of Grimme et al.[8] to correct the obtained energetics in terms of dispersion effects. Additionally, a dipole correction in the direction perpendicular to the surface is included in our calculations due to the choice of an asymmetric slab model. We make use of projector augmented wave (PAW)

pseudopotentials[9] with the cutoff energy specified in the above for the plane-wave-basis set. The Methfessel-Paxton smearing method is applied for the atomic structure optimization with a smearing width of 0.20 eV. The convergence criterion for forces and convergence of the total energy along the self-consistent field (SCF) is set to 0.01 eV/Å and 10^{-6} eV, respectively.
To describe the impact of the aqueous electrolyte on the adsorption free energies, we apply a continuum model in the realm of implicit solvation by referring to the VASPsol package.[10,11] The relative permittivity of water and the Debye length are set to 78.4 and 3.0 Å (corresponding to 1 M bulk electrolyte concentration), respectively.
Electrochemical experiments are carried out under constant potentials whereas the most popular approach to determine adsorption free energies refers to canonical DFT calculations (constant charge). To this end, we apply grand canonical DFT (GC-DFT) calculations at constant potential using VASP version 6.4.1 in combination with VASPsol implicit solvation model. While the version of VASPsol used in our work does not explicitly offer a built-in GC-DFT feature, this functionality is now available in the newer VASPsol++ implementation[12]. In our calculations, constant-potential conditions are achieved by calibrating the electron chemical potential relative to the standard hydrogen electrode (SHE)[11]. In this approach, the electrochemical potential of the electron in the system is kept constant, while the charge of the system can vary. The absolute potential is determined as the difference between the Fermi level and the vacuum level, where this value is related to the calibrated electron chemical potential of −4.66 eV, which corresponds to 0 V vs. SHE.[13] The calculation begins with the definition of a target potential. The number of electrons in the system is iteratively adjusted to ensure that the Fermi energy matches the specified potential. The applied electrode potential is then expressed as:

$$U = -(\mathit{shift} + \mathit{Fermi\ energy} + 4.66\ \text{eV}) \quad (1)$$

where the shift (representing the electrostatic potential) and the Fermi energy are extracted directly from the VASPsol outputs.
Furthermore, in the grand-canonical formalism, the thermodynamic potential of interest is the grand free energy (Ω), as opposed to the standard (Gibbs) free energy. The grand free energy is calculated using the expression:

$$\Omega = E_{\text{tot}} - (N_{\text{q}} - N_0) \times \mathit{Fermi\ energy} \quad (2)$$

where N_{q} is the number of electrons at the target potential, and N_0 is the number of electrons in the neutral system.
It is important to note that the vacuum level is not explicitly included in VASPsol. To account for this, the chemical potential of the electron at 0 V vs. SHE is calibrated based on the experimental potential of zero charge (PZC) for specific transition-metal electrodes. For further information, we relate the reader to the corresponding VASPsol publication[11].
For the assessment of electronic structure alterations and comprehension of charge state variations during the electrocatalytic processes of OER, we apply Bader charge analysis using the Henkelman Group script[14] designed for VASP.

2 Pourbaix Diagram of IrO_2 (110)

2.1 Pourbaix diagrams in electrocatalysis

In the following, we discuss the construction of a Pourbaix diagram for the IrO_2(110) surface under OER conditions. This is achieved by the connection of DFT calculations, analyzed by means of the computational hydrogen electrode (CHE) approach[15], with a thermodynamic-electrochemical evaluation of adsorption free energies for different adsorbate structures.[16–18]

Pourbaix diagrams indicate the energetically most stable surface structure as a function of the applied electrode potential, *U*, and pH. To gain insight into the surface structure of a single-crystalline IrO_2(110) model electrode under OER conditions, we determine the adsorption free energies for a variety of surface configurations containing the *OH, *O, *OOH, and *OO adsorbates on coordinatively unsaturated Ir surface sites, as these species are reconciled with intermediate structures during the OER. Thereafter, we minimize the free energies of the various surface phases as a function of *U* and pH, and the thermodynamically preferred surface configuration with the lowest free energy is displayed in the Pourbaix diagram.

It is a common consensus in the theoretical electrochemistry community that the energetically favored surface phase at the equilibrium potential of an electrocatalytic process is used as the active surface configuration for the modeling of mechanistic pathways[19–27]. While our current study builds upon this notion, we go one step beyond this approximation in that we model the mechanistic pathways over a variety of different surface phases that reveal a similar energetics (cf. Figure 1b-e in the main text). This finding is further discussed in the main text of our article, and in the following we provide a thorough mathematical framework for the determination of ab initio Pourbaix diagrams.

2.2 Mathematical framework

2.2.1 *Adsorption processes and reference states*

DFT-based Pourbaix diagrams are constructed based on a thermodynamic evaluation of reaction equations for a well-defined reference state. On the one hand, we select the stoichiometric IrO_2(110) surface – $2O_{br} + 2*_{cus}$ – as a reference (cf. Figure S1). In this surface phase, the coordinatively unsaturated (cus) Ir_{cus} sites are vacant, while the iridium bridge (br) sites, Ir_{br}, are capped by surface oxygen, O_{br}.

Adsorption processes can take place on both the Ir_{cus} and Ir_{br} sites; e. g., the formation of a hydroxyl adsorbate, *OH, on-top (ot) of Ir_{cus} (abbreviated $*_{cus}$) reads:

$$*_{cus} + H_2O_{(l)} \rightarrow *_{cus}\text{-}OH_{ot} + H^+_{(aq)} + e^- \quad (3)$$

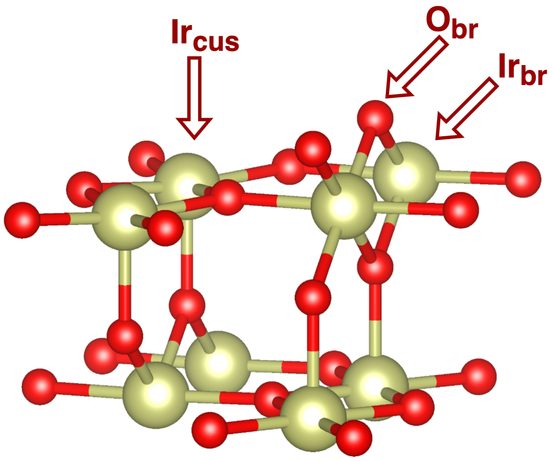

Figure S1. Two uppermost layers of the stochiometric (2x1) $IrO_2(110)$ surface. The surface comprises two distinct types of Ir atoms, cus and bridge sites. In the stoichiometric $IrO_2(110)$ surface, cus sites are unoccupied, while bridge sites are oxygen-covered. This is labeled as Ir_{cus} (short: $*_{cus}$) and O_{br}, respectively.

As the determination of the free energy of liquid water by DFT calculations is error-prone, we use gaseous water at T = 298.15 K and p = 0.035 bar as a reference state. This is justified by referring to the equilibrium between water vapor and liquid water at the specified temperature and pressure. Following the formalism of the CHE approach, the reference state for a proton-electron pair at U = 0 V vs. SHE (standard hydrogen electrode), pH = 0, T = 298.15, and p_{H2} = 1 atm refers to gaseous hydrogen by referring to the equilibrium of equation (4):

$$H^{+}_{(aq)} + e^{-} \rightleftharpoons \frac{1}{2} H_{2(g)} \quad (4)$$

Hence, it is sufficient to calculate a hydrogen molecule by DFT as the chemical potentials of the hydrated proton and the electron are elegantly connected to the free energy of H_2:

$$\mu(H^{+}_{(aq)}) + \mu(e^{-}) = \frac{1}{2} G(H_{2(g)}) \quad (5)$$

2.2.2 Determination of adsorption free energies

Calculating the Gibbs free-energy change (ΔG) for an adsorption process such as specified in equation (3) is paramount for the construction of surface Pourbaix diagrams. In case of several adsorbates in the (2x1) unit cell, the respective equations are simply combined; e. g., for the formation of *OH and *O on-top of two adjacent Ir_{cus} sites, we arrive at:

$$2\ *_{cus} + 2\ H_2O_{(l)} \rightarrow *_{cus}\text{-}OH_{ot} + *_{cus}\text{-}O_{ot} + 3\ H^{+}_{(aq)} + 3\ e^{-} \quad (6)$$

The corresponding ΔG value for the adsorption processes reads:

$$\Delta G(0,0) = G(*_{cus}\text{-}OH_{ot} + *_{cus}\text{-}O_{ot}) + 1.5\ G(H_2) - G(2\ *_{cus}) - 2\ G(H_2O) \quad (7)$$

The term (0,0) in equation (7) indicates that this ΔG value refers to zero electrode potential and zero pH. For all possible adsorbate structures, $\Delta G(0,0)$ values can be obtained by DFT calculations when evaluating equation (8):

$$\Delta G(0,0) = \Delta E_{\text{tot}} + \Delta E_{\text{ZPE}} - T\Delta S \tag{8}$$

Here, ΔE_{tot} denotes the difference in the electronic energy between the adsorbate-covered surface and the stoichiometric $IrO_2(110)$ surface including the respective reference states:

$$\Delta E_{\text{tot}} = E(*_{\text{cus}}\text{-OH}_{\text{ot}} + *_{\text{cus}}\text{-O}_{\text{ot}}) + 1.5\,E(\text{H}_2) - E(2\,*_{\text{cus}}) - 2\,E(\text{H}_2\text{O}) \tag{9}$$

ΔE_{ZPE} indicates the difference in the zero-point energy between the adsorbate-covered surface and the stoichiometric $IrO_2(110)$ surface including the respective reference states. This term is determined by frequency calculations for the reference molecules and the surface slabs where the two bottom two layers were fixed and the uppermost three layers and adsorbates are allowed to freely relax:

$$\Delta ZPE = ZPE(*_{\text{cus}}\text{-OH}_{\text{ot}} + *_{\text{cus}}\text{-O}_{\text{ot}}) + 1.5\,ZPE(\text{H}_2) - ZPE(2\,*_{\text{cus}}) - 2\,ZPE(\text{H}_2\text{O}) \tag{10}$$

Finally, the term $T\Delta S$ refers to the entropic change upon adsorption. For the $IrO_2(110)$ surface and intermediates, the vibrational entropy, S_{vib}, of adsorbates is determined by evaluating equation (11):

$$TS_{\text{vib}} = k_B N_A T \sum_{\text{i}}^{\text{n}} \frac{hv_i/k_BT}{e^{hv_i/k_BT}-1} - \ln\left(1 - e^{-hv_i/k_BT}\right) \tag{11}$$

For the reference molecules water and gaseous hydrogen, the standard entropies, S^0, are taken from thermodynamic data tables.[15] We obtain for the change in entropy during the adsorption process:

$$\Delta S = S_{\text{vib}}(*_{\text{cus}}\text{-OH}_{\text{ot}} + *_{\text{cus}}\text{-O}_{\text{ot}}) + 1.5\,S^0(\text{H}_2) - S_{\text{vib}}(2\,*_{\text{cus}}) - 2\,S^0(\text{H}_2\text{O}) \tag{12}$$

By counting the number of transferred protons, $v(\text{H}^+)$, and electrons, $v(\text{e}^-)$, in each adsorption process, it is possible to translate the $\Delta G(0,0)$ values to potential-dependent free-energy changes:

$$\Delta G(pH, U) = \Delta G(0,0) - v(\text{H}^+)\,k_BT(\ln 10)pH - v(e^-)eU \tag{13}$$

For instance, for the formation of the $*_{\text{cus}}$-OH_{ot} + $*_{\text{cus}}$-O_{ot} phase in equation (6), we infer:

$$\Delta G(pH, U) = \Delta G(0,0) - 3\,k_BT(\ln 10)pH - 3eU \tag{14}$$

In equations (13) and (14), e and k_B denote the elementary charge and Boltzmann's constant, respectively. Note that the term $k_BT(\ln 10)$ amounts to 0.059 V for $T = 298.15$ K.

By minimizing the $\Delta G(pH, U)$ values for all possible adsorbate configurations of the (2x1) $IrO_2(110)$ surface, the energetically preferred surface phase as a function of U and pH is derived, which is depicted in the Pourbaix diagram (cf. Figure 1 in the main text).

2.2.3 SHE vs. RHE: A matter of convenience

While the standard hydrogen electrode (SHE) is often used as the reference for the applied electrode potential, U, in Pourbaix diagrams, we point out that it is possible to translate equation (13) to the RHE (reversible hydrogen electrode) scale when considering the following correlation:

$$U_{\mathrm{SHE}} = U_{\mathrm{RHE}} + k_{\mathrm{B}}T(\ln 10)\, pH \tag{15}$$

Therefore, we obtain for the evaluation of free-energy changes for adsorption processes:

$$\Delta G(U_{\mathrm{RHE}}) = \Delta G(0,0) - \nu(e^{-})eU_{\mathrm{RHE}} \tag{16}$$

Note that this transformation is only reasonable if the adsorbate structures formed reveal the same number of transferred protons, $\nu(H^{+})$, and electrons, $\nu(e^{-})$, in the adsorption process. This prerequisite is obviously met for all OER adsorbates (*OH, *O, *OOH, and *O), and to this end we discuss the Pourbaix diagrams and reaction mechanisms on the RHE rather than the SHE scale.

2.3 Pourbaix diagram of $IrO_2(110)$ under anodic reaction conditions

We construct surface Pourbaix diagrams for the $IrO_2(110)$ surface under OER conditions by utilizing in-house scripts[28] that facilitate the analysis of the free-energy changes for various surface configurations as obtained by equation (16). All surface structures considered in this work including their free-energy changes are listed in Table S1. To account for a comparison between different exchange correlation functionals, we report the free energies for both the PBE and RPBE representations.

Table S1. Free-energy changes, $\Delta G(0,0)$, of different surface configurations with respect to the reference structure $2O_{br} + 2*_{cus}$ using the PBE and RPBE functionals.

	**Structure**	**Δ*G*(0,0) [eV]**	**Δ*G*(0,0) [eV]**
		**PBE**	**RPBE**
	$2O_{br} + 2*_{cus}$ **Reference**	0	0
1	$2OH_{br} + 2*_{cus}\text{-}OH_{ot}$	-2.59	-2.19
2	$2OH_{br} + 1*_{cus}\text{-}OH_{ot}\ 1*_{cus}\text{-}O_{ot}$	-0.96	-0.56

3	$1OH_{br}1O_{br} + 2*_{cus}$-$OH_{ot}$	-1.55	-1.15
4	$2OH_{br} + 2*_{cus}$-O_{ot}	0.71	1.12
5	$1OH_{br}1O_{br} + 1*_{cus}$-$OH_{ot}$ $1*_{cus}$-O_{ot}	0.11	0.50
6	$2O_{br} + 2*_{cus}$-OH_{ot}	-0.33	0.07
7	$1OH_{br}1O_{br} + 2*_{cus}$-$O_{ot}$	1.80	2.19
8	$2O_{br} + 1*_{cus}$-OH_{ot} $1*_{cus}$-O_{ot}	1.43	1.83
9	$2O_{br} + 2*_{cus}$-O_{ot}	3.09	3.55
10	$2O_{br} + 1*_{cus}$-O_{ot} $1*_{cus}$-OOH_{ot}	4.42	5.03
11	$1O_{br}1OOH_{br} + 1*_{cus}$-$O_{ot}$ $1*_{cus}$-OOH_{ot}	H moves to $1*_{cus}$-O_{ot}	H moves to $1*_{cus}$-O_{ot}
12	$1O_{br}1OOH_{br} + 2*_{cus}$-$O_{ot}$	5.09	5.68
13	$2O_{br} + 2*_{cus}$-OOH_{ot}	6.62	7.51
14	$2OOH_{br} + 2*_{cus}$-O_{ot}	7.36	8.17
15	$1O_{br}1OOH_{br} + 2*_{cus}$-$OOH_{ot}$	7.94	9.09
16	$2OOH_{br} + 1*_{cus}$-O_{ot} $1*_{cus}$-OOH_{ot}	H moves to $1*_{cus}$-O_{ot}	H moves to $1*_{cus}$-O_{ot}
17	$2OOH_{br} + 2*_{cus}$-OOH_{ot}	11.01	12.40
18	$2OH_{br} + 2*_{cus}$	-1.59	-1.72

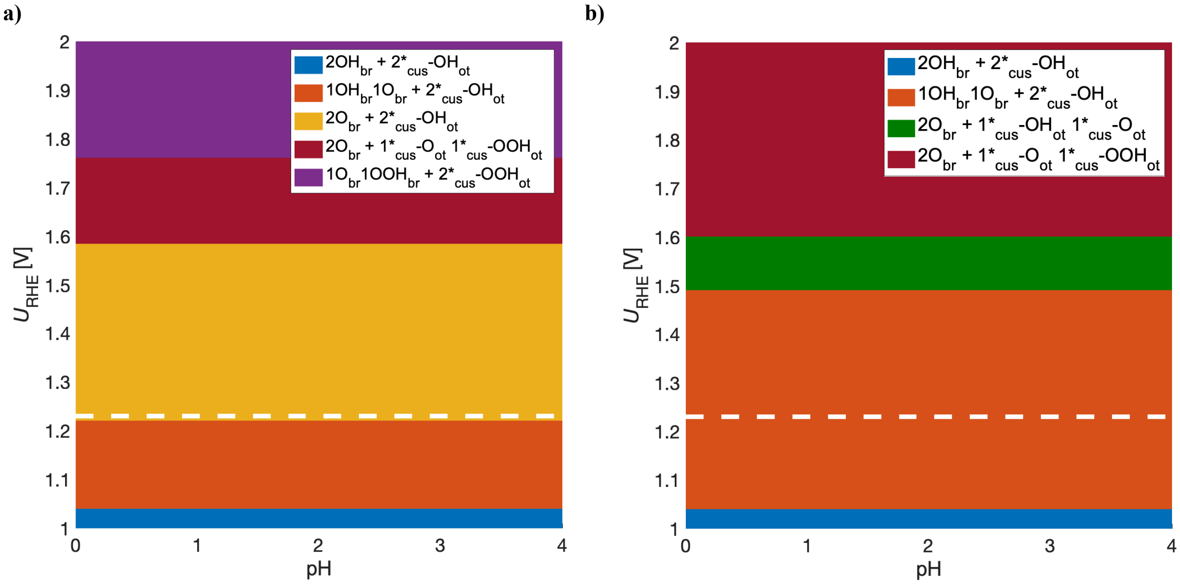

Figure S2. Pourbaix diagram for a IrO_2(110) surface under anodic reaction conditions. Only the thermodynamically preferred surface phase with the lowest free energy is indicated. The white dotted line represents the equilibrium potential of the OER; that is, U^0_{OER} = 1.23 V vs. RHE. Panels a) and b) refer to the PBE and RPBE functionals, respectively.

Figure S2 illustrates that there are only minor quantitative differences in the preferred surface structure when employing the PBE or RPBE functionals in the DFT calculations. Various surface configurations turn out to be stable under anodic conditions, ranging from a fully hydroxylated surface ($2OH_{br}$ + $2*_{cus}$-OH_{ot}) to partially hydroxylated surfaces ($1OH_{br}$ $1O_{br}$ + $2*_{cus}$-OH_{ot} or $2O_{br}$ + $2*_{cus}$-OH_{ot}) or partially OOH-covered surfaces ($2O_{br}$ + $1*_{cus}$-OOH_{ot} $1*_{cus}$-O_{ot} or $1O_{br}$ $1OOH_{br}$ + $2*_{cus}$-OOH_{ot}). The respective potential range where these surfaces are energetically preferred can vary up to about 200 mV on the RHE scale between the two different functionals, which is still within the error bars of conventional DFT approaches.

To this end, we consider multiple surface structures, ranging from hydroxylated to oxygen-covered, and OOH-covered surfaces (cf. section 3 of the SI or Figure 1b-e in the main text) to comprehend the elementary steps of the OER over $IrO_2(110)$. All further calculations were performed with the PBE functional as the above comparison of the Pourbaix diagrams reveals that there are no qualitative differences between the PBE and RPBE descriptions.

3 Mechanistic Pathways for Oxygen Evolution Reaction (OER)

In our mechanistic study, we factor a variety of different reaction mechanisms into the analysis, following the previous work by one of the authors.[29] Reaction equations for the elementary steps of each pathway are provided in the following.

3.1 Mononuclear mechanism

The mononuclear mechanism consists of the subsequent formation of the *OH, *O, and *OOH intermediates:[19,20]

$$*_{cus} + H_2O \rightarrow *_{cus}\text{-}OH_{ot} + H^+ + e^- \quad (17)$$

$$*_{cus}\text{-}OH_{ot} \rightarrow *_{cus}\text{-}O_{ot} + H^+ + e^- \quad (18)$$

$$*_{cus}\text{-}O_{ot} + H_2O \rightarrow *_{cus}\text{-}OOH_{ot} + H^+ + e^- \quad (19)$$

$$*_{cus}\text{-}OOH_{ot} \rightarrow *_{cus} + O_{2(g)} + H^+ + e^- \quad (20)$$

3.2 Bifunctional I mechanism

The bifunctional I mechanism differs from the mononuclear mechanism in that instead of the *OOH adsorbate, the *OO intermediate is formed by transferring one proton to a neighboring cus or bridge site.[22]

$$*_{cus} + O_{br} + H_2O \rightarrow *_{cus}\text{-}OH_{ot} + O_{br} + H^+ + e^- \quad (21)$$

$$*_{cus}\text{-}OH_{ot} + O_{br} \rightarrow *_{cus}\text{-}O_{ot} + O_{br} + H^+ + e^- \quad (22)$$

$$*_{cus}\text{-}O_{ot} + O_{br} + H_2O \rightarrow *_{cus}\text{-}OO_{ot} + OH_{br} + H^+ + e^- \quad (23)$$

$$*_{cus}\text{-}OO_{ot} + OH_{br} \rightarrow *_{cus} + O_{br} + O_{2(g)} + H^+ + e^- \quad (24)$$

3.3 Bifunctional II mechanism

In contrast to the bifunctional I mechanism, the bifunctional II mechanism[21,23,24] consists of a chemical reaction step, in which the second water molecule is adsorbed to the surface without the direct release of a proton-electron pair:

$$*_{cus} + O_{br} + H_2O \rightarrow *_{cus}\text{-}OH_{ot} + O_{br} + H^+ + e^- \quad (25)$$

$$*_{cus}\text{-}OH_{ot} + O_{br} \rightarrow *_{cus}\text{-}O_{ot} + O_{br} + H^+ + e^- \quad (26)$$

$$*_{cus}\text{-}O_{ot} + O_{br} + H_2O \rightarrow *_{cus}\text{-}OOH_{ot} + OH_{br} \quad (27)$$

$$*_{cus}\text{-}OOH_{ot} + OH_{br} \rightarrow *_{cus}\text{-}OOH_{ot} + O_{br} + H^+ + e^- \quad (28)$$

$$*_{cus}\text{-}OOH_{ot} + O_{br} \rightarrow *_{cus} + O_{br} + O_{2(g)} + H^+ + e^- \quad (29)$$

3.4 Oxide mechanism

The oxide mechanism[27] commences from two adjacent oxygen adsorbates (*O), and gaseous oxygen is formed by the chemical recombination of the outermost oxygen atoms of two adjacent *OO intermediates:

$$*_{cus}\text{-}O_{ot}\ *_{cus}\text{-}O_{ot} + H_2O \rightarrow *_{cus}\text{-}OOH_{ot}\ *_{cus}\text{-}O_{ot} + H^+ + e^- \quad (30)$$

$$*_{cus}\text{-}OOH_{ot}\ *_{cus}\text{-}O_{ot} + H_2O \rightarrow *_{cus}\text{-}OOH_{ot}\ *_{cus}\text{-}OOH_{ot} + H^+ + e^- \quad (31)$$

$$*_{cus}\text{-}OOH_{ot}\ *_{cus}\text{-}OOH_{ot} \rightarrow *_{cus}\text{-}OOH_{ot}\ *_{cus}\text{-}OO_{ot} + H^+ + e^- \quad (32)$$

$$*_{cus}\text{-}OOH_{ot}\ *_{cus}\text{-}OO_{ot} \rightarrow *_{cus}\text{-}OO_{ot}\ *_{cus}\text{-}OO_{ot} + H^+ + e^- \quad (33)$$

$$*_{cus}\text{-}OO_{ot}\ *_{cus}\text{-}OO_{ot} \rightarrow *_{cus}\text{-}O_{ot}\ *_{cus}\text{-}O_{ot} + O_{2(g)} \quad (34)$$

3.5 Binuclear mechanism

In the binuclear mechanism,[25,26] two adjacent oxygen adsorbates (*O) recombine by a chemical step to release the product gaseous oxygen:

$$*_{cus} + *_{cus} + H_2O \rightarrow *_{cus}\text{-}OH_{ot} + *_{cus} + H^+ + e^- \quad (35)$$

$$*_{cus}\text{-}OH_{ot} + *_{cus} + H_2O \rightarrow *_{cus}\text{-}OH_{ot} + *_{cus}\text{-}OH_{ot} + H^+ + e^- \quad (36)$$

$$*_{cus}\text{-}OH_{ot} + *_{cus}\text{-}OH_{ot} \rightarrow *_{cus}\text{-}O_{ot} + *_{cus}\text{-}OH_{ot} + H^+ + e^- \quad (37)$$

$$*_{cus}\text{-}O_{ot} + *_{cus}\text{-}OH_{ot} \rightarrow *_{cus}\text{-}O_{ot} + *_{cus}\text{-}O_{ot} + H^+ + e^- \quad (38)$$

$$*_{cus}\text{-}O_{ot} + *_{cus}\text{-}O_{ot} \rightarrow *_{cus} + *_{cus} + O_{2(g)} \quad (39)$$

The above pathways are modeled for the following four surface configurations of $IrO_2(110)$:

a) Fully hydroxylated surface ($2OH_{br} + 2*_{cus}\text{-}OH_{ot}$)
b) Partly hydroxylated surface ($2O_{br} + 2*_{cus}\text{-}OH_{ot}$)
c) Fully oxygen-covered surface ($2O_{br} + 2*_{cus}\text{-}O_{ot}$)
d) Partly OOH-covered surface ($2O_{br} + 1*_{cus}\text{-}OOH_{ot}$ $1*_{cus}\text{-}O_{ot}$)

This choice is based on the Pourbaix diagram of Figure S2 as the free-energy differences between the respective phases can be less than 0.1 eV in the potential window of U = 1.4 – 1.6 V vs. RHE, indicating that all these phases can potentially contribute to the formation of gaseous oxygen during the OER. A sketch of the uppermost layer for the fully oxygen-covered (2x1) $IrO_2(110)$ surface is given in Figure S3.

Electrocatalytic activity for each pathway is assessed by the activity descriptor $G_{max}(U)$ as introduced in a recent contribution.[30,31] While the most popular activity descriptor in the electrocatalysis community refers to the thermodynamic overpotential, η_{TD},[15,32] the difference between η_{TD} and $G_{max}(U)$ refers to the fact that η_{TD} renders activity predictions at the equilibrium potential of an electrocatalytic process whereas $G_{max}(U)$ allows potential-dependent activity analyses. Due to relying on the concept of a free-energy span model,[33] $G_{max}(U)$ captures overpotential and kinetic effects at least to a qualitative extent in the thermodynamic evaluation of adsorption free energies. In addition, this descriptor is further complemented with a measure for sensitivity: only if two pathways reveal a difference in $G_{max}(U)$ of at least by 200 meV at a fixed electrode potential, it can be concluded unambiguously that the pathway with the lower $G_{max}(U)$ is operative.[30] This is the reason why we adopt the concept of $G_{max}(U)$ rather than the notion of η_{TD} in our mechanistic analysis for the OER over $IrO_2(110)$.

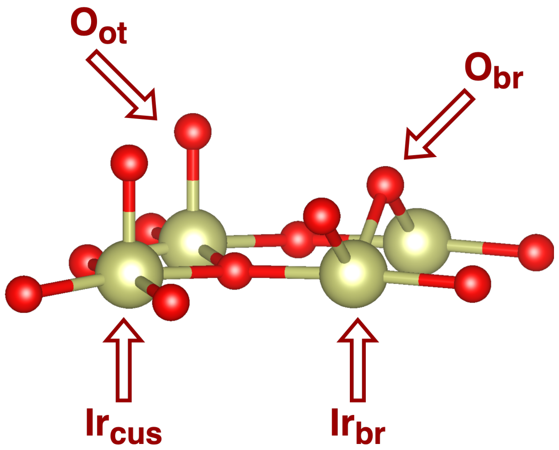

Figure S3. Uppermost layer of the fully oxygen-covered (2x1) $IrO_2(110)$ surface. Note that this representation of the surface is used in the following for the mechanistic discussion of the various pathways.

4 OER over the fully hydroxylated $IrO_2(110)$ surface

4.1 Mononuclear Mechanism

The mononuclear mechanism comprises the formation of the *OH (step 1), *O (step 2), and *OOH (step 3) intermediates, which is followed by the subsequent release of the product O_2 (step 4). Table S2 compiles the free-energy changes for each elementary step at $U = 0$ V vs. RHE and the activity descriptor $G_{max}(U)$ at different applied electrode potentials under OER conditions. The corresponding free-energy diagram is depicted in Figure S4a, with a visual representation of the elementary steps in Figure S4b. In the potential range of 1.23 V to 1.53 V vs. RHE, the limiting free-energy span is reconciled with $*_{cus}\text{-}OH_{ot} \rightarrow *_{cus}\text{-}O_{ot} \rightarrow *_{cus}\text{-}OOH_{ot} \rightarrow *_{cus} + O_2$ due to the strong adsorption of the *OH intermediate.

Table S2. Energetic evaluation of the mononuclear mechanism on the fully hydroxylated $IrO_2(110)$ surface (cf. Figure 1b in the main text) by the framework of the descriptor $G_{max}(U)$. The table indicates the free-energy changes of each step at $U = 0$ V vs. RHE and $G_{max}(U)$ values at different applied electrode potentials (U).

ΔG_1 [eV]	ΔG_2 [eV]	ΔG_3 [eV]	ΔG_4 [eV]	$G_{max}(U)$ [eV]				
				**1.23 V**	**1.33 V**	**1.43 V**	**1.53 V**	**1.63 V**
-0.53	1.65	1.44	2.36	1.76	1.46	1.16	0.86	0.73

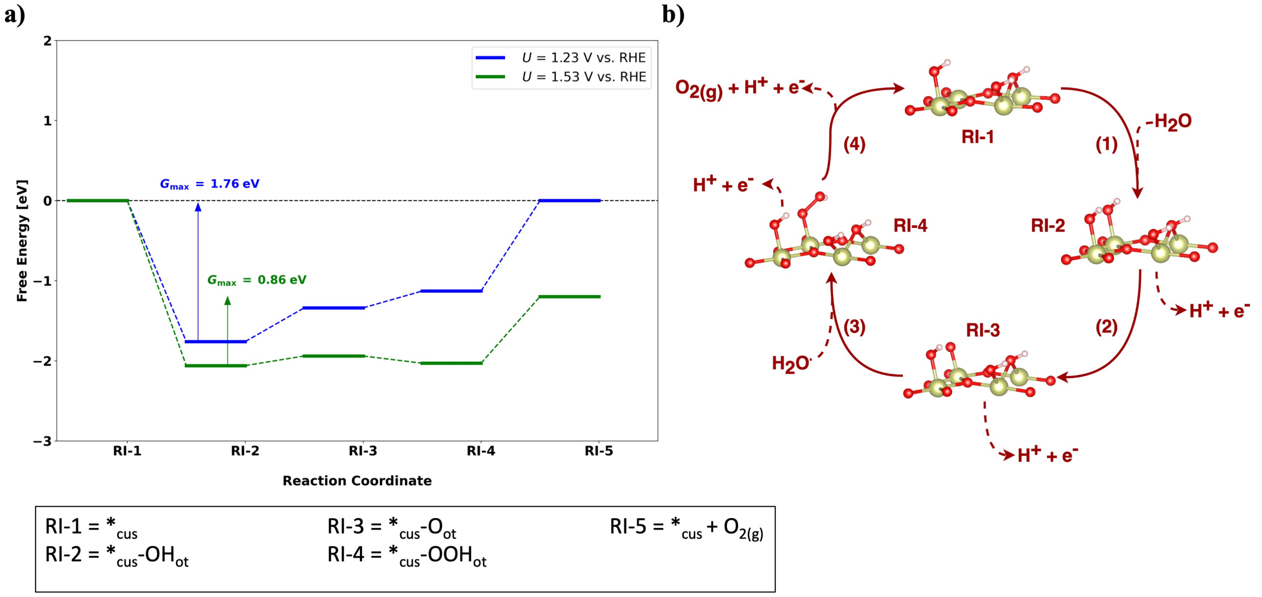

Figure S4. a) Free-energy diagram for the mononuclear mechanism on the fully hydroxylated $IrO_2(110)$ surface at 1.23 V and 1.53 V vs. RHE. The reaction intermediates of the mechanistic cycle are labeled on the x-axis. Blue and green solid lines indicate intermediates' free energies at 1.23 V and 1.53 V, respectively. Colored arrows indicate the free-energy span governing $G_{max}(U)$, with the respective value displayed.
b) Schematic illustration of the mononuclear mechanism, as described in Section 3.1, on the fully hydroxylated $IrO_2(110)$ surface. Numbers next to the arrows indicate the step sequence, and each structure represents the corresponding reaction intermediate.

4.2 Bifunctional I mechanism

While the first two steps of the bifunctional I mechanism are identical to the mononuclear pathway, the third and fourth steps differ compared to the mononuclear description (cf. section 3.2 or Figure S5b). Table S3 compiles the free-energy changes for each elementary step at $U = 0$ V vs. RHE and the activity descriptor $G_{max}(U)$ at different applied electrode potentials under OER conditions. The corresponding free-energy diagram is depicted in Figure S5a. Similar to the mononuclear mechanism, the descriptor $G_{max}(U)$ is governed by the span $*_{cus}$-OH_{ot} + O_{br} → $*_{cus}$-O_{ot} + O_{br} → $*_{cus}$-OO_{ot} + OH_{br} → $*_{cus}$ + O_{br} + O_2 in the potential range of 1.23 V to 1.53 V vs. RHE.

Table S3. Energetic evaluation of the bifunctional I mechanism on the fully hydroxylated $IrO_2(110)$ surface (cf. Figure 1b in the main text) by the framework of the descriptor $G_{max}(U)$. The table indicates the free-energy changes of each step at $U = 0$ V vs. RHE and $G_{max}(U)$ values at different applied electrode potentials (U).

**ΔG_1** [eV]	**ΔG_2** [eV]	**ΔG_3** [eV]	**ΔG_4** [eV]	**$G_{max}(U)$** [eV]				
				**1.23 V**	**1.33 V**	**1.43V**	**1.53 V**	**1.63 V**
-0.24	1.66	1.42	2.09	1.47	1.17	0.87	0.57	0.46

a)

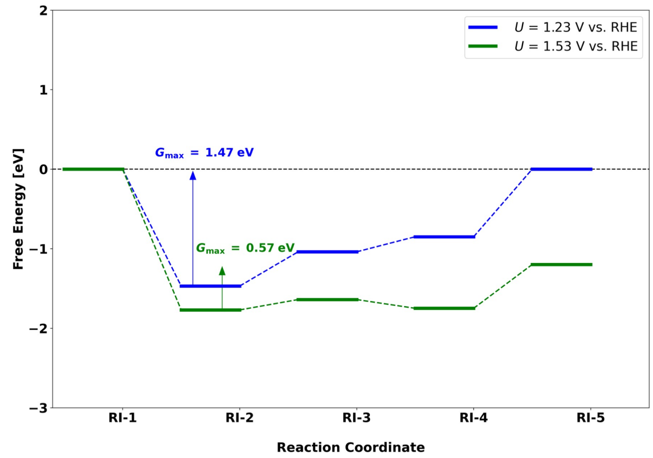

b)

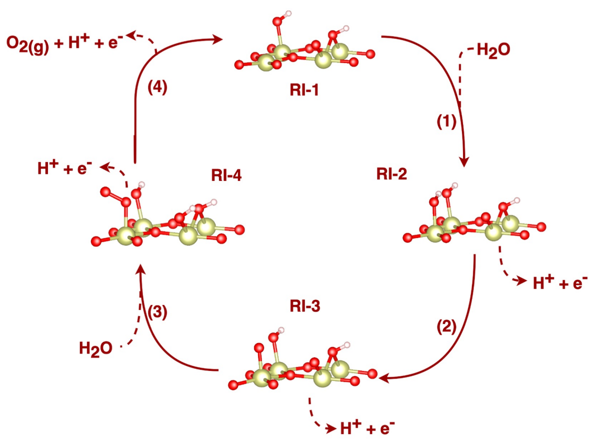

RI-1 = $*_{cus}$ + O_{br} RI-3 = $*_{cus}$-O_{ot} + O_{br} RI-5 = $*_{cus}$ + O_{br} + $O_{2(g)}$
RI-2 = $*_{cus}$-OH_{ot} + O_{br} RI-4 = $*_{cus}$-OO_{ot} + OH_{br}

Figure S5. a) Free-energy diagram for the bifunctional I mechanism on the fully hydroxylated $IrO_2(110)$ surface at 1.23 V and 1.53 V vs. RHE. The reaction intermediates of the mechanistic cycle are labeled on the x-axis. Blue and green solid lines indicate intermediates' free energies at 1.23 V and 1.53 V, respectively. Colored arrows indicate the free-energy span governing $G_{max}(U)$, with the respective value displayed.
b) Schematic illustration of the bifunctional I mechanism, as described in Section 3.2, on the fully hydroxylated $IrO_2(110)$ surface. Numbers next to the arrows indicate the step sequence, and each structure represents the corresponding reaction intermediate.

4.3 Bifunctional II mechanism

In the bifunctional II mechanism, the *OOH intermediate is formed by a chemical rather than an electrochemical reaction step (cf. section 3.3 or Figure S6b). Table S4 compiles the free-energy changes for each elementary step at U = 0 V vs. RHE and the activity descriptor $G_{max}(U)$ at different applied electrode potentials under OER conditions. The corresponding free-energy diagram is depicted in Figure S6a. Similar to the mononuclear mechanism, the descriptor $G_{max}(U)$ is governed by the span $*_{cus}$-OH_{ot} + O_{br} → $*_{cus}$-O_{ot} + O_{br} → $*_{cus}$-OOH_{ot} + OH_{br} → $*_{cus}$-OOH_{ot} + O_{br} → $*_{cus}$ + O_{br} + O_2 in the potential range of 1.23 V to 1.53 V vs. RHE.

Table S4. Energetic evaluation of the bifunctional II mechanism on the fully hydroxylated $IrO_2(110)$ surface (cf. Figure 1b in the main text) by the framework of the descriptor $G_{max}(U)$. The table indicates the free-energy changes of each step at U = 0 V vs. RHE and $G_{max}(U)$ values at different applied electrode potentials (U).

ΔG_1 [eV]	ΔG_2 [eV]	ΔG_3 [eV]	ΔG_4 [eV]	ΔG_5 [eV]	$G_{max}(U)$ [eV]				
					**1.23 V**	**1.33 V**	**1.43 V**	**1.53 V**	**1.63 V**
-0.24	1.66	0.39	1.01	2.11	1.47	1.17	0.87	0.57	0.48

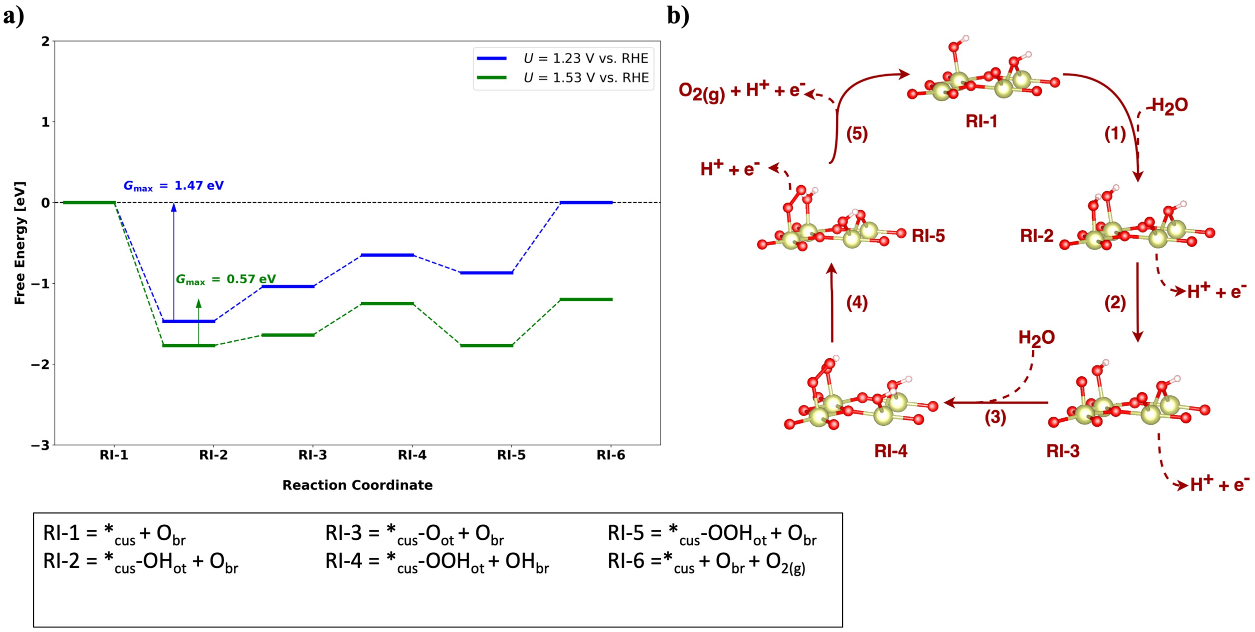

Figure S6. a) Free-energy diagram for the bifunctional II mechanism on the fully hydroxylated $IrO_2(110)$ surface at 1.23 V and 1.53 V vs. RHE. The reaction intermediates of the mechanistic cycle are labeled on the x-axis. Blue and green solid lines indicate intermediates' free energies at 1.23 V and 1.53 V, respectively. Colored arrows indicate the free-energy span governing $G_{max}(U)$, with the respective value displayed.
b) Schematic illustration of the bifunctional II mechanism, as described in Section 3.3, on the fully hydroxylated $IrO_2(110)$ surface. Numbers next to the arrows indicate the step sequence, and each structure represents the corresponding reaction intermediate.

4.4 Oxide Mechanism

The oxide mechanism requires complete oxygen coverage at the cus sites (cf. section 3.4). Even if we consider that initially, the *OH adsorbates are transformed to *O, we observe that one of

the intermediate structures in the oxide pathway containing the *OOH adsorbate is unstable. Therefore, we conclude that the oxide mechanism is unlikely for the fully hydroxylated $IrO_2(110)$ surface.

4.5 Binuclear Mechanism

The binuclear mechanism consists of the chemical recombination of two neighboring *O adsorbates (cf. section 3.5 or Figure S7b). Table S5 compiles the free-energy changes for each elementary step at U = 0 V vs. RHE and the activity descriptor $G_{max}(U)$ at different applied electrode potentials under OER conditions. The corresponding free-energy diagram is depicted in Figure S7a. Similar to the mononuclear mechanism, the descriptor $G_{max}(U)$ is governed by the free-energy span of the *OH intermediate to the product O_2 in the potential range of 1.23 V to 1.53 V vs. RHE.

Table S5. Energetic evaluation of the binuclear mechanism on the fully hydroxylated $IrO_2(110)$ surface (cf. Figure 1b in the main text) by the framework of the descriptor $G_{max}(U)$. The table indicates the free-energy changes of each step at U = 0 V vs. RHE and $G_{max}(U)$ values at different applied electrode potentials (U).

ΔG_1 [eV]	ΔG_2 [eV]	ΔG_3 [eV]	ΔG_4 [eV]	ΔG_5 [eV]	$G_{max}(U)$ [eV]				
					**1.23 V**	**1.33 V**	**1.43 V**	**1.53 V**	**1.63 V**
-0.47	-0.53	1.65	1.65	2.62	3.46	3.26	3.06	2.86	2.66

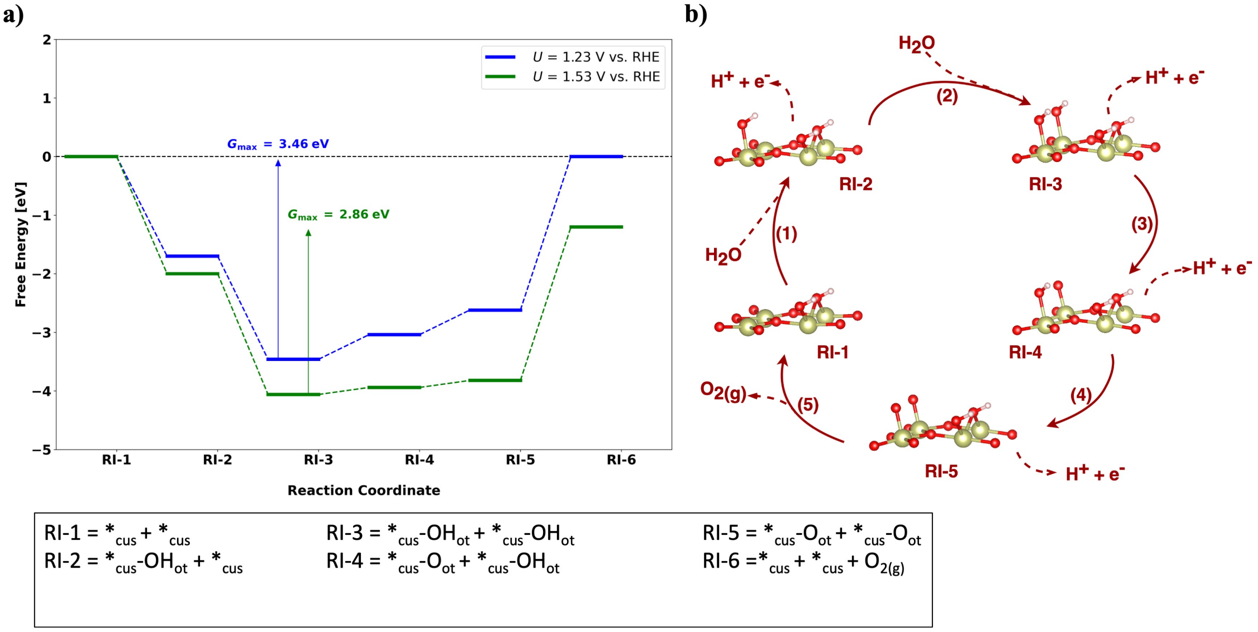

Figure S7. a) Free-energy diagram for the binuclear mechanism on the fully hydroxylated $IrO_2(110)$ surface at 1.23 V and 1.53 V vs. RHE. The reaction intermediates of the mechanistic cycle are labeled on the x-axis. Blue and green solid lines indicate intermediates' free energies at 1.23 V and 1.53 V, respectively. Colored arrows indicate the free-energy span governing $G_{max}(U)$, with the respective value displayed.

b) Schematic illustration of the binuclear mechanism, as described in Section 3.5, on the fully hydroxylated $IrO_2(110)$ surface. Numbers next to the arrows indicate the step sequence, and each structure represents the corresponding reaction intermediate.

4.6 Assessment of solvation effects by VASPsol

Electrocatalytic reactions such as the OER take place at electrified solid/ liquid interfaces, indicating that the aqueous solvent may have a non-negligible effect on the energetics of the electrochemical process. Despite this, there is no unifying framework of how to account for the surrounding aqueous phase in DFT calculations. While Chan and coworkers[34] reported that the application of continuum solvation methods do not necessarily provide more accurate energetics than gas-phase DFT calculations, we qualitatively assess the impact of solvation on the mononuclear OER by an implicit description as implemented in the VASPsol package.[10,11] Table S6 compiles the calculated free-energy changes neglecting or including solvation, their differences, and the difference in the activity descriptor $G_{max}(U)$. While the quantitative impact of solvation on the free-energy changes does not exceed 0.20 eV, it is evident that the value of the activity descriptor $G_{max}(U)$ remains virtually constant. This finding suggests that, for the fully hydroxylated IrO_2(110) surface, gas-phase DFT calculations provide a reliable energetic representation of the OER.

Table S6. Comparing the energetics of gas-phase DFT calculations and DFT including implicit solvation by means of VASPsol for the elementary steps of the mononuclear OER mechanism on the fully hydroxylated IrO_2(110) surface. Note that $\Delta\Delta G_j$ (j = 1, 2, 3, 4) indicates the free-energy difference of the free-energy changes for the continuum solvation approach and gas-phase DFT. Likewise, $\Delta G_{max}(U)$ denotes the difference in the activity descriptor $G_{max}(U)$ for the continuum solvation approach and gas-phase DFT.

	ΔG_1 [eV]	ΔG_2 [eV]	ΔG_3 [eV]	ΔG_4 [eV]
**Gas-phase DFT**	-0.53	1.65	1.44	2.36
**DFT + VASPsol**	-0.50	1.46	1.61	2.35

$\Delta\Delta G_j = \Delta G_{j;\,VASPsol} - \Delta G_{j;\,gas}$ [eV]				$\Delta G_{max}(U) = (G_{max}(U))_{VASPsol} - (G_{max}(U))_{gas}$ [eV]				
$\Delta\Delta G_1$	$\Delta\Delta G_2$	$\Delta\Delta G_3$	$\Delta\Delta G_4$	**1.23 V**	**1.33 V**	**1.43 V**	**1.53 V**	**1.63 V**
0.03	-0.19	0.17	-0.01	-0.03	-0.03	-0.03	0.04	-0.01

5 OER over the partly hydroxylated IrO_2(110) surface

5.1 Mononuclear Mechanism

Table S7 compiles the free-energy changes for each elementary step at U = 0 V vs. RHE and the activity descriptor $G_{max}(U)$ at different applied electrode potentials under OER conditions. The corresponding free-energy diagram is depicted in Figure S8a, with a visual representation of the elementary steps in Figure S8b. While the activity descriptor $G_{max}(U)$ is governed by the span

$*_{cus}$-OH_{ot} → $*_{cus}$-O_{ot} → $*_{cus}$-OOH_{ot} → $*_{cus}$ + O_2 at U = 1.23 V vs. RHE, the limiting free-energy span switches to $*_{cus}$-OOH_{ot} → $*_{cus}$ + O_2 for larger applied overpotentials (U = 1.53 V vs. RHE).

Table S7. Energetic evaluation of the mononuclear mechanism on the partly hydroxylated IrO_2(110) surface (cf. Figure 1c in the main text) by the framework of the descriptor $G_{max}(U)$. The table indicates the free-energy changes of each step at U = 0 V vs. RHE and $G_{max}(U)$ values at different applied electrode potentials (U).

ΔG_1 [eV]	ΔG_2 [eV]	ΔG_3 [eV]	ΔG_4 [eV]	$G_{max}(U)$ [eV]				
				**1.23 V**	**1.33 V**	**1.43 V**	**1.53 V**	**1.63 V**
-0.22	1.75	1.11	2.27	1.45	1.15	0.85	0.75	0.65

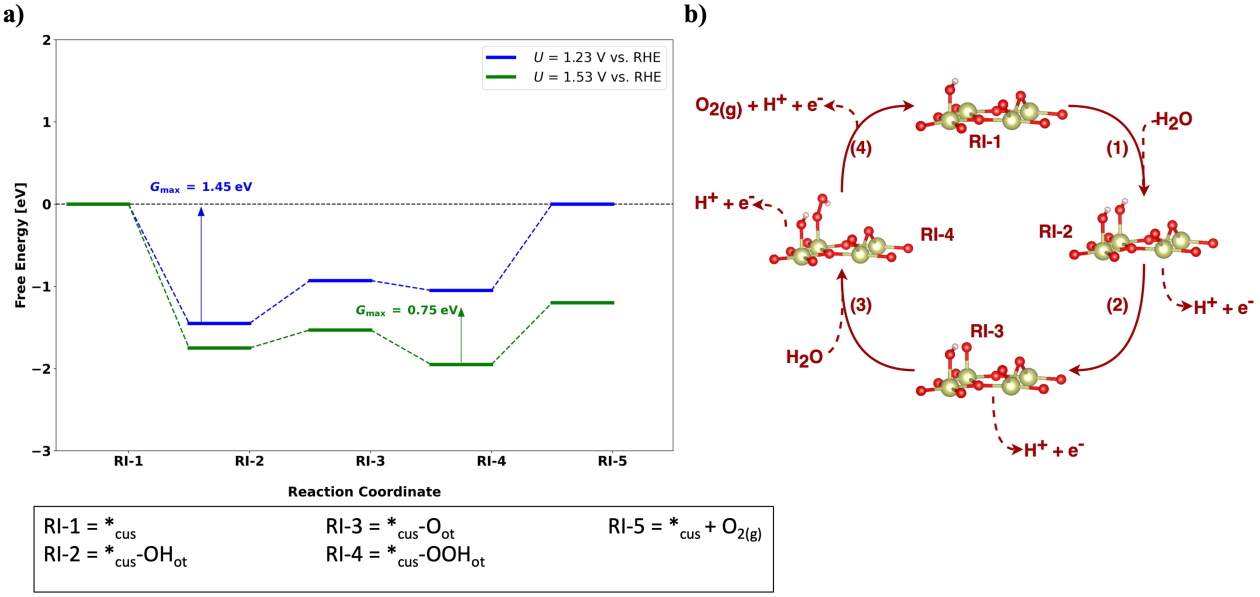

Figure S8. a) Free-energy diagram for the mononuclear mechanism on the partly hydroxylated IrO_2(110) surface at 1.23 V and 1.53 V vs. RHE. The reaction intermediates of the mechanistic cycle are labeled on the x-axis. Blue and green solid lines indicate intermediates' free energies at 1.23 V and 1.53 V, respectively. Colored arrows indicate the free-energy span governing $G_{max}(U)$, with the respective value displayed.
b) Schematic illustration of the mononuclear mechanism, as described in Section 3.1, on the partly hydroxylated IrO_2(110) surface. Numbers next to the arrows indicate the step sequence, and each structure represents the corresponding reaction intermediate.

5.2 Bifunctional I mechanism

Table S8 compiles the free-energy changes for each elementary step at U = 0 V vs. RHE and the activity descriptor $G_{max}(U)$ at different applied electrode potentials under OER conditions. The corresponding free-energy diagram is depicted in Figure S9a. Similar to the mononuclear mechanism, the activity descriptor $G_{max}(U)$ is governed by the span $*_{cus}$-OH_{ot} + O_{br} → $*_{cus}$-O_{ot} + O_{br} → $*_{cus}$-OO_{ot} + OH_{br} → $*_{cus}$ + O_{br} + O_2 at U = 1.23 V vs. RHE, whereas the limiting free-energy span switches to $*_{cus}$-OO_{ot} + OH_{br} → $*_{cus}$ + O_{br} + O_2 for larger applied overpotentials (U = 1.53 V vs. RHE).

Table S8. Energetic evaluation of the bifunctional I mechanism on the partly hydroxylated $IrO_2(110)$ surface (cf. Figure 1c in the main text) by the framework of the descriptor $G_{max}(U)$. The table indicates the free-energy changes of each step at $U = 0$ V vs. RHE and $G_{max}(U)$ values at different applied electrode potentials (U).

ΔG_1 [eV]	ΔG_2 [eV]	ΔG_3 [eV]	ΔG_4 [eV]	$G_{max}(U)$ [eV]				
				**1.23 V**	**1.33 V**	**1.43 V**	**1.53 V**	**1.63 V**
-0.22	1.75	1.18	2.21	1.45	1.15	0.85	0.68	0.58

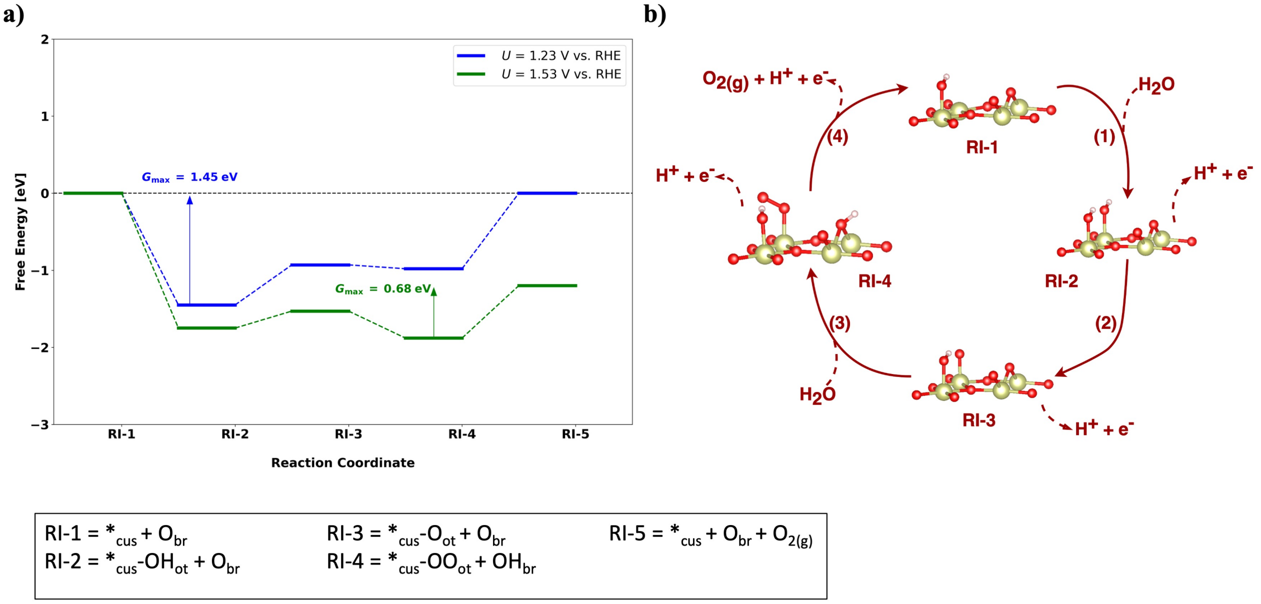

Figure S9. a) Free-energy diagram for the bifunctional I mechanism on the partly hydroxylated $IrO_2(110)$ surface at 1.23 V and 1.53 V vs. RHE. The reaction intermediates of the mechanistic cycle are labeled on the x-axis. Blue and green solid lines indicate intermediates' free energies at 1.23 V and 1.53 V, respectively. Colored arrows indicate the free-energy span governing $G_{max}(U)$, with the respective value displayed.
b) Schematic illustration of the bifunctional I mechanism, as described in Section 3.2, on the partly hydroxylated $IrO_2(110)$ surface. Numbers next to the arrows indicate the step sequence, and each structure represents the corresponding reaction intermediate.

5.3 Bifunctional II mechanism

Table S9 compiles the free-energy changes for each elementary step at $U = 0$ V vs. RHE and the activity descriptor $G_{max}(U)$ at different applied electrode potentials under OER conditions. The corresponding free-energy diagram is depicted in Figure S10a. Similar to the mononuclear mechanism, the activity descriptor $G_{max}(U)$ is governed by the span $*_{cus}\text{-}OH_{ot} + O_{br} \rightarrow *_{cus}\text{-}O_{ot} + O_{br} \rightarrow *_{cus}\text{-}OOH_{ot} + OH_{br} \rightarrow *_{cus}\text{-}OOH_{ot} + O_{br} \rightarrow *_{cus} + O_{br} + O_2$ at $U = 1.23$ V vs. RHE, whereas the limiting free-energy span switches to $*_{cus}\text{-}OOH_{ot} + O_{br} \rightarrow *_{cus} + O_{br} + O_2$ for more anodic conditions ($U = 1.53$ V vs. RHE).

Table S9. Energetic evaluation of the bifunctional II mechanism on the partly hydroxylated $IrO_2(110)$ surface (cf. Figure 1c in the main text) by the framework of the descriptor $G_{max}(U)$. The table indicates the free-energy changes of each step at $U = 0$ V vs. RHE and $G_{max}(U)$ values at different applied electrode potentials (U).

ΔG_1 [eV]	ΔG_2 [eV]	ΔG_3 [eV]	ΔG_4 [eV]	ΔG_5 [eV]	$G_{max}(U)$ [eV]				
					**1.23 V**	**1.33 V**	**1.43 V**	**1.53 V**	**1.63 V**
-0.22	1.75	0.00	1.11	2.28	1.45	1.15	0.85	0.75	0.65

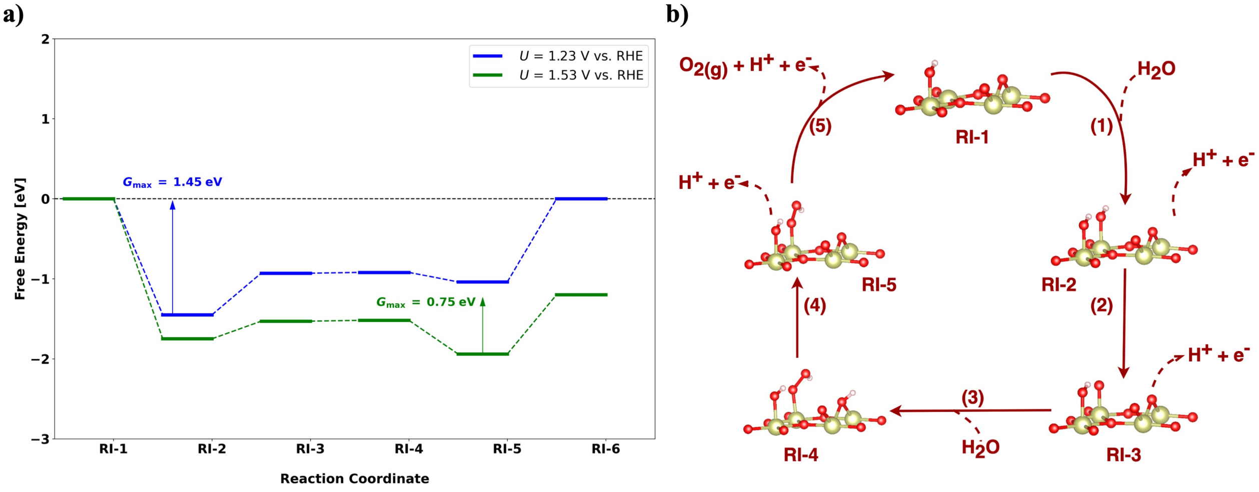

RI-1 = $*_{cus} + O_{br}$
RI-2 = $*_{cus}\text{-}OH_{ot} + O_{br}$
RI-3 = $*_{cus}\text{-}O_{ot} + O_{br}$
RI-4 = $*_{cus}\text{-}OOH_{ot} + OH_{br}$
RI-5 = $*_{cus}\text{-}OOH_{ot} + O_{br}$
RI-6 = $*_{cus} + O_{br} + O_{2(g)}$

Figure S10. a) Free-energy diagram for the bifunctional II mechanism on the partly hydroxylated $IrO_2(110)$ surface at 1.23 V and 1.53 V vs. RHE. The reaction intermediates of the mechanistic cycle are labeled on the x-axis. Blue and green solid lines indicate intermediates' free energies at 1.23 V and 1.53 V, respectively. Colored arrows indicate the free-energy span governing $G_{max}(U)$, with the respective value displayed.
b) Schematic illustration of the bifunctional II mechanism, as described in Section 3.3, on the partly hydroxylated $IrO_2(110)$ surface. Numbers next to the arrows indicate the step sequence, and each structure represents the corresponding reaction intermediate.

5.4 Oxide Mechanism

In contrast to the fully hydroxylated $IrO_2(110)$ surface, we observe that the oxide mechanism (cf. section 3.4 or Figure S11b can take place in a partly hydroxylated environment of the active site. Table S10 compiles the free-energy changes for each elementary step at $U = 0$ V vs. RHE and the activity descriptor $G_{max}(U)$ at different applied electrode potentials under OER conditions. The corresponding free-energy diagram is depicted in Figure S11a. In contrast to the other mechanisms, the activity descriptor $G_{max}(U)$ is governed by the formation rather than the decomposition of the *OOH intermediate from the *O-covered surface.

Table S10. Energetic evaluation of the oxide mechanism on the partly hydroxylated $IrO_2(110)$ surface (cf. Figure 1c in the main text) by the framework of the descriptor $G_{max}(U)$. The table indicates the free-energy changes of each step at $U = 0$ V vs. RHE and $G_{max}(U)$ values at different applied electrode potentials (U).

ΔG_1 [eV]	ΔG_2 [eV]	ΔG_3 [eV]	ΔG_4 [eV]	ΔG_5 [eV]	$G_{max}(U)$ [eV]				
					**1.23 V**	**1.33 V**	**1.43 V**	**1.53 V**	**1.63 V**
1.33	2.19	0.71	0.67	0.01	1.07	0.87	0.76	0.66	0.56

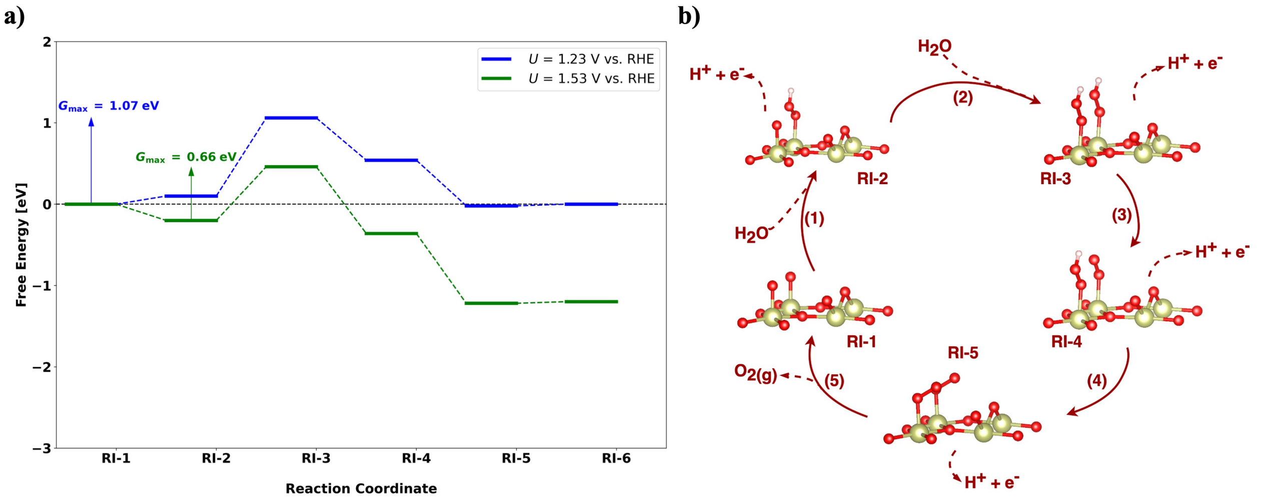

RI-1 = $*_{cus}$-O_{ot} + $*_{cus}$-O_{ot}
RI-2 = $*_{cus}$-OOH_{ot} + $*_{cus}$-O_{ot}
RI-3 = $*_{cus}$-OOH_{ot} + $*_{cus}$-OOH_{ot}
RI-4 = $*_{cus}$-OOH_{ot} + $*_{cus}$-OO_{ot}
RI-5 = $*_{cus}$-OO_{ot} + $*_{cus}$-OO_{ot}
RI-6 = $*_{cus}$-O_{ot} + $*_{cus}$-O_{ot} + $O_{2(g)}$

Figure S11. a) Free-energy diagram for the oxide mechanism on the partly hydroxylated $IrO_2(110)$ surface at 1.23 V and 1.53 V vs. RHE. The reaction intermediates of the mechanistic cycle are labeled on the x-axis. Blue and green solid lines indicate intermediates' free energies at 1.23 V and 1.53 V, respectively. Colored arrows indicate the free-energy span governing $G_{max}(U)$, with the respective value displayed.
b) Schematic illustration of the oxide mechanism, as described in Section 3.4, on the partly hydroxylated $IrO_2(110)$ surface. Numbers next to the arrows indicate the step sequence, and each structure represents the corresponding reaction intermediate.

5.5 Binuclear Mechanism

Table S11 compiles the free-energy changes for each elementary step at $U = 0$ V vs. RHE and the activity descriptor $G_{max}(U)$ at different applied electrode potentials under OER conditions. The corresponding free-energy diagram is depicted in Figure S12a. In the potential range of 1.23 V to 1.53 V vs. RHE, the descriptor $G_{max}(U)$ is governed by the free-energy span of the *OH intermediate to the product O_2.

Table S11. Energetic evaluation of the binuclear mechanism on the partly hydroxylated $IrO_2(110)$ surface (cf. Figure 1c in the main text) by the framework of the descriptor $G_{max}(U)$. The table indicates the free-energy changes of each step at $U = 0$ V vs. RHE and $G_{max}(U)$ values at different applied electrode potentials (U).

ΔG_1 [eV]	ΔG_2 [eV]	ΔG_3 [eV]	ΔG_4 [eV]	ΔG_5 [eV]	$G_{max}(U)$ [eV]				
					**1.23 V**	**1.33 V**	**1.43 V**	**1.53 V**	**1.63 V**
-0.10	-0.22	1.75	1.66	1.83	2.78	2.58	2.38	2.18	1.98

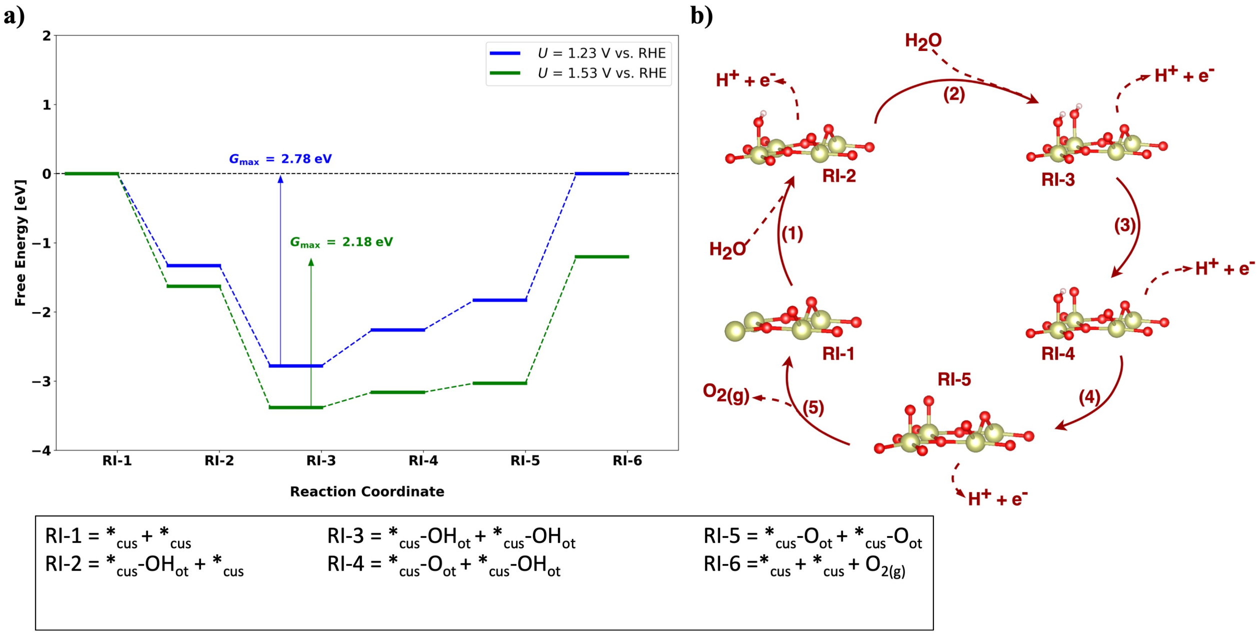

Figure S12. a) Free-energy diagram for the binuclear mechanism on the partly hydroxylated $IrO_2(110)$ surface at 1.23 V and 1.53 V vs. RHE. The reaction intermediates of the mechanistic cycle are labeled on the x-axis. Blue and green solid lines indicate intermediates' free energies at 1.23 V and 1.53 V, respectively. Colored arrows indicate the free-energy span governing $G_{max}(U)$, with the respective value displayed.
b) Schematic illustration of the binuclear mechanism, as described in Section 3.5, on the partly hydroxylated $IrO_2(110)$ surface. Numbers next to the arrows indicate the step sequence, and each structure represents the corresponding reaction intermediate.

5.6 Assessment of solvation effects by VASPsol

Table S12 compiles the calculated free-energy changes neglecting or including solvation, their differences, and the difference in the activity descriptor $G_{max}(U)$. While the quantitative impact of solvation on the free-energy changes does not exceed 0.30 eV, the value of the activity descriptor $G_{max}(U)$ is particularly unchanged in the potential range of 1.23 V to 1.43 V vs. RHE.

Table S12. Comparing the energetics of gas-phase DFT calculations and DFT including implicit solvation by means of VASPsol for the elementary steps of the mononuclear OER mechanism on the partly hydroxylated $IrO_2(110)$ surface. Note that $\Delta\Delta G_j$ (j = 1, 2, 3, 4) indicates the free-energy difference of the free-energy changes for the continuum solvation approach and gas-phase DFT. Likewise, $\Delta G_{max}(U)$ denotes the difference in the activity descriptor $G_{max}(U)$ for the continuum solvation approach and gas-phase DFT.

	ΔG_1 [eV]	ΔG_2 [eV]	ΔG_3 [eV]	ΔG_4 [eV]
**Gas-phase DFT**	-0.22	1.75	1.11	2.27
**DFT + VASPsol**	-0.21	1.55	1.40	2.18

$\Delta\Delta G_j = \Delta G_{j;\ VASPsol} - \Delta G_{j;\ gas}$ [eV]				$\Delta G_{max}(U) = (G_{max}(U))_{VASPsol} - (G_{max}(U))_{gas}$ [eV]				
$\Delta\Delta G_1$	$\Delta\Delta G_2$	$\Delta\Delta G_3$	$\Delta\Delta G_4$	**1.23 V**	**1.33 V**	**1.43 V**	**1.53 V**	**1.63 V**
0.01	-0.20	0.29	-0.09	-0.01	-0.01	-0.01	-0.10	-0.10

6 OER over the fully oxygen-covered $IrO_2(110)$ surface

6.1 Mononuclear mechanism

Table S13 compiles the free-energy changes for each elementary step at $U = 0$ V vs. RHE and the activity descriptor $G_{max}(U)$ at different applied electrode potentials under OER conditions. The corresponding free-energy diagram is depicted in Figure S13a, with a visual representation of the elementary steps in Figure S13b. While the activity descriptor $G_{max}(U)$ is governed by the span $*_{cus}$-OH_{ot} → $*_{cus}$-O_{ot} → $*_{cus}$-OOH_{ot} → $*_{cus}$ + O_2 at $U = 1.23$ V vs. RHE, the limiting free-energy span switches to $*_{cus}$-OOH_{ot} → $*_{cus}$ + O_2 for larger applied overpotentials ($U = 1.53$ V vs. RHE).

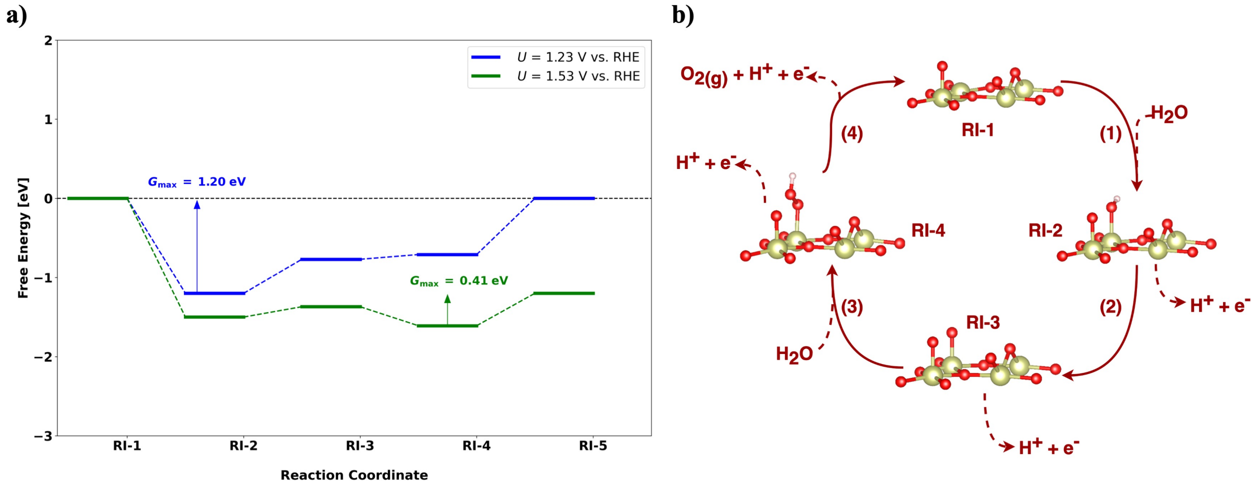

RI-1 = $*_{cus}$	RI-3 = $*_{cus}$-O_{ot}	RI-5 = $*_{cus}$ + $O_{2(g)}$
RI-2 = $*_{cus}$-OH_{ot}	RI-4 = $*_{cus}$-OOH_{ot}	

Figure S13. a) Free-energy diagram for the mononuclear mechanism on the fully oxygen-covered $IrO_2(110)$ surface at 1.23 V and 1.53 V vs. RHE. The reaction intermediates of the mechanistic cycle are labeled on the x-axis. Blue and green solid lines indicate intermediates' free energies at 1.23 V and 1.53 V, respectively. Colored arrows indicate the free-energy span governing $G_{max}(U)$, with the respective value displayed.
b) Schematic illustration of the mononuclear mechanism, as described in Section 3.1, on the fully oxygen-covered $IrO_2(110)$ surface. Numbers next to the arrows indicate the step sequence, and each structure represents the corresponding reaction intermediate.

Table S13. Energetic evaluation of the mononuclear mechanism on the fully oxygen-covered $IrO_2(110)$ surface (cf. Figure 1d in the main text) by the framework of the descriptor $G_{max}(U)$. The table indicates the free-energy changes of each step at $U = 0$ V vs. RHE and $G_{max}(U)$ values at different applied electrode potentials (U).

**ΔG_1** [eV]	**ΔG_2** [eV]	**ΔG_3** [eV]	**ΔG_4** [eV]	**$G_{max}(U)$** [eV]				
				**1.23**	**1.33**	**1.43**	**1.53**	**1.63**
0.03	1.66	1.29	1.93	1.20	0.90	0.60	0.41	0.31

6.2 Bifunctional I mechanism

Table S14 compiles the free-energy changes for each elementary step at $U = 0$ V vs. RHE and the activity descriptor $G_{max}(U)$ at different applied electrode potentials under OER conditions. The corresponding free-energy diagram is depicted in Figure S14a. Similar to the mononuclear mechanism, the activity descriptor $G_{max}(U)$ is governed by the span $*_{cus}$-OH_{ot} + O_{br} → $*_{cus}$-O_{ot} + O_{br} → $*_{cus}$-OO_{ot} + OH_{br} → $*_{cus}$ + O_{br} + O_2 at $U = 1.23$ V vs. RHE, whereas the limiting free-energy span switches to $*_{cus}$-OO_{ot} + OH_{br} → $*_{cus}$ + O_{br} + O_2 for larger applied overpotentials ($U = 1.53$ V vs. RHE).

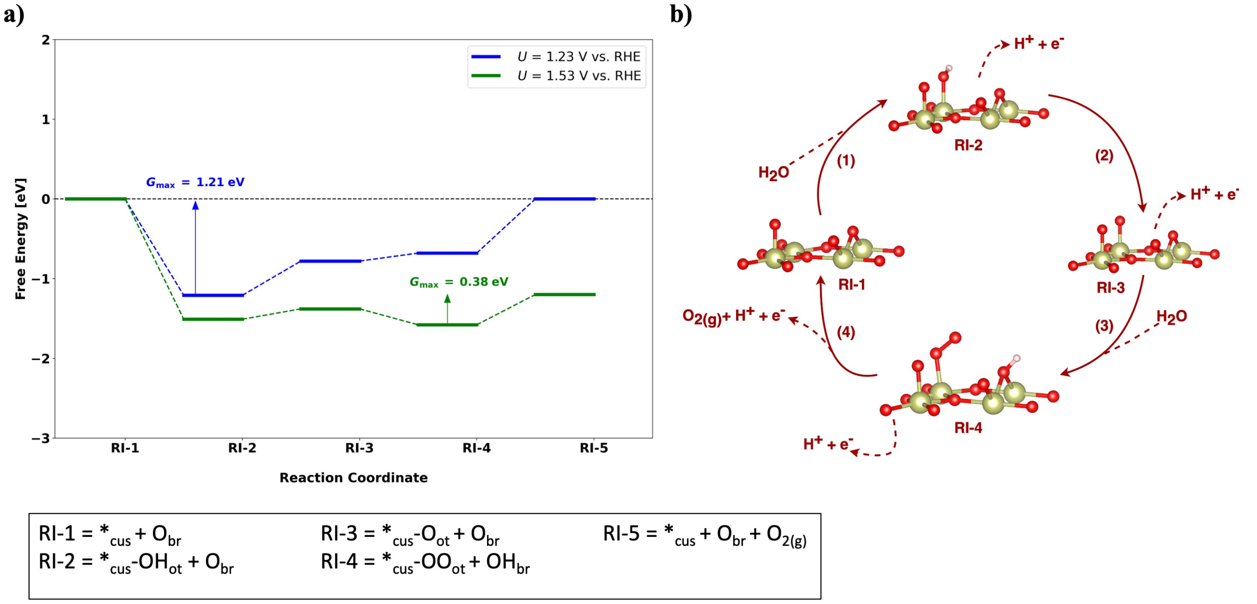

Figure S14. a) Free-energy diagram for the bifunctional I mechanism on the fully oxygen-covered $IrO_2(110)$ surface at 1.23 V and 1.53 V vs. RHE. The reaction intermediates of the mechanistic cycle are labeled on the x-axis. Blue and green solid lines indicate intermediates' free energies at 1.23 V and 1.53 V, respectively. Colored arrows indicate the free-energy span governing $G_{max}(U)$, with the respective value displayed.
b) Schematic illustration of the bifunctional I mechanism, as described in Section 3.2, on the fully oxygen-covered $IrO_2(110)$ surface. Numbers next to the arrows indicate the step sequence, and each structure represents the corresponding reaction intermediate.

Table S14. Energetic evaluation of the bifunctional I mechanism on the fully oxygen-covered $IrO_2(110)$ surface (cf. Figure 1d in the main text) by the framework of the descriptor $G_{max}(U)$. The table indicates the free-energy changes of each step at $U = 0$ V vs. RHE and $G_{max}(U)$ values at different applied electrode potentials (U).

ΔG_1 [eV]	ΔG_2 [eV]	ΔG_3 [eV]	ΔG_4 [eV]	$G_{max}(U)$ [eV]				
				**1.23**	**1.33**	**1.43**	**1.53**	**1.63**
0.02	1.66	1.33	1.91	1.21	0.91	0.61	0.38	0.28

6.3 Bifunctional II mechanism

Table S15 compiles the free-energy changes for each elementary step at $U = 0$ V vs. RHE and the activity descriptor $G_{max}(U)$ at different applied electrode potentials under OER conditions. The corresponding free-energy diagram is depicted in Figure S15a. Similar to the other mechanisms, the activity descriptor $G_{max}(U)$ is governed by the span $*_{cus}\text{-}OH_{ot} + O_{br} \rightarrow *_{cus}\text{-}O_{ot} + O_{br} \rightarrow *_{cus}\text{-}OOH_{ot} + OH_{br} \rightarrow *_{cus}\text{-}OOH_{ot} + O_{br} \rightarrow *_{cus} + O_{br} + O_2$ at $U = 1.23$ V vs. RHE, whereas the limiting free-energy span switches to $*_{cus}\text{-}OOH_{ot} + O_{br} \rightarrow *_{cus} + O_{br} + O_2$ for more anodic conditions ($U = 1.53$ V vs. RHE).

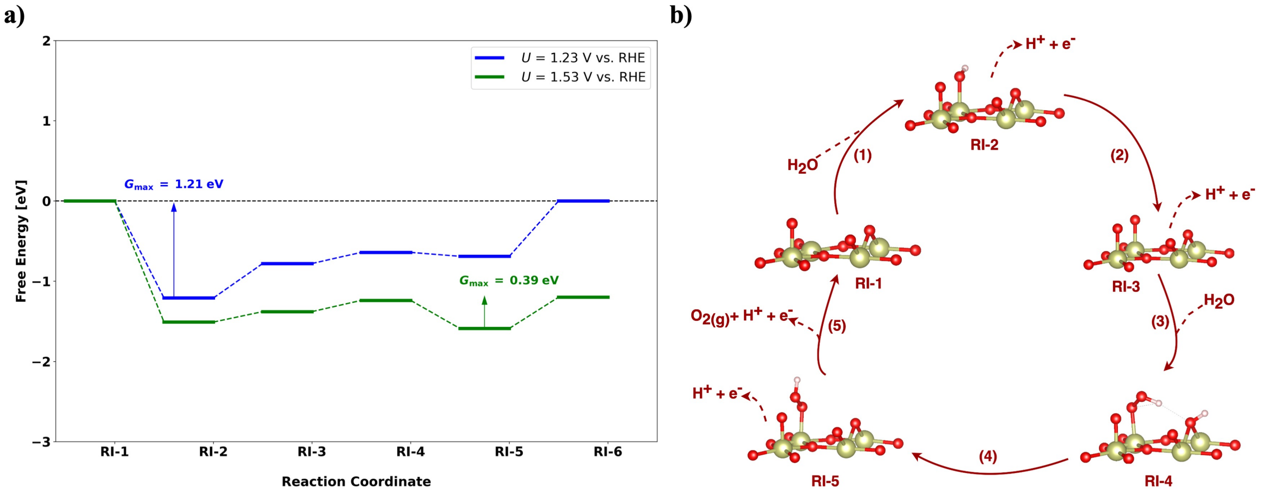

RI-1 = $*_{cus} + O_{br}$ RI-3 = $*_{cus}\text{-}O_{ot} + O_{br}$ RI-5 = $*_{cus}\text{-}OOH_{ot} + O_{br}$

RI-2 = $*_{cus}\text{-}OH_{ot} + O_{br}$ RI-4 = $*_{cus}\text{-}OOH_{ot} + OH_{br}$ RI-6 = $*_{cus} + O_{br} + O_{2(g)}$

Figure S15. a) Free-energy diagram for the bifunctional II mechanism on the fully oxygen-covered $IrO_2(110)$ surface at 1.23 V and 1.53 V vs. RHE. The reaction intermediates of the mechanistic cycle are labeled on the x-axis. Blue and green solid lines indicate intermediates' free energies at 1.23 V and 1.53 V, respectively. Colored arrows indicate the free-energy span governing $G_{max}(U)$, with the respective value displayed.
b) Schematic illustration of the bifunctional II mechanism, as described in Section 3.3, on the fully oxygen-covered $IrO_2(110)$ surface. Numbers next to the arrows indicate the step sequence, and each structure represents the corresponding reaction intermediate.

Table S15. Energetic evaluation of the bifunctional II mechanism on the fully oxygen-covered $IrO_2(110)$ surface (cf. Figure 1d in the main text) by the framework of the descriptor $G_{max}(U)$. The table indicates the free-energy changes of each step at $U = 0$ V vs. RHE and $G_{max}(U)$ values at different applied electrode potentials (U).

ΔG_1 [eV]	ΔG_2 [eV]	ΔG_3 [eV]	ΔG_4 [eV]	ΔG_5 [eV]	$G_{max}(U)$ [eV]				
					**1.23**	**1.33**	**1.43**	**1.53**	**1.63**
0.02	1.66	0.14	1.18	1.90	1.21	0.91	0.61	0.39	0.27

6.4 Oxide mechanism

Table S16 compiles the free-energy changes for each elementary step at $U = 0$ V vs. RHE and the activity descriptor $G_{max}(U)$ at different applied electrode potentials under OER conditions. The corresponding free-energy diagram is depicted in Figure S16a. In contrast to the other mechanisms, the activity descriptor $G_{max}(U)$ is governed by the formation rather than the decomposition of the *OOH intermediate from the *O-covered surface. We note that the obtained results are identical to the partly hydroxylated surface in section 5.4 due to the following reason: the partly hydroxylated phase first needs to be transformed into a fully oxygen-covered surface, which refers to the reference structure of this section.

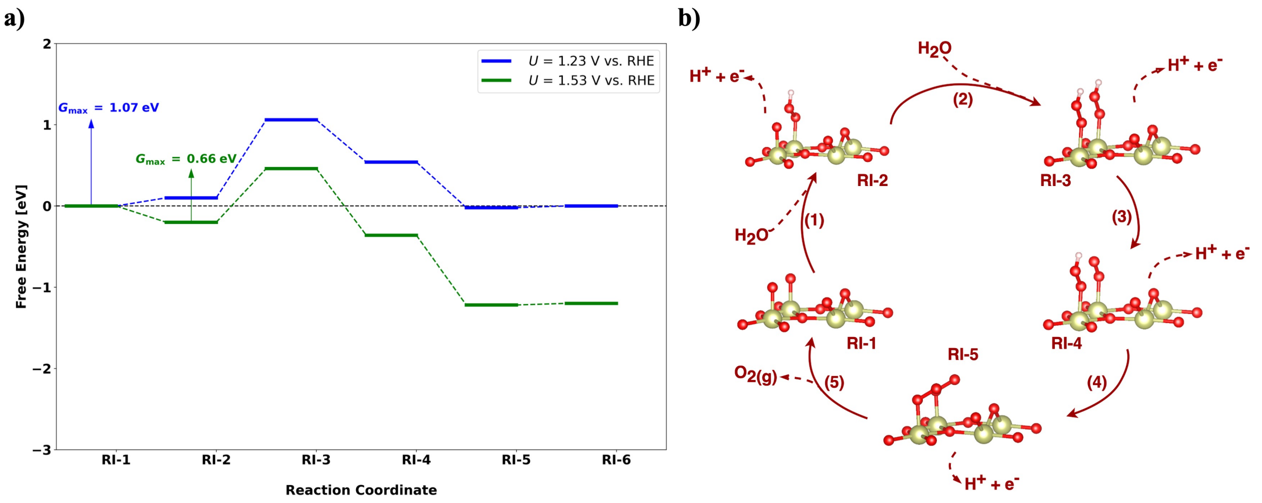

RI-1 = $*_{cus}$-O_{ot} + $*_{cus}$-O_{ot}
RI-2 = $*_{cus}$-OOH_{ot} + $*_{cus}$-O_{ot}
RI-3 = $*_{cus}$-OOH_{ot} + $*_{cus}$-OOH_{ot}
RI-4 = $*_{cus}$-OOH_{ot} + $*_{cus}$-OO_{ot}
RI-5 = $*_{cus}$-OO_{ot} + $*_{cus}$-OO_{ot}
RI-6 = $*_{cus}$-O_{ot} + $*_{cus}$-O_{ot} + $O_{2(g)}$

Figure S16. a) Free-energy diagram for the oxide mechanism on the fully oxygen-covered IrO_2(110) surface at 1.23 V and 1.53 V vs. RHE. The reaction intermediates of the mechanistic cycle are labeled on the x-axis. Blue and green solid lines indicate intermediates' free energies at 1.23 V and 1.53 V, respectively. Colored arrows indicate the free-energy span governing $G_{max}(U)$, with the respective value displayed.
b) Schematic illustration of the oxide mechanism, as described in Section 3.4, on the fully oxygen-covered IrO_2(110) surface. Numbers next to the arrows indicate the step sequence, and each structure represents the corresponding reaction intermediate.

Table S16. Energetic evaluation of the oxide mechanism on the fully oxygen-covered IrO_2(110) surface (cf. Figure 1d in the main text) by the framework of the descriptor $G_{max}(U)$. The table indicates the free-energy changes of each step at $U = 0$ V vs. RHE and $G_{max}(U)$ values at different applied electrode potentials (U).

ΔG_1 [eV]	ΔG_2 [eV]	ΔG_3 [eV]	ΔG_4 [eV]	ΔG_5 [eV]	$G_{max}(U)$ [eV]				
					**1.23 V**	**1.33 V**	**1.43 V**	**1.53 V**	**1.63 V**
1.33	2.19	0.71	0.67	0.01	1.07	0.87	0.76	0.66	0.56

6.5 Binuclear mechanism

Table S17 compiles the free-energy changes for each elementary step at $U = 0$ V vs. RHE and the activity descriptor $G_{max}(U)$ at different applied electrode potentials under OER conditions. The corresponding free-energy diagram is depicted in Figure S17a. In the potential range of 1.23 V to 1.53 V vs. RHE, the descriptor $G_{max}(U)$ is governed by the free-energy span of the *OH intermediate to the product O_2.

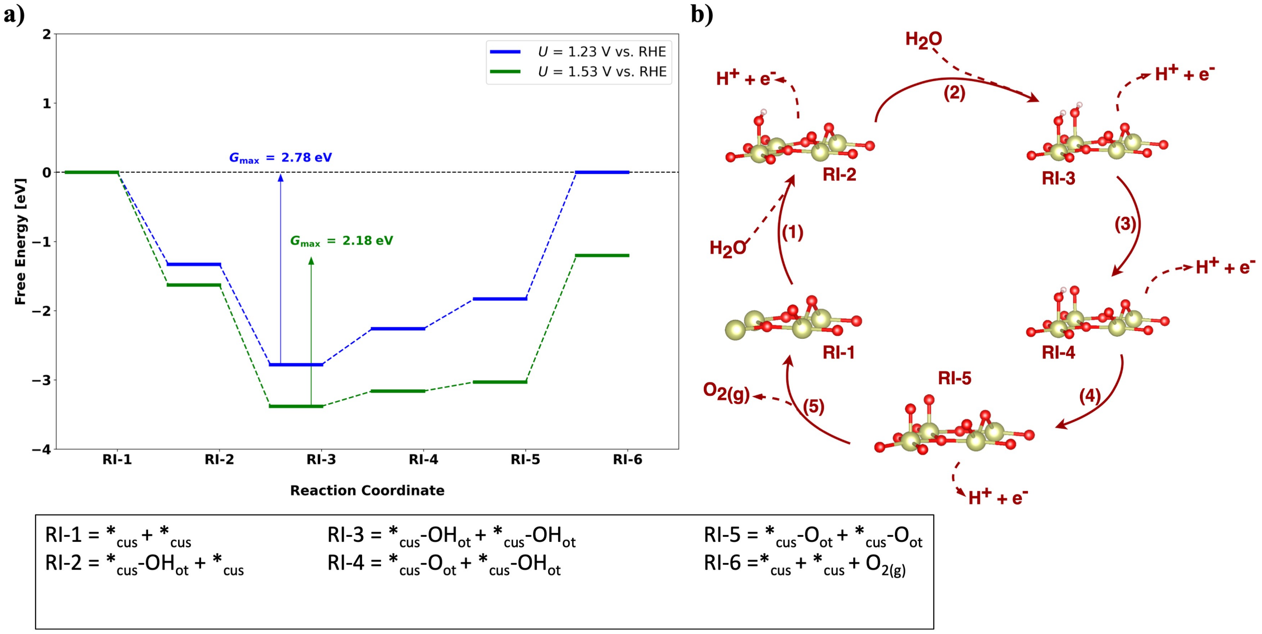

Figure S17. a) Free-energy diagram for the binuclear mechanism on the fully oxygen-covered $IrO_2(110)$ surface at 1.23 V and 1.53 V vs. RHE. The reaction intermediates of the mechanistic cycle are labeled on the x-axis. Blue and green solid lines indicate intermediates' free energies at 1.23 V and 1.53 V, respectively. Colored arrows indicate the free-energy span governing $G_{max}(U)$, with the respective value displayed.
b) Schematic illustration of the binuclear mechanism, as described in Section 3.5, on the fully oxygen-covered $IrO_2(110)$ surface. Numbers next to the arrows indicate the step sequence, and each structure represents the corresponding reaction intermediate.

Table S17. Energetic evaluation of the binuclear mechanism on the fully oxygen-covered $IrO_2(110)$ surface (cf. Figure 1d in the main text) by the framework of the descriptor $G_{max}(U)$. The table indicates the free-energy changes of each step at $U = 0$ V vs. RHE and $G_{max}(U)$ values at different applied electrode potentials (U).

ΔG_1 [eV]	ΔG_2 [eV]	ΔG_3 [eV]	ΔG_4 [eV]	ΔG_5 [eV]	$G_{max}(U)$ [eV]				
					**1.23 V**	**1.33 V**	**1.43 V**	**1.53 V**	**1.63 V**
-0.10	-0.22	1.75	1.66	1.83	2.78	2.58	2.38	2.18	1.98

6.6 Assessment of solvation effects by VASPsol

Table S18 compiles the calculated free-energy changes neglecting or including solvation, their differences, and the difference in the activity descriptor $G_{max}(U)$. While the quantitative impact of solvation on the free-energy changes does not exceed 0.25 eV, the value of the activity descriptor $G_{max}(U)$ is unchanged for large overpotentials ($U > 1.53$ V vs. RHE).

Table S18. Comparing the energetics of gas-phase DFT calculations and DFT including implicit solvation by means of VASPsol for the elementary steps of the mononuclear OER mechanism on the fully oxygen-covered $IrO_2(110)$ surface. Note that $\Delta\Delta G_j$ (j = 1, 2, 3, 4) indicates the free-energy difference of the free-energy changes for the continuum solvation approach and gas-phase DFT. Likewise, $\Delta G_{max}(U)$ denotes the difference in the activity descriptor $G_{max}(U)$ for the continuum solvation approach and gas-phase DFT.

	ΔG_1 [eV]	ΔG_2 [eV]	ΔG_3 [eV]	ΔG_4 [eV]
**Gas-phase DFT**	0.03	1.66	1.29	1.93
**DFT + VASPsol**	-0.09	1.57	1.54	1.90

$\Delta\Delta G_j = \Delta G_{j;\,VASPsol} - \Delta G_{j;\,gas}$ [eV]				$\Delta G_{max}(U) = (G_{max}(U))_{VASPsol} - (G_{max}(U))_{gas}$ [eV]				
$\Delta\Delta G_1$	$\Delta\Delta G_2$	$\Delta\Delta G_3$	$\Delta\Delta G_4$	**1.23 V**	**1.33 V**	**1.43 V**	**1.53 V**	**1.63 V**
-0.12	-0.09	0.25	-0.03	0.12	0.12	0.12	0.01	-0.04

6.7 Assessment of constant potential DFT

Catalytic processes at electrified solid/ liquid interfaces take place at constant potential (grand canonical ensemble) rather than at constant charge (canonical ensemble) conditions, though the latter refers to the application of the CHE approach. To qualitatively assess the impact of the different ensembles on activity predictions, we adopt the mononuclear description on the fully oxygen-covered $IrO_2(110)$ surface and compare the constant potential and constant charge descriptions at U = 1.43 V vs. RHE. Note that the application of the grand canonical approach also contains the inclusion of implicit solvation by VASPsol package whereas the canonical description relies on gas-phase DFT. Table S19 compiles the calculated free-energy changes in the canonical and grand canonical ensembles at U = 1.43 V vs. RHE as well as the activity descriptor $G_{max}(U = 1.43\ V)$. Given that the difference in $G_{max}(U = 1.43\ V)$ is only 0.02 eV, we conclude that it is sufficient to apply the constant charge formalism in the realm of the CHE approach to study the elementary steps of the OER over $IrO_2(110)$.

Table S19. Comparing the energetics of gas-phase DFT calculations and grand canonical DFT (GC-DFT) including implicit solvation by means of VASPsol for the elementary steps of the mononuclear OER mechanism over the fully oxygen-covered $IrO_2(110)$ surface at U = 1.43 V vs. RHE.

	ΔG_1 [eV]	ΔG_2 [eV]	ΔG_3 [eV]	ΔG_4 [eV]	$G_{max}(U)$ [eV]
	**U = 1.43 V**				
**Gas-phase DFT**	-1.40	0.23	-0.14	0.50	0.60
**GC-DFT**	-1.06	0.33	-0.69	0.62	0.62

7 OER over the partly OOH-covered $IrO_2(110)$ surface

7.1 Mononuclear mechanism

Table S20 compiles the free-energy changes for each elementary step at $U = 0$ V vs. RHE and the activity descriptor $G_{max}(U)$ at different applied electrode potentials under OER conditions. The corresponding free-energy diagram is depicted in Figure S18a, with a visual representation of the elementary steps in Figure S18b. The activity descriptor $G_{max}(U)$ is governed by the span $*_{cus}$-OH_{ot} → $*_{cus}$-O_{ot} → $*_{cus}$-OOH_{ot} in the potential range of $U = 1.23$ V to 1.53 V vs. RHE.

Table S20. Energetic evaluation of the mononuclear mechanism on the partly OOH-covered $IrO_2(110)$ surface (cf. Figure 1e in the main text) by the framework of the descriptor $G_{max}(U)$. The table indicates the free-energy changes of each step at $U = 0$ V vs. RHE and $G_{max}(U)$ values at different applied electrode potentials (U).

ΔG_1 [eV]	ΔG_2 [eV]	ΔG_3 [eV]	ΔG_4 [eV]	$G_{max}(U)$ [eV]				
				**1.23 V**	**1.33 V**	**1.43 V**	**1.53 V**	**1.63 V**
-0.23	1.88	2.19	1.07	1.61	1.41	1.21	1.01	0.81

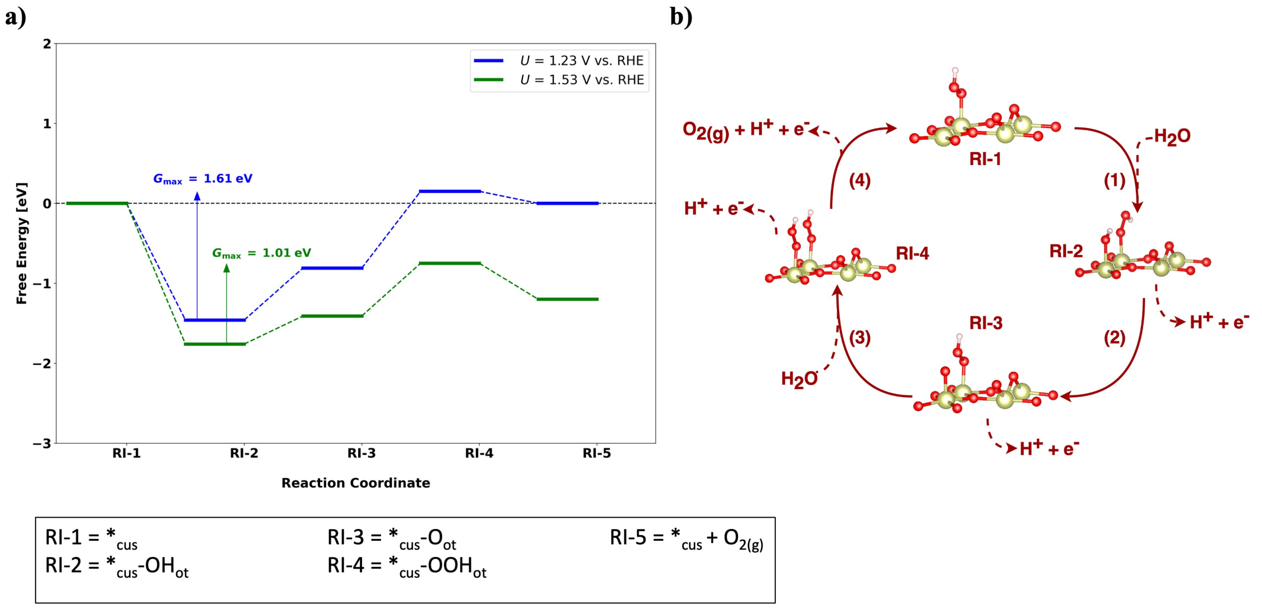

Figure S18. a) Free-energy diagram for the mononuclear mechanism on the partly OOH-covered $IrO_2(110)$ surface at 1.23 V and 1.53 V vs. RHE. The reaction intermediates of the mechanistic cycle are labeled on the x-axis. Blue and green solid lines indicate intermediates' free energies at 1.23 V and 1.53 V, respectively. Colored arrows indicate the free-energy span governing $G_{max}(U)$, with the respective value displayed.
b) Schematic illustration of the mononuclear mechanism, as described in Section 3.1, on the partly OOH-covered $IrO_2(110)$ surface. Numbers next to the arrows indicate the step sequence, and each structure represents the corresponding reaction intermediate.

7.2 Bifunctional I mechanism

Table S21 compiles the free-energy changes for each elementary step at $U = 0$ V vs. RHE and the activity descriptor $G_{max}(U)$ at different applied electrode potentials under OER conditions. The

corresponding free-energy diagram is depicted in Figure S19a. Similar to the mononuclear mechanism, the activity descriptor $G_{max}(U)$ is governed by the span $*_{cus}$-OH_{ot} + O_{br}→ $*_{cus}$-O_{ot} + O_{br} → $*_{cus}$-OO_{ot} + OH_{br} → $*_{cus}$ + O_{br} + O_2 in the potential range of U = 1.23 V to 1.53 V vs. RHE.

Table S21. Energetic evaluation of the bifunctional I mechanism on the partly OOH-covered $IrO_2(110)$ surface (cf. Figure 1e in the main text) by the framework of the descriptor $G_{max}(U)$. The table indicates the free-energy changes of each step at U = 0 V vs. RHE and $G_{max}(U)$ values at different applied electrode potentials (U).

**ΔG_1** [eV]	**ΔG_2** [eV]	**ΔG_3** [eV]	**ΔG_4** [eV]	**$G_{max}(U)$** [eV]				
				**1.23 V**	**1.33 V**	**1.43 V**	**1.53 V**	**1.63 V**
-0.23	1.88	1.35	1.91	1.46	1.16	0.86	0.56	0.28

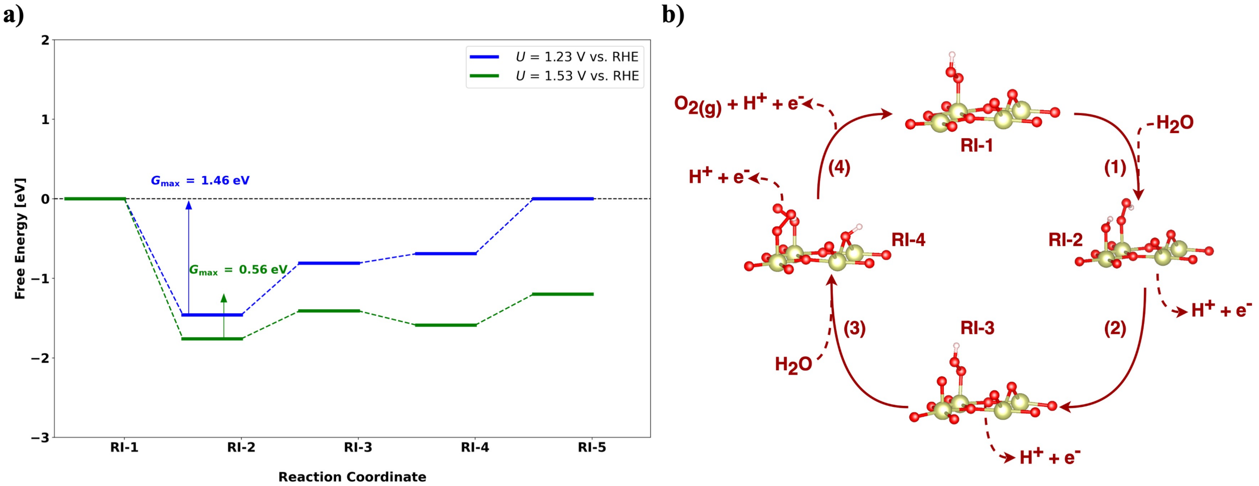

RI-1 = $*_{cus}$ + O_{br}
RI-2 = $*_{cus}$-OH_{ot} + O_{br}
RI-3 = $*_{cus}$-O_{ot} + O_{br}
RI-4 = $*_{cus}$-OO_{ot} + OH_{br}
RI-5 = $*_{cus}$ + O_{br} + $O_{2(g)}$

Figure S19. a) Free-energy diagram for the bifunctional I mechanism on the partly OOH-covered $IrO_2(110)$ surface at 1.23 V and 1.53 V vs. RHE. The reaction intermediates of the mechanistic cycle are labeled on the x-axis. Blue and green solid lines indicate intermediates' free energies at 1.23 V and 1.53 V, respectively. Colored arrows indicate the free-energy span governing $G_{max}(U)$, with the respective value displayed.
b) Schematic illustration of the bifunctional I mechanism, as described in Section 3.2, on the partly OOH-covered $IrO_2(110)$ surface. Numbers next to the arrows indicate the step sequence, and each structure represents the corresponding reaction intermediate.

7.3 Bifunctional II mechanism

Table S22 compiles the free-energy changes for each elementary step at U = 0 V vs. RHE and the activity descriptor $G_{max}(U)$ at different applied electrode potentials under OER conditions. The corresponding free-energy diagram is depicted in Figure S20a. The activity descriptor $G_{max}(U)$ is governed by the span $*_{cus}$-OH_{ot} + O_{br} → $*_{cus}$-O_{ot} + O_{br} → $*_{cus}$-OOH_{ot} + OH_{br} → $*_{cus}$-OOH_{ot} + O_{br} in the potential range of U = 1.23 V to 1.53 V vs. RHE.

Table S22. Energetic evaluation of the bifunctional II mechanism on the partly OOH-covered $IrO_2(110)$ surface (cf. Figure 1e in the main text) by the framework of the descriptor $G_{max}(U)$. The table indicates the free-energy changes of each step at $U = 0$ V vs. RHE and $G_{max}(U)$ values at different applied electrode potentials (U).

ΔG_1 [eV]	ΔG_2 [eV]	ΔG_3 [eV]	ΔG_4 [eV]	ΔG_5 [eV]	$G_{max}(U)$ [eV]				
					**1.23 V**	**1.33 V**	**1.43 V**	**1.53 V**	**1.63 V**
-0.23	1.88	0.33	1.86	1.07	1.61	1.41	1.21	1.01	0.81

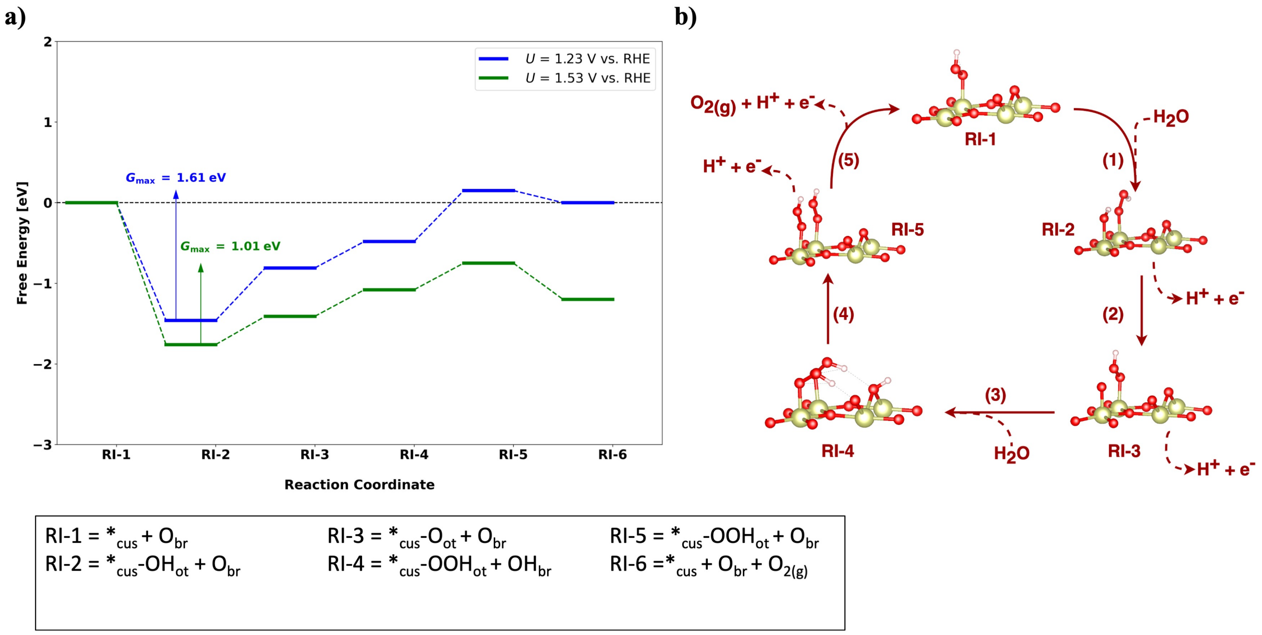

Figure S20. a) Free-energy diagram for the bifunctional II mechanism on the partly OOH-covered $IrO_2(110)$ surface at 1.23 V and 1.53 V vs. RHE. The reaction intermediates of the mechanistic cycle are labeled on the x-axis. Blue and green solid lines indicate intermediates' free energies at 1.23 V and 1.53 V, respectively. Colored arrows indicate the free-energy span governing $G_{max}(U)$, with the respective value displayed.
b) Schematic illustration of the bifunctional II mechanism, as described in Section 3.3, on the partly OOH-covered $IrO_2(110)$ surface. Numbers next to the arrows indicate the step sequence, and each structure represents the corresponding reaction intermediate.

7.4 Oxide mechanism

Table S23 compiles the free-energy changes for each elementary step at $U = 0$ V vs. RHE and the activity descriptor $G_{max}(U)$ at different applied electrode potentials under OER conditions. The corresponding free-energy diagram is depicted in Figure S21a. In contrast to the other mechanisms, the activity descriptor $G_{max}(U)$ is governed by the formation rather than the decomposition of the *OOH intermediate from the *O-covered surface. We note that the obtained results are identical to the partly hydroxylated and fully oxygen-covered surfaces in sections 5.4 and 6.4, respectively.

Table S23. Energetic evaluation of the oxide mechanism on the partly OOH-covered $IrO_2(110)$ surface (cf. Figure 1e in the main text) by the framework of the descriptor $G_{max}(U)$. The table indicates the free-energy changes of each step at $U = 0$ V vs. RHE and $G_{max}(U)$ values at different applied electrode potentials (U).

ΔG_1 [eV]	ΔG_2 [eV]	ΔG_3 [eV]	ΔG_4 [eV]	ΔG_5 [eV]	$G_{max}(U)$ [eV]				
					**1.23 V**	**1.33 V**	**1.43 V**	**1.53 V**	**1.63 V**
1.33	2.19	0.71	0.67	0.01	1.07	0.87	0.76	0.66	0.56

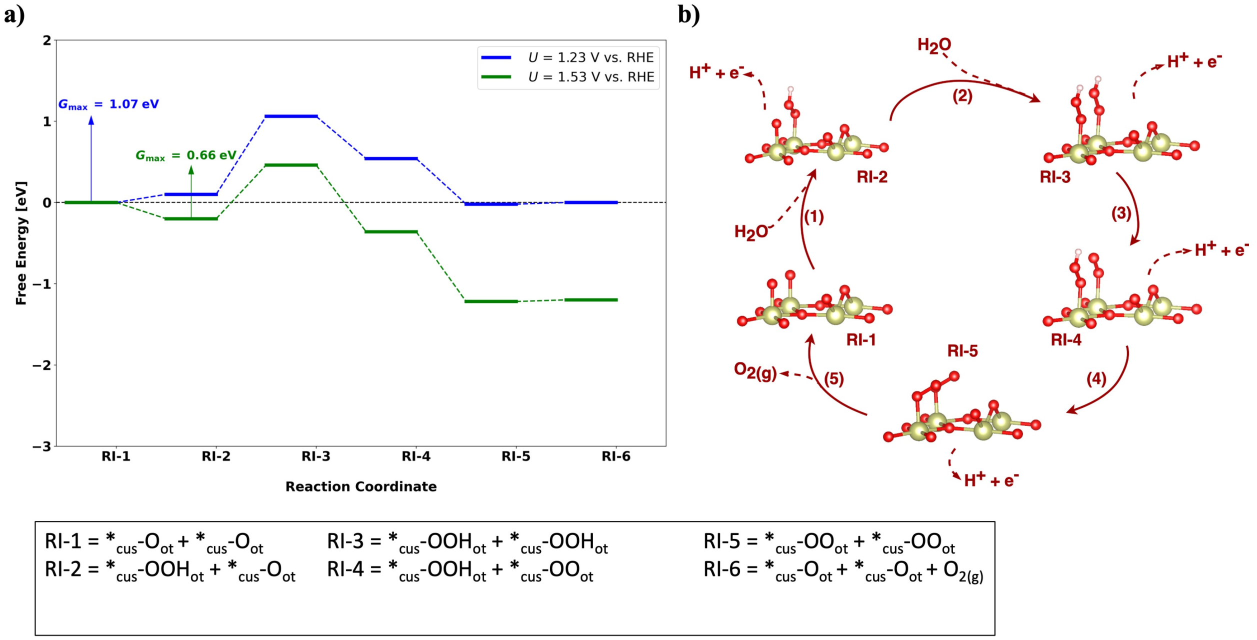

Figure S21. a) Free-energy diagram for the oxide mechanism on the partly OOH-covered $IrO_2(110)$ surface at 1.23 V and 1.53 V vs. RHE. The reaction intermediates of the mechanistic cycle are labeled on the x-axis. Blue and green solid lines indicate intermediates' free energies at 1.23 V and 1.53 V, respectively. Colored arrows indicate the free-energy span governing $G_{max}(U)$, with the respective value displayed.
b) Schematic illustration of the oxide mechanism, as described in Section 3.4, on the partly OOH-covered $IrO_2(110)$ surface. Numbers next to the arrows indicate the step sequence, and each structure represents the corresponding reaction intermediate.

7.5 Binuclear mechanism

The binuclear mechanism consists of the chemical recombination of two adjacent *O adsorbates. Due to the presence of the $*_{cus}$-OOH_{ot} intermediate on the neighboring cus site, we conclude that the binuclear mechanism cannot proceed on the partly OOH-covered surface due to the lack of surface oxygen, $*_{cus}$-O_{ot}, on the cus sites.

7.6 Assessment of solvation effects by VASPsol

Table S24 compiles the calculated free-energy changes neglecting or including solvation, their differences, and the difference in the activity descriptor $G_{max}(U)$. While the quantitative impact of solvation on the free-energy changes is less than 0.20 eV, we observe that there is a constant

difference of 0.15 eV for the values of the activity descriptor $G_{max}(U)$ for the two different approaches.

Table S24. Comparing the energetics of gas-phase DFT calculations and DFT including implicit solvation by means of VASPsol for the elementary steps of the mononuclear OER mechanism on the partly OOH-covered $IrO_2(110)$ surface. Note that $\Delta\Delta G_j$ (j = 1, 2, 3, 4) indicates the free-energy difference of the free-energy changes for the continuum solvation approach and gas-phase DFT. Likewise, $\Delta G_{max}(U)$ denotes the difference in the activity descriptor $G_{max}(U)$ for the continuum solvation approach and gas-phase DFT.

	ΔG_1 [eV]	ΔG_2 [eV]	ΔG_3 [eV]	ΔG_4 [eV]
**Gas-phase DFT**	-0.23	1.88	2.19	1.07
**DFT + VASPsol**	-0.23	1.71	2.22	1.22

$\Delta\Delta G_j = \Delta G_{j; VASPsol} - \Delta G_{j; gas}$ [eV]				$\Delta G_{max}(U) = (G_{max}(U))_{VASPsol} - (G_{max}(U))_{gas}$ [eV]				
$\Delta\Delta G_1$	$\Delta\Delta G_2$	$\Delta\Delta G_3$	$\Delta\Delta G_4$	**1.23 V**	**1.33 V**	**1.43 V**	**1.53 V**	**1.63 V**
0	-0.17	0.03	0.15	-0.15	-0.15	-0.15	-0.15	-0.15

8 Elementary steps of Walden pathways

All the reaction mechanisms discussed so far rely on the notion that the unoccupied metal site, $*_{cus}$, is formed after product formation. However, it is also possible that product desorption and reactant adsorption proceed simultaneously, giving rise to so-called Walden pathways.[35] In these mechanisms, the metal site is always occupied with an adsorbate and does not become vacant in the course of the catalysis.

The term Walden inversion is coined from the field of organic chemistry where it refers to the concurrent removal of a leaving group and adsorption of the reactant with an inversion of the stereochemistry.[36] Interestingly, this scenario can also be transferred to surface reactions in heterogeneous catalysis, as recently highlighted by two different groups.[35,37]

In the following, we provide reaction equations for the elementary steps of the mononuclear-Walden and bifunctional-Walden mechanisms. Please note that we did not consider the oxide-Walden and binuclear-Walden pathways in our study due to the following reason: the oxide- and binuclear-Walden mechanisms require two adjacent active sites to form the product O_2 by a chemical recombination of two neighboring oxygen species. To account for the notion of the Walden inversion in that the metal site is always capped by an adsorbate, it would require the concurrent adsorption of two water molecules on the adjacent active sites during the desorption of the product O_2. We conclude that such a scenario is unlikely due to steric reasons, considering that about 50 % of the surrounding of the active $*_{cus}$ site is unavailable due to the bulk structure

underneath. To this end, we purport that only for a single-site mechanism with one main active site such as the mononuclear and bifunctional descriptions, a Walden inversion mechanism can take place for heterogeneous electrocatalysts.

8.1 Mononuclear-Walden mechanism

$$*_{cus}\text{-}OH_{ot} \rightarrow *_{cus}\text{-}O_{ot} + H^{+} + e^{-} \quad (40)$$

$$*_{cus}\text{-}O_{ot} + H_2O \rightarrow *_{cus}\text{-}OOH_{ot} + H^{+} + e^{-} \quad (41)$$

$$*_{cus}\text{-}OOH_{ot} \rightarrow *_{cus}\text{-}OO_{ot} + H^{+} + e^{-} \quad (42)$$

$$*_{cus}\text{-}OO_{ot} + H_2O \rightarrow *_{cus}\text{-}OH_{ot} + O_{2(g)} + H^{+} + e^{-} \quad (43)$$

8.2 Bifunctional-Walden mechanism

$$*_{cus}\text{-}OH_{ot} + O_{br} \rightarrow *_{cus}\text{-}O_{ot} + O_{br} + H^{+} + e^{-} \quad (44)$$

$$*_{cus}\text{-}O_{ot} + O_{br} + H_2O \rightarrow *_{cus}\text{-}OO_{ot} + OH_{br} + H^{+} + e^{-} \quad (45)$$

$$*_{cus}\text{-}OO_{ot} + OH_{br} \rightarrow *_{cus}\text{-}OO_{ot} + O_{br} + H^{+} + e^{-} \quad (46)$$

$$*_{cus}\text{-}OO_{ot} + O_{br} + H_2O \rightarrow *_{cus}\text{-}OH_{ot} + O_{br} + O_{2(g)} + H^{+} + e^{-} \quad (47)$$

9 Walden pathways over the fully hydroxylated $IrO_2(110)$ surface

9.1 Mononuclear-Walden mechanism

The mononuclear-Walden consists of the sequential formation of the *O (step 1), *OOH (step 2), and *OO (step 3) adsorbates, followed by the simultaneous release of O_2 and formation of the *OH adsorbate in the final step (step 4). Table S25 compiles the free-energy changes for each elementary step at U = 0 V vs. RHE and the activity descriptor $G_{max}(U)$ at different applied electrode potentials under OER conditions. The corresponding free-energy diagram is depicted in Figure S22a, with a visual representation of the elementary steps in Figure S22b. While the activity descriptor $G_{max}(U)$ is governed by the span $*_{cus}\text{-}OH_{ot} \rightarrow *_{cus}\text{-}O_{ot} \rightarrow *_{cus}\text{-}OOH_{ot}$ for U = 1.23 V vs. RHE, the limiting span switches to $*_{cus}\text{-}OH_{ot} \rightarrow *_{cus}\text{-}O_{ot}$ at larger applied overpotentials (1.53 V vs. RHE).

Table S25. Energetic evaluation of the mononuclear-Walden mechanism on the fully hydroxylated $IrO_2(110)$ surface (cf. Figure 1b in the main text) by the framework of the descriptor $G_{max}(U)$. The table indicates the free-energy changes of each step at $U = 0$ V vs. RHE and $G_{max}(U)$ values at different applied electrode potentials (U).

**ΔG_1** [eV]	**ΔG_2** [eV]	**ΔG_3** [eV]	**ΔG_4** [eV]	**$G_{max}(U)$** [eV]				
				**1.23 V**	**1.33 V**	**1.43 V**	**1.53 V**	**1.63 V**
1.65	1.44	1.02	0.81	0.63	0.43	0.23	0.12	0.02

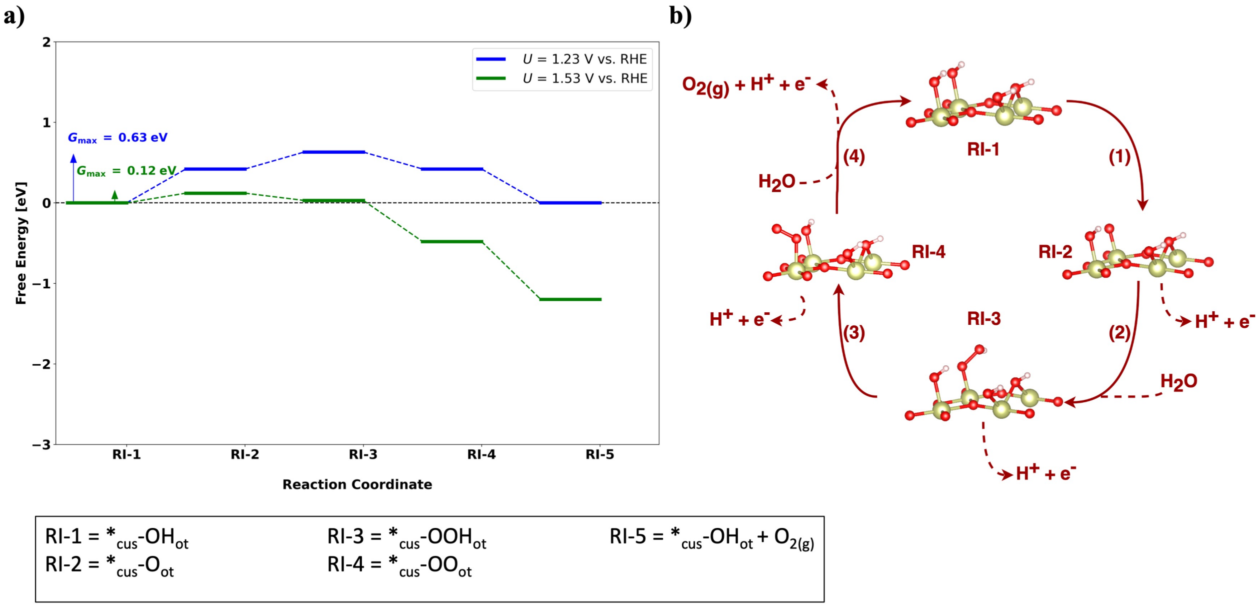

Figure S22. a) Free-energy diagram for the mononuclear-Walden mechanism on the fully hydroxylated $IrO_2(110)$ surface at 1.23 V and 1.53 V vs. RHE. The reaction intermediates of the mechanistic cycle are labeled on the x-axis. Blue and green solid lines indicate intermediates' free energies at 1.23 V and 1.53 V, respectively. Colored arrows indicate the free-energy span governing $G_{max}(U)$, with the respective value displayed.
b) Schematic illustration of the mononuclear-Walden mechanism, as described in Section 8.1, on the fully hydroxylated $IrO_2(110)$ surface. Numbers next to the arrows indicate the step sequence, and each structure represents the corresponding reaction intermediate.

9.2 Bifunctional-Walden mechanism

The bifunctional-Walden differs from the mononuclear-Walden mechanism in that the formation of the *OOH intermediate is circumvented (cf. section 8.2). Table S26 compiles the free-energy changes for each elementary step at $U = 0$ V vs. RHE and the activity descriptor $G_{max}(U)$ at different applied electrode potentials under OER conditions. The corresponding free-energy diagram is depicted in Figure S23a, with a visual representation of the elementary steps in Figure S23b. While the activity descriptor $G_{max}(U)$ is governed by the span $*_{cus}\text{-}OH_{ot} + O_{br} \rightarrow *_{cus}\text{-}O_{ot} + O_{br} \rightarrow *_{cus}\text{-}OO_{ot} + OH_{br}$ for $U = 1.23$ V vs. RHE, the limiting span switches to $*_{cus}\text{-}OH_{ot} + O_{br} \rightarrow *_{cus}\text{-}O_{ot} + O_{br}$ at larger applied overpotentials (1.53 V vs. RHE).

Table S26. Energetic evaluation of the bifunctional-Walden mechanism on the fully hydroxylated $IrO_2(110)$ surface (cf. Figure 1b in the main text) by the framework of the descriptor $G_{max}(U)$. The table indicates the free-energy changes of each step at $U = 0$ V vs. RHE and $G_{max}(U)$ values at different applied electrode potentials (U).

**ΔG_1** [eV]	**ΔG_2** [eV]	**ΔG_3** [eV]	**ΔG_4** [eV]	**$G_{max}(U)$** [eV]				
				**1.23 V**	**1.33 V**	**1.43 V**	**1.53 V**	**1.63 V**
1.66	1.42	1.08	0.77	0.62	0.42	0.23	0.13	0.03

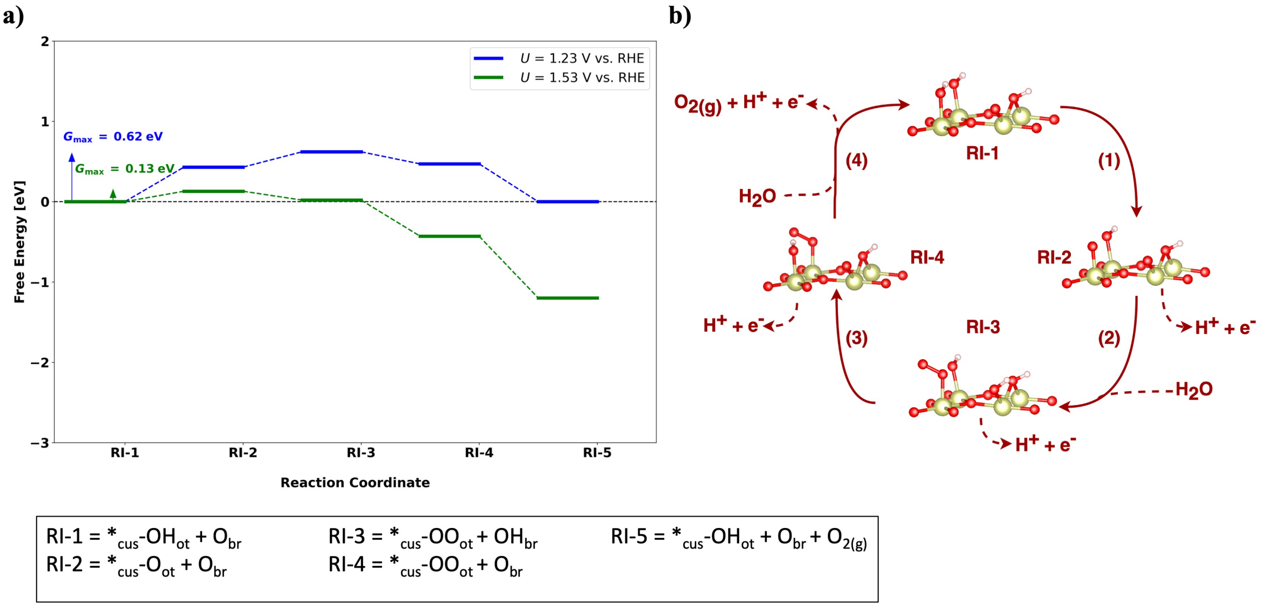

Figure S23. a) Free-energy diagram for the bifunctional-Walden mechanism on the fully hydroxylated $IrO_2(110)$ surface at 1.23 V and 1.53 V vs. RHE. The reaction intermediates of the mechanistic cycle are labeled on the x-axis. Blue and green solid lines indicate intermediates' free energies at 1.23 V and 1.53 V, respectively. Colored arrows indicate the free-energy span governing $G_{max}(U)$, with the respective value displayed.
b) Schematic illustration of the bifunctional-Walden mechanism, as described in Section 8.2, on the fully hydroxylated $IrO_2(110)$ surface. Numbers next to the arrows indicate the step sequence, and each structure represents the corresponding reaction intermediate.

10 Walden pathways over the partly hydroxylated $IrO_2(110)$ surface

10.1 Mononuclear-Walden mechanism

Table S27 compiles the free-energy changes for each elementary step at $U = 0$ V vs. RHE and the activity descriptor $G_{max}(U)$ at different applied electrode potentials under OER conditions. The corresponding free-energy diagram is depicted in Figure S24a, with a visual representation of the elementary steps in Figure S24b. The activity descriptor $G_{max}(U)$ is governed by the span $*_{cus}$-OH_{ot} → $*_{cus}$-O_{ot} in the potential range of $U = 1.23$ V to 1.53 V vs. RHE.

Table S27. Energetic evaluation of the mononuclear-Walden mechanism on the partly hydroxylated $IrO_2(110)$ surface (cf. Figure 1c in the main text) by the framework of the descriptor $G_{max}(U)$. The table indicates the free-energy changes of each step at $U = 0$ V vs. RHE and $G_{max}(U)$ values at different applied electrode potentials (U).

ΔG_1 [eV]	ΔG_2 [eV]	ΔG_3 [eV]	ΔG_4 [eV]	$G_{max}(U)$ [eV]				
				**1.23 V**	**1.33 V**	**1.43 V**	**1.53 V**	**1.63 V**
1.75	1.12	1.22	0.83	0.52	0.42	0.32	0.22	0.12

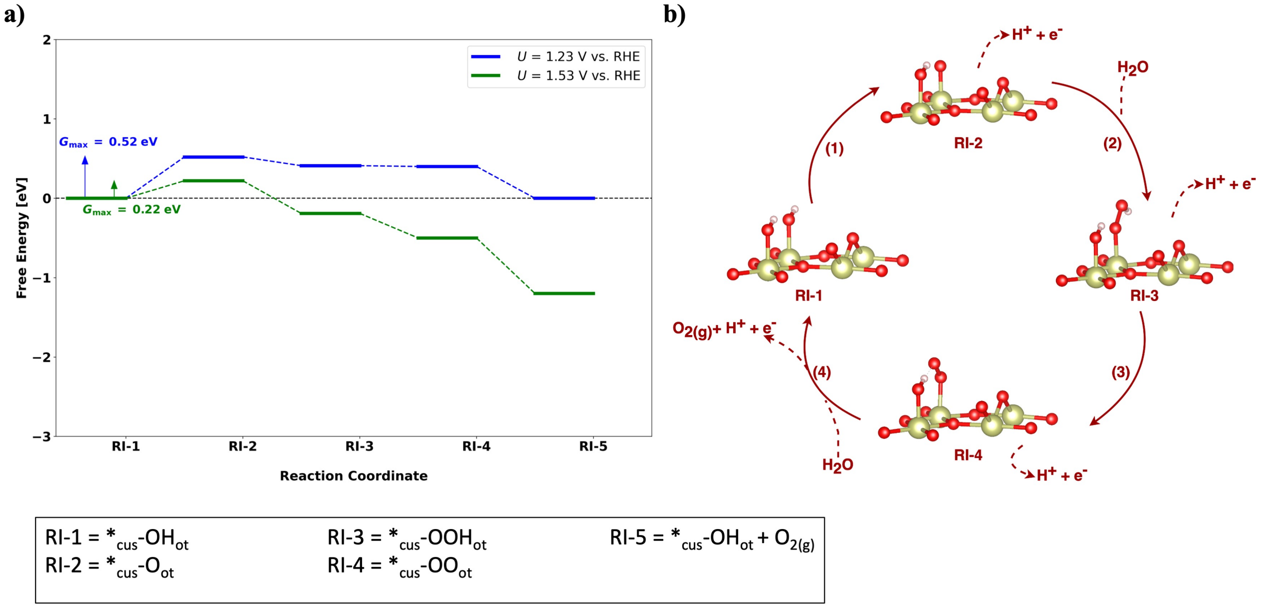

Figure S24. a) Free-energy diagram for the mononuclear-Walden mechanism on the partly hydroxylated $IrO_2(110)$ surface at 1.23 V and 1.53 V vs. RHE. The reaction intermediates of the mechanistic cycle are labeled on the x-axis. Blue and green solid lines indicate intermediates' free energies at 1.23 V and 1.53 V, respectively. Colored arrows indicate the free-energy span governing $G_{max}(U)$, with the respective value displayed.
b) Schematic illustration of the mononuclear-Walden mechanism, as described in Section 8.1, on the partly hydroxylated $IrO_2(110)$ surface. Numbers next to the arrows indicate the step sequence, and each structure represents the corresponding reaction intermediate.

10.2 Bifunctional-Walden mechanism

Table S28 compiles the free-energy changes for each elementary step at $U = 0$ V vs. RHE and the activity descriptor $G_{max}(U)$ at different applied electrode potentials under OER conditions. The corresponding free-energy diagram is depicted in Figure S25a, with a visual representation of the elementary steps in Figure S25b. The activity descriptor $G_{max}(U)$ is governed by the span $*_{cus}$-OH_{ot} + O_{br} → $*_{cus}$-O_{ot} + O_{br} in the potential range of $U = 1.23$ V to 1.53 V vs. RHE.

Table S28. Energetic evaluation of the bifunctional-Walden mechanism on the partly hydroxylated $IrO_2(110)$ surface (cf. Figure 1c in the main text) by the framework of the descriptor $G_{max}(U)$. The table indicates the free-energy changes of each step at $U = 0$ V vs. RHE and $G_{max}(U)$ values at different applied electrode potentials (U).

ΔG_1 [eV]	ΔG_2 [eV]	ΔG_3 [eV]	ΔG_4 [eV]	$G_{max}(U)$ [eV]				
				**1.23 V**	**1.33 V**	**1.43 V**	**1.53 V**	**1.63 V**
1.75	1.18	1.16	0.83	0.52	0.42	0.32	0.22	0.12

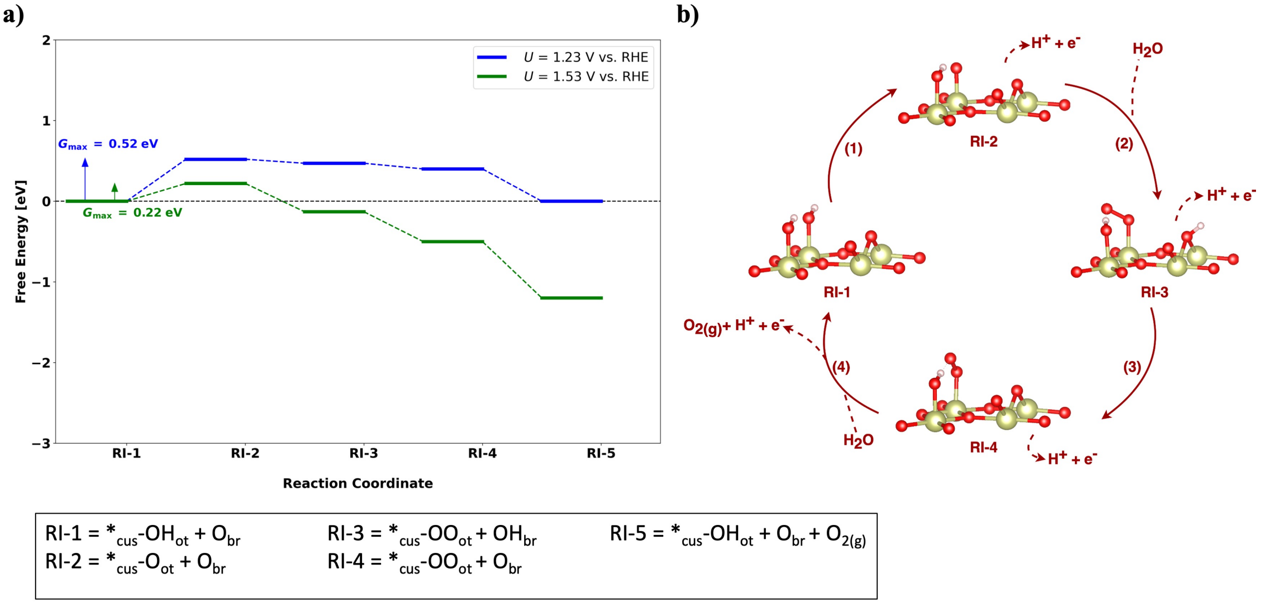

Figure S25. a) Free-energy diagram for the bifunctional-Walden mechanism on the partly hydroxylated $IrO_2(110)$ surface at 1.23 V and 1.53 V vs. RHE. The reaction intermediates of the mechanistic cycle are labeled on the x-axis. Blue and green solid lines indicate intermediates' free energies at 1.23 V and 1.53 V, respectively. Colored arrows indicate the free-energy span governing $G_{max}(U)$, with the respective value displayed.
b) Schematic illustration of the bifunctional-Walden mechanism, as described in Section 8.2, on the partly hydroxylated $IrO_2(110)$ surface. Numbers next to the arrows indicate the step sequence, and each structure represents the corresponding reaction intermediate.

11 Walden pathways over the fully oxygen-covered $IrO_2(110)$ surface

11.1 Mononuclear-Walden mechanism

Table S29 compiles the free-energy changes for each elementary step at $U = 0$ V vs. RHE and the activity descriptor $G_{max}(U)$ at different applied electrode potentials under OER conditions. The corresponding free-energy diagram is depicted in Figure S26a, with a visual representation of the elementary steps in Figure S26b. The activity descriptor $G_{max}(U)$ is governed by the span $*_{cus}$-$OH_{ot} \rightarrow *_{cus}$-$O_{ot} \rightarrow *_{cus}$-$OOH_{ot}$ for $U = 1.23$ V vs. RHE, and for larger overpotentials the limiting span switches to $*_{cus}$-$OH_{ot} \rightarrow *_{cus}$-$O_{ot}$.

Table S29. Energetic evaluation of the mononuclear-Walden mechanism on the fully oxygen-covered $IrO_2(110)$ surface (cf. Figure 1d in the main text) by the framework of the descriptor $G_{max}(U)$. The table indicates the free-energy changes of each step at $U = 0$ V vs. RHE and $G_{max}(U)$ values at different applied electrode potentials (U).

**ΔG_1** [eV]	**ΔG_2** [eV]	**ΔG_3** [eV]	**ΔG_4** [eV]	**$G_{max}(U)$** [eV]				
				**1.23 V**	**1.33 V**	**1.43 V**	**1.53 V**	**1.63 V**
1.66	1.33	1.12	0.80	0.53	0.33	0.23	0.13	0.03

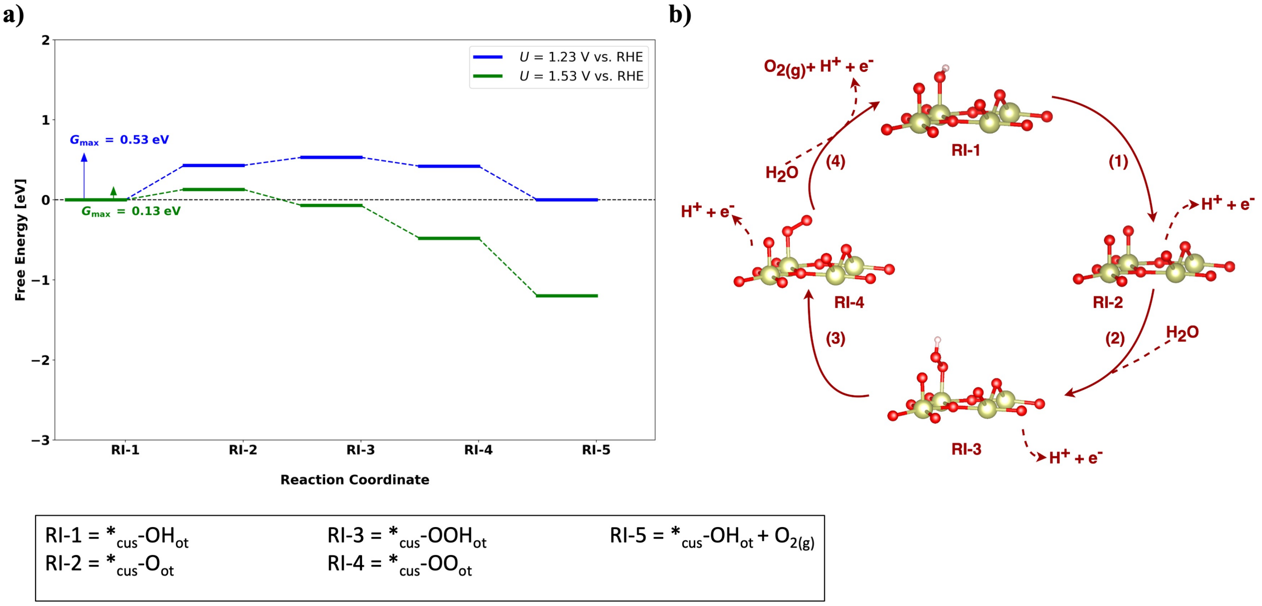

Figure S26. a) Free-energy diagram for the mononuclear-Walden mechanism on the fully oxygen-covered $IrO_2(110)$ surface at 1.23 V and 1.53 V vs. RHE. The reaction intermediates of the mechanistic cycle are labeled on the x-axis. Blue and green solid lines indicate intermediates' free energies at 1.23 V and 1.53 V, respectively. Colored arrows indicate the free-energy span governing $G_{max}(U)$, with the respective value displayed.
b) Schematic illustration of the mononuclear-Walden mechanism, as described in Section 8.1, on the fully oxygen-covered $IrO_2(110)$ surface. Numbers next to the arrows indicate the step sequence, and each structure represents the corresponding reaction intermediate.

11.2 Bifunctional-Walden mechanism

Table S30 compiles the free-energy changes for each elementary step at $U = 0$ V vs. RHE and the activity descriptor $G_{max}(U)$ at different applied electrode potentials under OER conditions. The corresponding free-energy diagram is depicted in Figure S27a, with a visual representation of the elementary steps in Figure S27b. The activity descriptor $G_{max}(U)$ is governed by the span $*_{cus}$-OH_{ot} + O_{br} → $*_{cus}$-O_{ot} + O_{br} → $*_{cus}$-OO_{ot}+ OH_{br} for $U = 1.23$ V vs. RHE, and for larger overpotentials the limiting span switches to $*_{cus}$-OH_{ot} + O_{br} → $*_{cus}$-O_{ot} + O_{br}.

Table S30. Energetic evaluation of the bifunctional-Walden mechanism on the fully oxygen-covered $IrO_2(110)$ surface (cf. Figure 1d in the main text) by the framework of the descriptor $G_{max}(U)$. The table indicates the free-energy changes of each step at $U = 0$ V vs. RHE and $G_{max}(U)$ values at different applied electrode potentials (U).

ΔG_1 [eV]	ΔG_2 [eV]	ΔG_3 [eV]	ΔG_4 [eV]	$G_{max}(U)$ [eV]				
				**1.23 V**	**1.33 V**	**1.43 V**	**1.53 V**	**1.63 V**
1.66	1.32	1.13	0.80	0.52	0.33	0.23	0.13	0.03

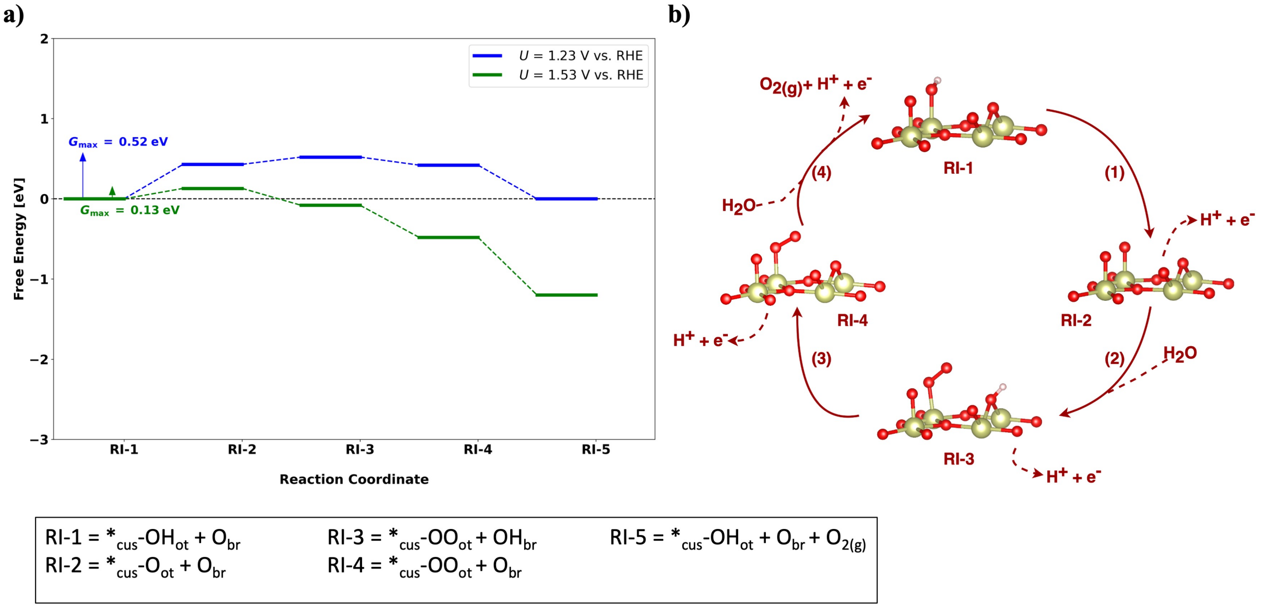

Figure S27. a) Free-energy diagram for the bifunctional-Walden mechanism on the fully oxygen-covered $IrO_2(110)$ surface at 1.23 V and 1.53 V vs. RHE. The reaction intermediates of the mechanistic cycle are labeled on the x-axis. Blue and green solid lines indicate intermediates' free energies at 1.23 V and 1.53 V, respectively. Colored arrows indicate the free-energy span governing $G_{max}(U)$, with the respective value displayed.
b) Schematic illustration of the bifunctional-Walden mechanism, as described in Section 8.2, on the fully oxygen-covered $IrO_2(110)$ surface. Numbers next to the arrows indicate the step sequence, and each structure represents the corresponding reaction intermediate.

12 Walden pathways over the partly OOH-covered $IrO_2(110)$ surface

12.1 Mononuclear-Walden mechanism

Table S31 compiles the free-energy changes for each elementary step at $U = 0$ V vs. RHE and the activity descriptor $G_{max}(U)$ at different applied electrode potentials under OER conditions. The corresponding free-energy diagram is depicted in Figure S28a, with a visual representation of the elementary steps in Figure S28b. The activity descriptor $G_{max}(U)$ is governed by the span $*_{cus}$-OH_{ot} → $*_{cus}$-O_{ot} → $*_{cus}$-OOH_{ot} in the potential range of $U = 1.23$ V to 1.53 V vs. RHE.

Table S31. Energetic evaluation of the mononuclear-Walden mechanism on the partly OOH-covered $IrO_2(110)$ surface (cf. Figure 1e in the main text) by the framework of the descriptor $G_{max}(U)$. The table indicates the free-energy changes of each step at $U = 0$ V vs. RHE and $G_{max}(U)$ values at different applied electrode potentials (U).

**ΔG_1** [eV]	**ΔG_2** [eV]	**ΔG_3** [eV]	**ΔG_4** [eV]	**$G_{max}(U)$** [eV]				
				**1.23 V**	**1.33V**	**1.43 V**	**1.53 V**	**1.63 V**
1.88	2.19	0.71	0.13	1.61	1.41	1.21	1.01	0.81

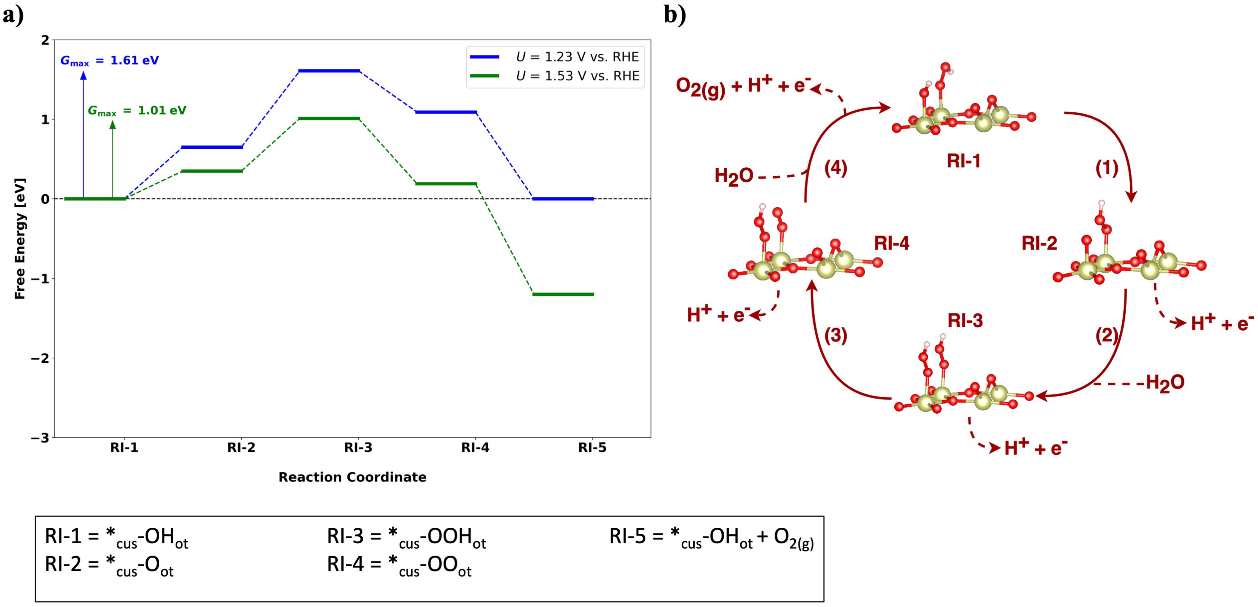

Figure S28. a) Free-energy diagram for the mononuclear-Walden mechanism on the partly OOH-covered $IrO_2(110)$ surface at 1.23 V and 1.53 V vs. RHE. The reaction intermediates of the mechanistic cycle are labeled on the x-axis. Blue and green solid lines indicate intermediates' free energies at 1.23 V and 1.53 V, respectively. Colored arrows indicate the free-energy span governing $G_{max}(U)$, with the respective value displayed.
b) Schematic illustration of the mononuclear-Walden mechanism, as described in Section 8.1, on the partly OOH-covered $IrO_2(110)$ surface. Numbers next to the arrows indicate the step sequence, and each structure represents the corresponding reaction intermediate.

12.2 Bifunctional-Walden mechanism

Table S32 compiles the free-energy changes for each elementary step at $U = 0$ V vs. RHE and the activity descriptor $G_{max}(U)$ at different applied electrode potentials under OER conditions. The corresponding free-energy diagram is depicted in Figure S29a, with a visual representation of the elementary steps in Figure S29b. The activity descriptor $G_{max}(U)$ is governed by the span $*_{cus}$-$OH_{ot} + O_{br} \rightarrow *_{cus}$-$O_{ot} + O_{br} \rightarrow *_{cus}$-$OO_{ot} + OH_{br} \rightarrow *_{cus}$-$OO_{ot} + O_{br}$ for $U = 1.23$ V vs. RHE, and for larger overpotentials the limiting span switches to $*_{cus}$-$OH_{ot} + O_{br} \rightarrow *_{cus}$-$O_{ot} + O_{br}$.

Table S32. Energetic evaluation of the bifunctional-Walden mechanism on the partly OOH-covered $IrO_2(110)$ surface (cf. Figure 1e in the main text) by the framework of the descriptor $G_{max}(U)$. The table indicates the free-energy changes of each step at U = 0 V vs. RHE and $G_{max}(U)$ values at different applied electrode potentials (U).

**ΔG_1** [eV]	**ΔG_2** [eV]	**ΔG_3** [eV]	**ΔG_4** [eV]	**$G_{max}(U)$** [eV]				
				**1.23 V**	**1.33 V**	**1.43 V**	**1.53 V**	**1.63 V**
1.88	1.35	1.55	0.13	1.09	0.79	0.49	0.35	0.25

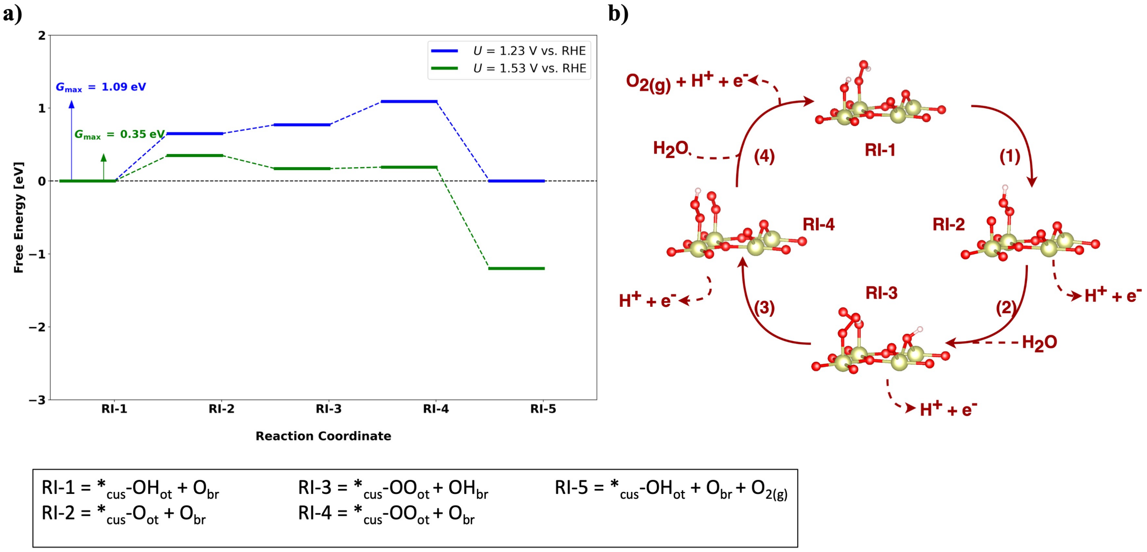

Figure S29. a) Free-energy diagram for the bifunctional-Walden mechanism on the partly OOH-covered $IrO_2(110)$ surface at 1.23 V and 1.53 V vs. RHE. The reaction intermediates of the mechanistic cycle are labeled on the x-axis. Blue and green solid lines indicate intermediates' free energies at 1.23 V and 1.53 V, respectively. Colored arrows indicate the free-energy span governing $G_{max}(U)$, with the respective value displayed.
b) Schematic illustration of the bifunctional-Walden mechanism, as described in Section 8.2, on the partly OOH-covered $IrO_2(110)$ surface. Numbers next to the arrows indicate the step sequence, and each structure represents the corresponding reaction intermediate.

13 Thermodynamically stable surface of $IrO_2(110)$ at U = 1.53 V vs. RHE

Table S33 summaries our extensive literature survey, in which we compiled the thermodynamically most stable surface configuration at U = 1.53 V vs. RHE depending on the exchange-correlation functional and the description of solvation used in the DFT framework.

Table S33. Summary of the thermodynamically stable surfaces of $IrO_2(110)$ at U = 1.53 V vs. RHE as reported in previous studies[24,27,38–47], together with the computational methods and solvation models used. The inclusion of "vdW" indicates that dispersion interactions were considered in the computational setup.

	Thermodynamically stable surface @ U = 1.53 V vs. RHE	Exchange correlation functional	Description of solvation
**Ref. 24**	Fully Oxygen-Covered	PBE + vdW	Implicit
**Ref. 27**	Fully Oxygen-Covered	PBE + vdW	Implicit
**Ref. 38**	Partly OOH-terminated	RPBE	No
**Ref. 39**	Partly OOH-terminated	BEEF-vdW	No

**Ref. 40**	Partly OOH-terminated	RPBE	Explicit
**Ref. 41**	Partly OOH-terminated	RPBE + vdW	Explicit
**Ref. 42**	Fully Oxygen-Covered	PBE	No
**Ref. 43**	Partly Hydroxylated	PBE + vdW	Implicit
**Ref. 44**	Fully Oxygen-Covered	PBE + vdW	Implicit
**Ref. 45**	Partly Hydroxylated	PBE + vdW	Implicit & Explicit
**Ref. 46**	Fully Oxygen-Covered	PBE + vdW	Implicit
**Ref. 47 (*)**	Fully Oxygen-Covered	PBE + vdW	No
**This Work**	Partly Hydroxylated	PBE + vdW	Implicit

*The applied electrode potential in this work is 1.20 V vs. RHE instead of 1.53 V vs. RHE.

As evident from Table S33, different surface phases ranging from a partially hydroxylated to a partially OOH-terminated surface are energetically favored at U = 1.53 V vs. RHE, and the full range of possible surface configurations is considered in the mechanistic analysis presented in this work.

14 OER on the bridge sites of IrO_2(110)

While it has been reported in previous works[20,21,24,27,42,48–50] that the cus sites are the catalytically active centers for the OER or other surface reactions on IrO_2(110) whereas the bridge sites are mainly spectators, we further investigate the electrocatalytic activity of the bridge sites of the IrO_2(110) surface using fully oxygen-covered IrO_2(110) surface (cf. Figure 1d of the main text) as a representative example. The mononuclear mechanism of the OER on an Ir bridge site ($*_{br}$) of IrO_2(110) reads:

$$*_{br} + H_2O \rightarrow *_{br}\text{-}OH_{br} + H^+ + e^- \quad (48)$$

$$*_{br}\text{-}OH_{br} \rightarrow *_{br}\text{-}O_{br} + H^+ + e^- \quad (49)$$

$$*_{br}\text{-}O_{br} + H_2O \rightarrow *_{br}\text{-}OOH_{br} + H^+ + e^- \quad (50)$$

$$*_{br}\text{-}OOH_{br} \rightarrow *_{br} + O_{2(g)} + H^+ + e^- \quad (51)$$

While Table S34 compiles the free-energy changes for each elementary step at U = 0 V vs. RHE and the activity descriptor $G_{max}(U)$ at different applied electrode potentials under OER conditions, the corresponding free-energy diagram is depicted in Figure S30a, with a visual representation of the elementary steps in Figure S30b. While the activity descriptor $G_{max}(U)$ is governed by the span $*_{br}$-OH_{br} → $*_{br}$-O_{br} → $*_{br}$-OOH_{br} → $*_{br}$ + O_2 at U = 1.23 V vs. RHE, the limiting free-energy span switches to $*_{br}$-O_{br} → $*_{br}$-OOH_{br} → $*_{br}$ + O_2 for larger applied overpotentials (U = 1.53 V vs. RHE).

Based on the determination of the activity descriptor $G_{max}(U$ = 1.53 V) = 1.86 eV for the mononuclear mechanism, which exceeds the value for the Ir cus site ($G_{max}(U$ = 1.53 V) = 0.13 eV for the energetically favored mononuclear-Walden mechanism) by more than 1.70 eV, the Ir bridge site can be fairly excluded as an active site in the OER over IrO_2(110).

Table S34. Energetic evaluation of the mononuclear mechanism on the bridge site of the fully oxygen-covered $IrO_2(110)$ surface (cf. Figure 1d in the main text) by the framework of the descriptor $G_{max}(U)$. The table indicates the free-energy changes of each step at $U = 0$ V vs. RHE and $G_{max}(U)$ values at different applied electrode potentials (U).

ΔG_1 [eV]	ΔG_2 [eV]	ΔG_3 [eV]	ΔG_4 [eV]	$G_{max}(U)$ [eV]				
				**1.23 V**	**1.33 V**	**1.43 V**	**1.53 V**	**1.63 V**
-1.29	1.29	2.00	2.92	2.52	2.26	2.06	1.86	1.66

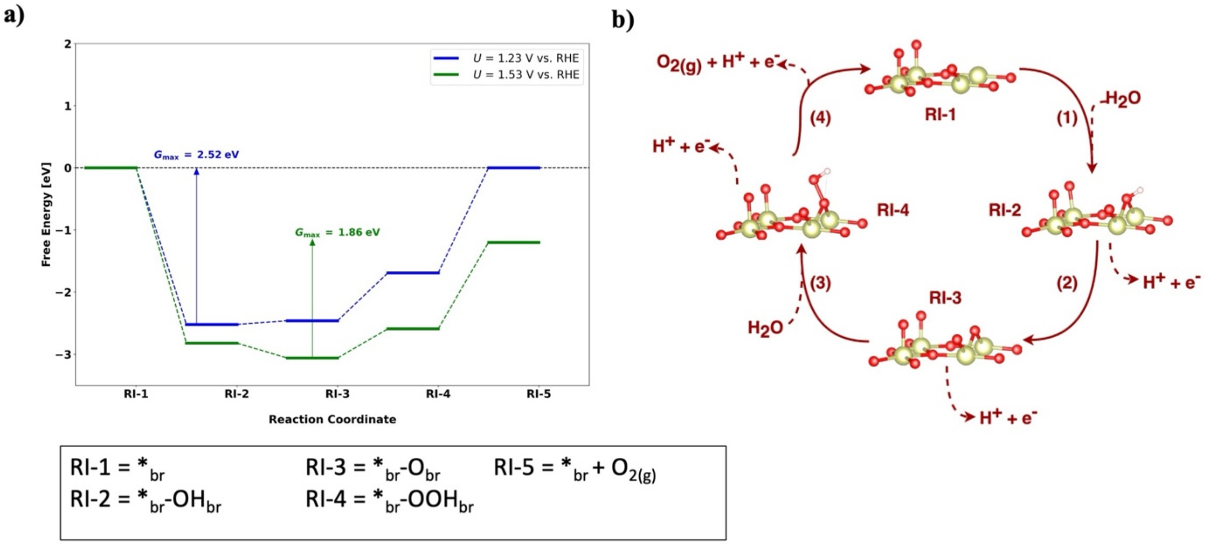

Figure S30. a) Free-energy diagram for the mononuclear mechanism on the bridge site of the fully oxygen-covered $IrO_2(110)$ surface at 1.23 V and 1.53 V vs. RHE. The reaction intermediates of the mechanistic cycle are labelled on the x-axis. Blue and green solid lines indicate intermediates' free energies at 1.23 V and 1.53 V, respectively. Colored arrows indicate the free-energy span governing $G_{max}(U)$, with the respective value displayed.
b) Schematic illustration of the mononuclear mechanism, as described in Section 3.1, on the bridge site of the fully oxygen-covered $IrO_2(110)$ surface. Numbers next to the arrows indicate the step sequence, and each structure represents the corresponding reaction intermediate.

Besides the mononuclear mechanism, we have also evaluated the energetics of the mononuclear-Walden pathway at the Ir bridge site. The mechanistic description is given by equations (52) – (55):

$$*_{br}\text{-}OH_{br} \rightarrow *_{br}\text{-}O_{br} + H^+ + e^- \quad (52)$$

$$*_{br}\text{-}O_{br} + H_2O \rightarrow *_{br}\text{-}OOH_{br} + H^+ + e^- \quad (53)$$

$$*_{br}\text{-}OOH_{br} \rightarrow *_{br}\text{-}OO_{br} + H^+ + e^- \quad (54)$$

$$*_{br}\text{-}OO_{br} + H_2O \rightarrow *_{br}\text{-}OH_{br} + O_{2(g)} + H^+ + e^- \quad (55)$$

While Table S35 compiles the free-energy changes for each elementary step at $U = 0$ V vs. RHE and the activity descriptor $G_{max}(U)$ at different applied electrode potentials under OER conditions, the corresponding free-energy diagram is depicted in Figure S31a, with a visual

representation of the elementary steps in Figure S31b. While the activity descriptor $G_{max}(U)$ is governed by the span $*_{br}$-OH_{br} → $*_{br}$-O_{br} → $*_{br}$-OOH_{br} at U = 1.23 V vs. RHE, the limiting free-energy span switches to $*_{br}$-O_{br} → $*_{br}$-OOH_{br} for larger applied overpotentials (U = 1.53 V vs. RHE).

Table S35. Energetic evaluation of the mononuclear-Walden mechanism on the bridge site of the fully oxygen-covered IrO_2(110) surface (cf. Figure 1d in the main text) by the framework of the descriptor $G_{max}(U)$. The table indicates the free-energy changes of each step at U = 0 V vs. RHE and $G_{max}(U)$ values at different applied electrode potentials (U).

ΔG_1 [eV]	ΔG_2 [eV]	ΔG_3 [eV]	ΔG_4 [eV]	$G_{max}(U)$ [eV]				
				**1.23 V**	**1.33 V**	**1.43 V**	**1.53 V**	**1.63 V**
1.29	2.00	1.21	0.42	0.83	0.67	0.57	0.47	0.37

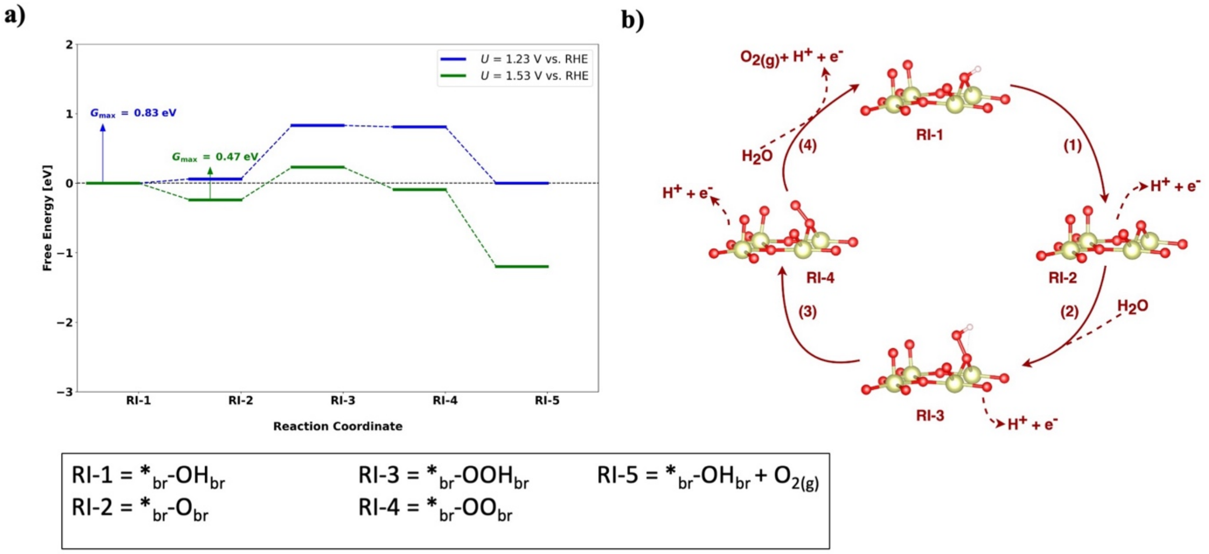

Figure S31. a) Free-energy diagram for the mononuclear-Walden mechanism on the bridge site of fully oxygen-covered IrO_2(110) surface at 1.23 V and 1.53 V vs. RHE. The reaction intermediates of the mechanistic cycle are labeled on the x-axis. Blue and green solid lines indicate intermediates' free energies at 1.23 V and 1.53 V, respectively. Colored arrows indicate the free-energy span governing $G_{max}(U)$, with the respective value displayed.
b) Schematic illustration of the mononuclear-Walden mechanism, as described in Section 8.1, on the bridge site of the fully oxygen-covered IrO_2(110) surface. Numbers next to the arrows indicate the step sequence, and each structure represents the corresponding reaction intermediate.

We note that the mononuclear-Walden mechanism reveals a significantly lower $G_{max}(U)$ value compared to the mononuclear mechanism at the Ir bridge site. This result is in qualitative agreement to the discussion of the Walden-type pathways at the Ir cus site in the main text of our work. This underlines the scope of Walden-like pathways for electrocatalytic processes, as concerted desorption-adsorption processes can efficiently reduce thermodynamic constraints in proton-coupled electron transfer steps.

Despite the reduced $G_{max}(U)$ value, the electrocatalytic activity of the Ir bridge site is still sufficiently lower ($G_{max}(U$ = 1.53 V) = 0.47 eV) compared to the Ir cus site ($G_{max}(U$ = 1.53 V) =

0.13 eV). Therefore, we conclude that the bridge sites are not the active sites in the OER over $IrO_2(110)$, which is in line with previous works on the topic[20,21,24,27,42,48–50].

15 Kinetics of O_2 desorption

To investigate the kinetics of O_2 desorption for conventional and Walden-type pathways, we reformulate the mononuclear mechanism by including the *OO adsorbate as well as the chemical desorption of O_2 as an additional step into the analysis:

$$*_{cus} + H_2O \rightarrow *_{cus}\text{-}OH_{ot} + H^+ + e^- \qquad (56)$$

$$*_{cus}\text{-}OH_{ot} \rightarrow *_{cus}\text{-}O_{ot} + H^+ + e^- \qquad (57)$$

$$*_{cus}\text{-}O_{ot} + H_2O \rightarrow *_{cus}\text{-}OOH_{ot} + H^+ + e^- \qquad (58)$$

$$*_{cus}\text{-}OOH_{ot} \rightarrow *_{cus}\text{-}OO_{ot} + H^+ + e^- \qquad (59)$$

$$*_{cus}\text{-}OO_{ot} \rightarrow *_{cus} + O_{2(g)} \qquad (60)$$

The corresponding free-energy diagram at U = 1.53 V vs. RHE for equations (56) – (60) is shown in Figure S32, using the partly hydroxylated $IrO_2(110)$ surface (cf. Figure 1c in the main text) as a representative example. In addition, we plot the free-energy diagram for the traditional mononuclear mechanism (where steps 59 and 60 are combined) in Figure S32b.

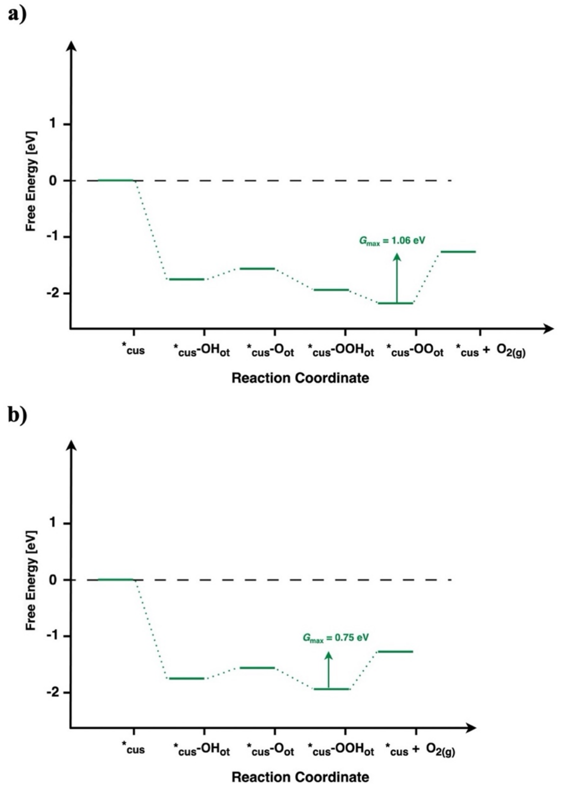

Figure S32. a) Free-energy diagram of the OER over the partly hydroxylated $IrO_2(110)$ surface according to the mechanistic description in equations (56) – (60) at U = 1.53 V vs. RHE. b) Free-energy diagram of the OER over the partly hydroxylated $IrO_2(110)$ surface for the traditional mononuclear mechanism at U = 1.53 V vs. RHE.

To compare the energetics of the refined mononuclear mechanism containing a chemical step (cf. equation (60) with the energetics of the Walden-type pathway, we refine the mechanistic description of the mononuclear-Walden mechanism by considering a chemical step for the desorption of O_2 (cf. equation (64). Therefore, we consider the $*_{cus}$-$(OH_2)_{ot}$ intermediate as an additional adsorbate in our analysis:

$$*_{cus}\text{-}OH_{ot} \rightarrow *_{cus}\text{-}O_{ot} + H^+ + e^- \quad (61)$$

$$*_{cus}\text{-}O_{ot} + H_2O \rightarrow *_{cus}\text{-}OOH_{ot} + H^+ + e^- \quad (62)$$

$$*_{cus}\text{-}OOH_{ot} \rightarrow *_{cus}\text{-}OO_{ot} + H^+ + e^- \quad (63)$$

$$*_{cus}\text{-}OO_{ot} + H_2O \rightarrow *_{cus}\text{-}(OH_2)_{ot} + O_{2(g)} \quad (64)$$

$$*_{cus}\text{-}(OH_2)_{ot} \rightarrow *_{cus}\text{-}OH_{ot} + H^+ + e^- \quad (65)$$

The corresponding free-energy diagram at U = 1.53 V vs. RHE for equations (61) – (65) is shown in Figure S33a, using the partly hydroxylated $IrO_2(110)$ surface (cf. Figure 1c in the main text) as a representative example. In addition, we plot the free-energy diagram for the original description of the mononuclear-Walden pathway (where steps 64 and 65 are combined) in Figure S33b.

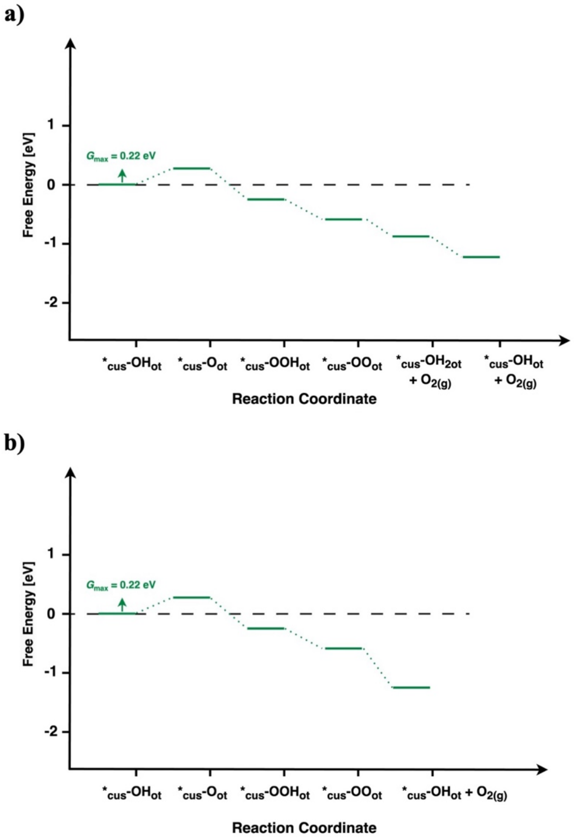

Figure S33. a) Free-energy diagram of the OER over the partly hydroxylated $IrO_2(110)$ surface according to the mechanistic description in equations (61) – (65) at U = 1.53 V vs. RHE. b) Free-energy diagram of the OER over the partly hydroxylated $IrO_2(110)$ surface for the mononuclear-Walden mechanism at U = 1.53 V vs. RHE.

When comparing the thermodynamic free-energy landscapes of Figure S32-S33, we observe that the electrocatalytic activity for the Walden-type pathway is not affected by the consideration of the $*_{cus}$-$(OH_2)_{ot}$ intermediate in the analysis: the descriptor $G_{max}(U)$ amounts to 0.22 eV, and the limiting free-energy span is $*_{cus}$-OH_{ot} → $*_{cus}$-O_{ot} at U = 1.53 V vs. RHE. In contrast, the consideration of an additional chemical step by means of the *OO adsorbate for the mononuclear mechanism leads to a significant increase in the descriptor $G_{max}(U)$ from 0.75 eV to 1.06 eV, indicating that the Walden pathway is clearly favored over the traditional mechanism when using the span model as a measure for the electrocatalytic activity.

In the following, we provide evidence by the calculation of transition states that the water-mediated desorption of O_2 is kinetically more facile than the desorption of O_2 without the direct adsorption of water. To this end, we performed nudge elastic band (NEB) calculations to map the reaction pathway and locate an approximate transition state for equations (60) and (64). Subsequently, the dimer method was employed to refine and identify the precise transition state structure. The identified transition state was validated by frequency analysis. All these calculations were performed including spin polarization and an implicit solvation model (VASPsol).

To calculate the transition state of equations (60) and (64), we rely on a canonical framework to ensure consistency with the thermodynamic analysis of the reaction intermediates' free energies. This is further backed up by the fact that Ping and Goddard[24] reported that the difference between constant potential and constant charge calculations for $IrO_2(110)$ in their study did not exceed 0.10 eV, which is considered to be sufficiently accurate for the present qualitative assessment of the Walden transition state. While Figure S34 illustrates the initial, transition, and final states for the process of equation (60), Table S36 summarizes the energetics of the states involved.

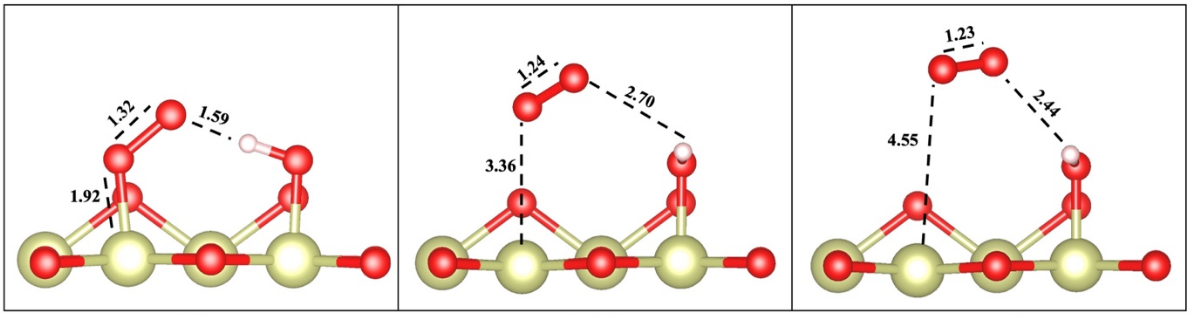

Figure S34. Initial (IS), transition (TS), and final (FS) states for O_2 desorption (cf. equation (60) from the partly hydroxylated $IrO_2(110)$ surface. All bond lengths are given in Å.

Table S36. Energetics of the initial (IS), transition (TS), and final (FS) states of O_2 desorption (cf. equation (60) from the partly hydroxylated $IrO_2(110)$ surface to derive the activation free energy, $\Delta G^{\#}$, for this process.

	E_{DFT} [eV]	ZPE [eV]	TS [eV]
**IS**	–434.66	0.52	0.20
**TS**	–433.38	0.47	0.31
**FS**	–433.34	0.48	0.24
**G_{IS} [eV]**	–434.34		
**G_{TS} [eV]**	–433.22		
**$\Delta G^{\#}$ [eV]**	**1.12**		

In the same fashion, we investigate the transition state for the water-mediated Walden-type step. While Figure S35 illustrates the initial, transition, and final states for the process of equation (64), Table S37 summarizes the energetics of the states involved.

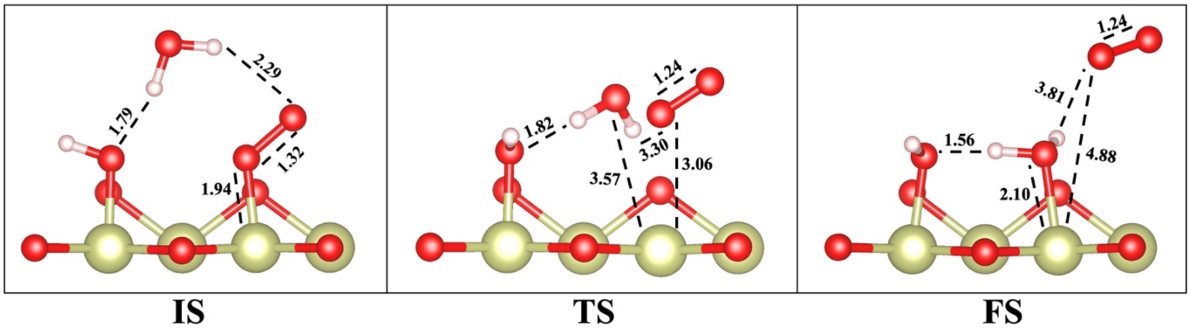

Figure S35. Initial (IS), transition (TS), and final (FS) states for the water-mediated O_2 desorption (cf. equation (64) from the partly hydroxylated $IrO_2(110)$ surface. All bond lengths are given in Å.

Table S37. Energetics of the initial (IS), transition (TS), and final (FS) states of water-mediated O_2 desorption (cf. equation (64) from the partly hydroxylated $IrO_2(110)$ surface to derive the activation free energy, $\Delta G^{\#}$, for this process.

	E_{DFT} [eV]	ZPE [eV]	TS [eV]
**IS**	–449.43	1.16	0.32
**TS**	–448.31	1.15	0.38
**FS**	–449.47	1.14	0.31
**G_{IS} [eV]**	–448.59		
**G_{TS} [eV]**	–447.54		
**$\Delta G^{\#}$ [eV]**	**1.05**		

Our analysis reveals that the desorption barrier of O_2 is on the order of about 1 eV, and the activation barrier for the water-assisted route in the framework of a Walden step (cf. equation (64) is 0.07 eV lower in free energy than the desorption of O_2 without the involvement of a water molecule (cf. equation (60). These activation free energies are incorporated into the free-energy landscapes of the OER on $IrO_2(110)$, which is shown in Figure 5 of the main text.

16 Comparison with experimental data

We performed microkinetic simulations based on the evaluation of the descriptor $G_{max}(U)$[30,31] to estimate the current density (j) as a function of the applied electrode potential for different surface configurations and reaction mechanisms (cf. Table S38), which can be compared to the experimentally measured current density by Suntivich and coworkers for an $IrO_2(110)$ model electrode[51,52]. In our analysis, we use an applied electrode potential of U = 1.60 V vs. RHE (reversible hydrogen electrode) to link experimental and theoretical investigations.

Following previous work on the descriptor $G_{max}(U)$[30,31], this activity measure allows the determination of the current density using equation (1):

$$j(U) = \frac{4k_BT}{h} e\Gamma_{act} e^{\left[\frac{-(G_{max}(U)+\beta)}{k_BT}\right]} \quad (66)$$

In equation (66), e, k_B, T, and h denote the elementary charge, Boltzmann constant, absolute temperature in Kelvin, and Planck constant, respectively, while the density of active surface site

(cus sites) amounts to Γ_{act} = 7 x 10^{14} cm^{-2} for $IrO_2(110)$. β refers to the Brønsted-Evans-Polanyi (BEP) intercept constant, which links the thermodynamic analysis in terms of $G_{max}(U)$ with the kinetics related to the transition-state free energy. Based on previous work on the descriptor $G_{max}(U)$[30,31], β = 0.6 is chosen in the analysis. Table S38 summarizes the estimated current densities at U = 1.60 V vs. RHE for the traditional and Walden mechanisms for four different surface configurations of $IrO_2(110)$ (cf. Figure 1b-e of the main text).

Table S38. Comparison of the theoretically calculated current densities (j) for the traditional and Walden-type OER mechanisms over four different $IrO_2(110)$ surface configurations at U = 1.60 V vs. RHE.

**Traditional Mechanism**	**U = 1.60 V vs. RHE**	**Walden-type Mechanism**	**U = 1.60 V vs. RHE**
	**Current Density (j) [mA/cm^2]**		**Current Density (j) [mA/cm^2]**
**Fully hydroxylated $IrO_2(110)$ surface**			
Mononuclear	8.9 x 10^{-12}	Mononuclear-Walden	28.70
**Partly hydroxylated $IrO_2(110)$ surface**			
Mononuclear	6.4 x 10^{-10}	Mononuclear-Walden	0.59
**Fully oxygen-covered $IrO_2(110)$ surface**			
Mononuclear	3.6 x 10^{-4}	Mononuclear-Walden	19.44
**Partly OOH-covered $IrO_2(110)$ surface**			
Mononuclear	2.6 x 10^{-14}	Mononuclear-Walden	2.6 x 10^{-14}

Considering that the experimental benchmark in the OER over $IrO_2(110)$ based on the work of Kuo et al[51,52]. amounts to $j \approx 0.01$ mA/cm^2 at U = 1.60 V vs. RHE, it is obvious that our theoretical model predicts current densities following the Walden-type pathway for the fully hydroxylated, partially hydroxylated and fully oxygen-covered $IrO_2(110)$ surface that are in the same order of magnitude as the experiments. In contrast, there is a strong difference in current density for the Walden mechanism over the partly OOH-covered $IrO_2(110)$ surface as well as for all traditional mechanisms over the different $IrO_2(110)$ surface configurations with respect to the experimental benchmark. Therefore, we conclude that the $IrO_2(110)$ surface is likely hydroxylated or covered with oxygen adsorbates under typical OER conditions, and our comparison with the experimental data further suggests the prevalence of Walden-type pathways over conventional OER mechanisms.

17 Comparison with a previous work by Binninger and Doublet

In a previous work, Binninger and Doublet reported that the OER on $IrO_2(110)$ proceeds via the oxide mechanism.[27] To reach this conclusion, the authors calculated the free-energy barrier of selected chemical reaction steps for a limited number of surface configurations and OER pathways. We note that this approach is different to the approach used in the present work. In the following, we point out the differences in the methodology between Binninger's and our study:

i) According to the Butler-Volmer theory[53], the rate of electrocatalytic processes is not determined by barriers but rather by transition states, combining the thermodynamic and kinetic contributions to the transition state with the highest free energy. Therefore, we do not discuss barriers in identifying the preferred mechanism or the limiting reaction step.

ii) To identify the transition state with the highest free energy, which determines the kinetic rate equation, the energetics of all transition states along the reaction coordinate must be calculated. We emphasize that no theoretical work has been able to tackle such a daunting task, as it requires the resolution of the transition states of all chemical and electrochemical steps in the OER catalytic cycle. Despite increasing computational power, this still exceeds the capabilities of theoretical studies by far due to the enormous computational effort required to identify transition states in an electrochemical environment.
Therefore, we conclude that current approaches in theoretical electrocatalysis for barrier calculations are not sufficient to capture the 'true kinetic picture' of the proton-coupled electron transfer steps. To this end, we rely on thermodynamic approaches rather than barrier calculations to infer the preferred mechanistic pathway.

iii) We argue that the discussion of barriers for chemical reaction steps in the OER over $IrO_2(110)$ does not seem reasonable if one wants to identify the preferred reaction mechanism. The reason for this is – as explained above – that chemical steps are not rate determining[49,51,52] and are therefore hidden in the kinetic rate equation, which is determined only by the transition state with the highest free energy.[53] Note that the transition state with the highest free energy in the OER over $IrO_2(110)$ is an electrochemical step and the transition states of electrochemical steps in the OER over $IrO_2(110)$ were not resolved in previous computational studies[21,24,27,42].

Based on the above discussion, we draw conclusions in our manuscript using thermodynamic approaches by making use of the descriptor $G_{max}(U)$,[30,31] which is further discussed in the Method section. To this end, we rely on thermodynamic information rather than the energetics of selected transition states when discussing OER on $IrO_2(110)$. Taking into account the caveats and reservations listed above regarding the discussion of barriers to chemical reaction steps only, we believe that the $G_{max}(U)$ approach – within the limitation of the BEP approximation – is fully consistent and robust to investigate the OER over $IrO_2(110)$.

Comparing the thermodynamic free-energy landscapes of the OER on $IrO_2(110)$, Figure S36 shows that the Walden-type pathway is clearly preferred over the oxide mechanism, as the $G_{max}(U)$ value is 0.44 eV smaller. Considering the sensitivity of 0.20 eV of the $G_{max}(U)$ descriptor,[30] it can be reasonably assumed that the Walden mechanism is superior to the oxide description.

a)

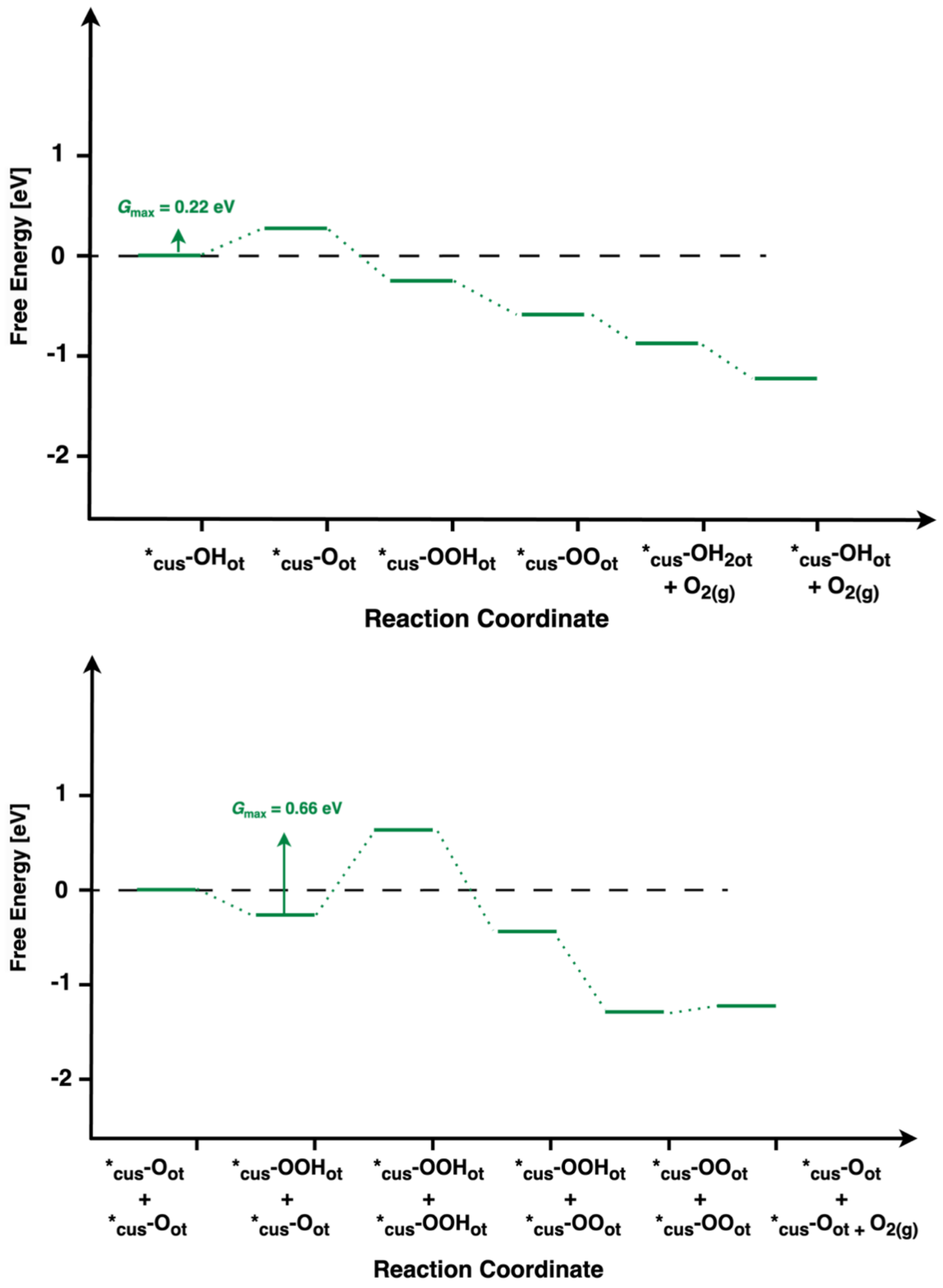

Figure S36. a) Thermodynamic free-energy diagram of the OER over the partly hydroxylated IrO2(110) surface for the Walden-type mechanism with water-assisted O_2 desorption at U = 1.53 V vs. RHE. b) Thermodynamic free-energy diagram of the OER over the fully oxygen-covered IrO_2(110) surface for the oxide mechanism with an Ir-OOOO-Ir association step at U = 1.53 V vs. RHE.

Figure S37 indicates the free-energy landscape of the OER for the Walden-type and oxide mechanisms considering the transition state for water-assisted O_2 desorption and the Ir-OOOO-Ir association step, respectively. When comparing the barrier heights of the transition states, it becomes clear that the Ir-OOOO-Ir association step is kinetically simpler.

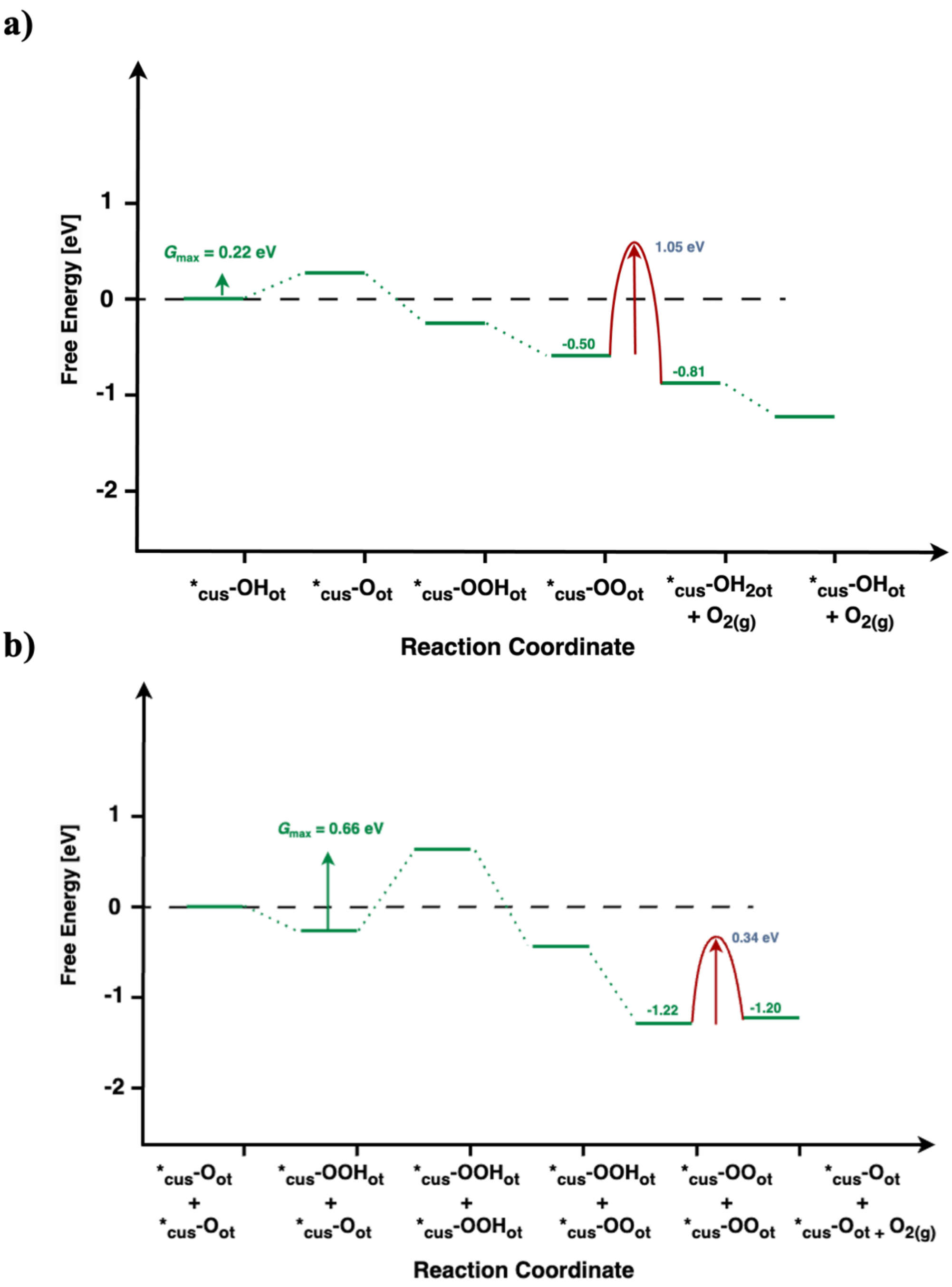

Figure S37. a) Thermodynamic free-energy diagram of the OER over the partly hydroxylated IrO2(110) surface for the Walden-type mechanism with water-assisted O_2 desorption at U = 1.53 V vs. RHE. The calculated activation free energy for the water-assisted desorption of the O_2 molecule has been added to the figure. **b)** Thermodynamic free-energy diagram of the OER over the fully oxygen-covered IrO_2(110) surface for the oxide mechanism with an Ir-OOOO-Ir association step at U = 1.53 V vs. RHE. The calculated activation free energy for the Ir-OOOO-Ir association step is taken from previous work by Binninger and Doublet.[27]

We argue that the actual barrier height of the water-assisted O_2 desorption or the Ir-OOOO-Ir association step is not relevant because none of these steps constitutes the rate-determining step

in the OER over IrO_2(110).[49,51,52] Therefore, these steps are hidden in the kinetic rate equation, which is determined only by the transition state with the highest free energy.[53]

By comparing the $G_{max}(U)$ descriptor, which is directly related to the transition state with the highest free energy, it becomes clear that the oxide mechanism is thermodynamically unfavorable due to the formation of two adjacent *OOH intermediates. Such a scenario is not observed for the Walden-type mechanism, which requires only a single *OOH intermediate. Therefore, we conclude that, despite the kinetically facile Ir-OOOO-Ir association step, the bottleneck of the oxide mechanism is due to the lateral interaction of the *OOH intermediates required for this pathway. For a further contemplation of the limiting factors, it would be required to calculate transition states for the formation of two adjacent *OOH intermediates. However, this is beyond the scope of the present manuscript, which focuses on the thermodynamic information related to the reaction intermediates by using the $G_{max}(U)$ descriptor.

It should also be noted that, even if the barrier of 1.05 eV for water-assisted O_2 desorption appears large (cf. Figure S37a), this barrier can be overcome for industrial operating conditions of 80 – 90 °C. However, we do not believe that a qualitative discussion of the water-assisted O_2 desorption barrier is justified, considering that previous work on the topic reported a significantly smaller barrier: following the previous work by Ping et al.,[24] there are no kinetic limitations for water-assisted O_2 desorption (free-energy barrier of 0.56 eV), even at room temperature.

To further investigate the correlation of the two reaction pathways, we have performed microkinetic simulations based on the evaluation of the descriptor $G_{max}(U)$[30,31] to estimate the current density (j) as a function of the applied electrode potential (U) for the oxide mechanism (cf. Table S39), which can be compared to the experimentally measured current density by Suntivich and coworkers for an IrO_2(110) model electrode.[51,52] We refer to section 16 of the SI for a discussion of the approach. Two β values are selected for the analysis: a) β = 0.60 eV and b) $G_{max}(U) + \beta = 0.34$ eV; the latter corresponding to the activation barrier reported for O_2 desorption by the *OO-OO* association step.[27] The results are listed in Table S39.

Table S39. Current densities (j) for the oxide mechanism over the fully oxygen-covered IrO_2(110) surface configuration at U = 1.60 V vs. RHE.

**Mechanism**	**BEP intercept constant *(β)***	***U* = 1.60 V vs. RHE**
		**Current Density (*j*) [mA/cm²]**
Oxide	0.60	2.14×10^{-8}
	0.34	4.99×10^{6}

Given that the experimental benchmark for OER over IrO_2(110), as reported by Kuo et al.,[51,52] is $j \approx 0.01$ mA/cm² at U = 1.60 V vs. RHE, it is evident that the current densities predicted for the oxide mechanism deviate significantly from this benchmark. On the contrary, the current densities derived for the Walden-type pathway approach the experimental current density for

single-crystal IrO_2(110) (cf. section 16 of the SI), thus confirming our conclusion that Walden-like mechanisms are favored in the OER.

Finally, we performed a Bader charge analysis for the oxide mechanism, which is shown in Figure S38. As discussed in section 2.4 of our manuscript, we link the largest charge span in the catalytic cycle – Q_{max} – to the electrocatalytic activity in terms of $G_{max}(U)$. Our analysis reveals that Q_{max} amounts to +0.23e for the oxide description, which is larger than the charge span for the Walden-type pathway of +0.17e (cf. Figure 6b of the main text). Therefore, this approach also confirms that the OER over IrO_2(110) is controlled by Walden-like mechanisms.

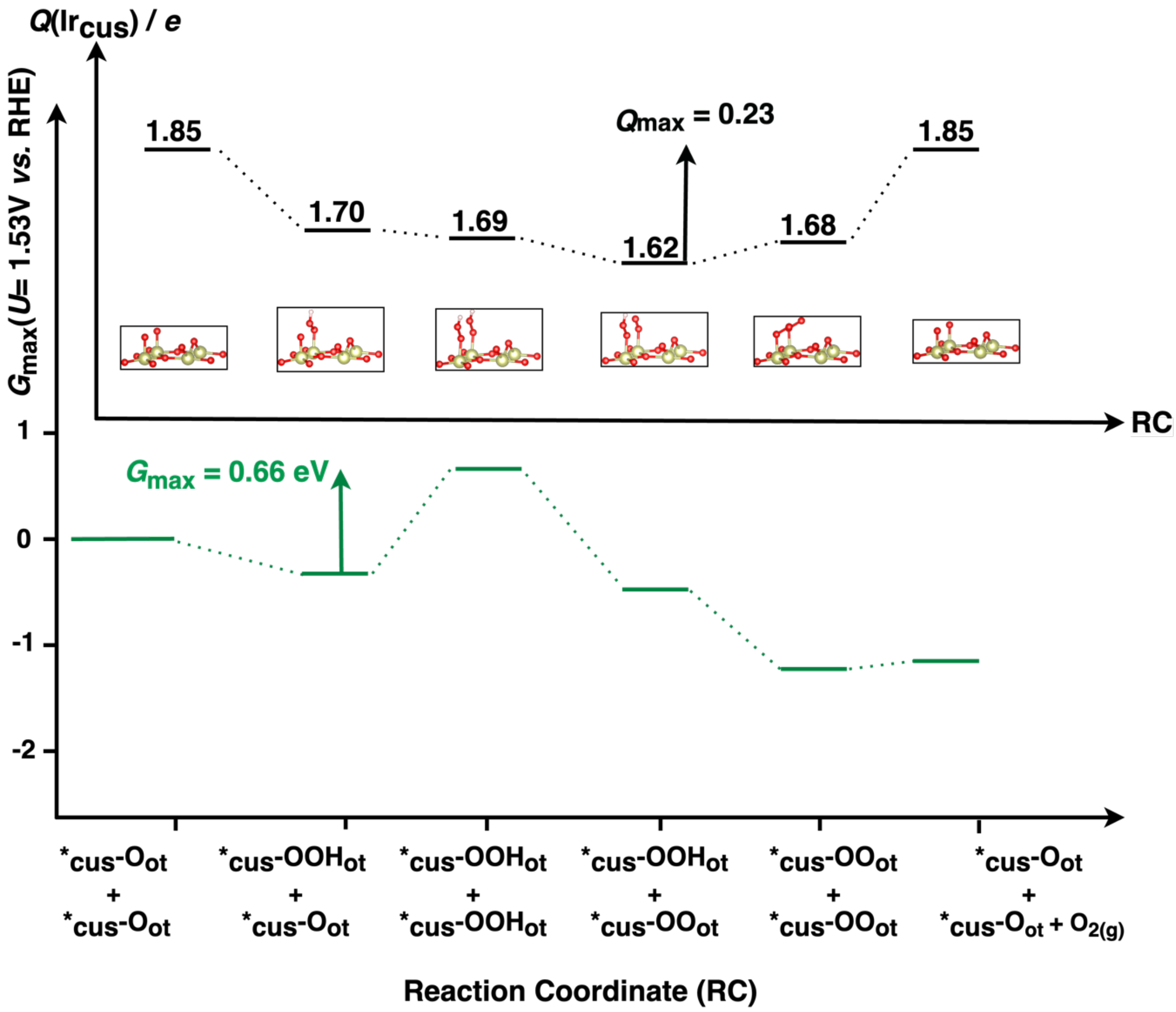

Figure S38. Charge states (upper panel) and free-energy diagram (lower panel) of the reaction intermediates in the OER over the fully oxygen-covered IrO_2(110) surface for the oxide mechanism at U = 1.53 V vs. RHE.

Ultimately, however, we must acknowledge that all current works in the field of theoretical electrocatalysis are based on approximations, since not all transition states can be resolved in the free-energy landscape. It can be interpreted as a matter of taste whether the thermodynamics – as done in the present contribution to a large extent – or the kinetics of selected steps is used for a discussion of the electrocatalytic activity. In our opinion, the discussion of transition states for

selected steps as an indicator of electrocatalytic activity is subject to a bias[54], since it assumes *a priori* which elementary step limits the reaction rate. In the end, one has to be aware of the fact that none of these approaches is entirely conclusive, since only knowledge of all transition states, coupled with a degree of rate control analysis, would allow us to draw definite conclusions about the electrocatalytic activity, limiting steps, and reaction mechanisms of proton-coupled electron transfer steps at electrified solid/ liquid interfaces. We believe that the community will continue to evolve in this direction over the next years, provided the determination of transition states in an electrochemical environment is facilitated by machine learning and artificial intelligence approaches. Currently, we believe that discussing the OER over $IrO_2(110)$ using the $G_{max}(U)$ approach is the best compromise for a consistent and unbiased evaluation of the electrocatalytic activity of a model system.

Supplementary References

1. Kresse, G. & Hafner, J. *Ab initio* molecular dynamics for liquid metals. *Phys. Rev. B* **47**, 558–561 (1993).
2. Kresse, G. & Furthmüller, J. Efficiency of ab-initio total energy calculations for metals and semiconductors using a plane-wave basis set. *Computational Materials Science* **6**, 15–50 (1996).
3. Kresse, G. & Furthmüller, J. Efficient iterative schemes for *ab initio* total-energy calculations using a plane-wave basis set. *Phys. Rev. B* **54**, 11169–11186 (1996).
4. Momma, K. & Izumi, F. *VESTA* : a three-dimensional visualization system for electronic and structural analysis. *J Appl Crystallogr* **41**, 653–658 (2008).
5. Korotcov, A. *et al.* Well-Aligned IrO 2 Nanocrystals. *Journal of Nanomaterials* **2007**, 1–17 (2007).
6. Perdew, J. P., Burke, K. & Ernzerhof, M. Generalized Gradient Approximation Made Simple. *Phys. Rev. Lett.* **77**, 3865–3868 (1996).
7. Hammer, B., Hansen, L. B. & Nørskov, J. K. Improved adsorption energetics within density-functional theory using revised Perdew-Burke-Ernzerhof functionals. *Phys. Rev. B* **59**, 7413–7421 (1999).
8. Grimme, S., Antony, J., Ehrlich, S. & Krieg, H. A consistent and accurate *ab initio* parametrization of density functional dispersion correction (DFT-D) for the 94 elements H-Pu. *The Journal of Chemical Physics* **132**, 154104 (2010).
9. Kresse, G. & Joubert, D. From ultrasoft pseudopotentials to the projector augmented-wave method. *Phys. Rev. B* **59**, 1758–1775 (1999).

10. Mathew, K., Sundararaman, R., Letchworth-Weaver, K., Arias, T. A. & Hennig, R. G. Implicit solvation model for density-functional study of nanocrystal surfaces and reaction pathways. *The Journal of Chemical Physics* **140**, 084106 (2014).

11. Mathew, K., Kolluru, V. S. C., Mula, S., Steinmann, S. N. & Hennig, R. G. Implicit self-consistent electrolyte model in plane-wave density-functional theory. *The Journal of Chemical Physics* **151**, 234101 (2019).

12. Islam, S. M. R., Khezeli, F., Ringe, S. & Plaisance, C. An implicit electrolyte model for plane wave density functional theory exhibiting nonlinear response and a nonlocal cavity definition. *The Journal of Chemical Physics* **159**, 234117 (2023).

13. Hossain, M. D., Huang, Y., Yu, T. H., Goddard III, W. A. & Luo, Z. Reaction mechanism and kinetics for CO2 reduction on nickel single atom catalysts from quantum mechanics. *Nat Commun* **11**, 2256 (2020).

14. Henkelman, G., Arnaldsson, A. & Jónsson, H. A fast and robust algorithm for Bader decomposition of charge density. *Computational Materials Science* **36**, 354–360 (2006).

15. Nørskov, J. K. *et al.* Origin of the Overpotential for Oxygen Reduction at a Fuel-Cell Cathode. *J. Phys. Chem. B* **108**, 17886–17892 (2004).

16. Hansen, H. A., Rossmeisl, J. & Nørskov, J. K. Surface Pourbaix diagrams and oxygen reduction activity of Pt, Ag and Ni(111) surfaces studied by DFT. *Phys. Chem. Chem. Phys.* **10**, 3722 (2008).

17. Eslamibidgoli, M. J., Huang, J., Kowalski, P. M., Eikerling, M. H. & Groß, A. Deprotonation and cation adsorption on the NiOOH/water interface: A grand-canonical first-principles investigation. *Electrochimica Acta* **398**, 139253 (2021).

18. Exner, K. S., Anton, J., Jacob, T. & Over, H. Chlorine Evolution Reaction on RuO2(110): Ab initio Atomistic Thermodynamics Study - Pourbaix Diagrams. *Electrochimica Acta* **120**, 460–466 (2014).

19. Rossmeisl, J., Logadottir, A. & Nørskov, J. K. Electrolysis of water on (oxidized) metal surfaces. *Chemical Physics* **319**, 178–184 (2005).

20. Rossmeisl, J., Qu, Z.-W., Zhu, H., Kroes, G.-J. & Nørskov, J. K. Electrolysis of water on oxide surfaces. *Journal of Electroanalytical Chemistry* **607**, 83–89 (2007).

21. Nong, H. N. *et al.* Key role of chemistry versus bias in electrocatalytic oxygen evolution. *Nature* **587**, 408–413 (2020).

22. Halck, N. B., Petrykin, V., Krtil, P. & Rossmeisl, J. Beyond the volcano limitations in electrocatalysis – oxygen evolution reaction. *Phys. Chem. Chem. Phys.* **16**, 13682–13688 (2014).

23. Fang, Y.-H. & Liu, Z.-P. Mechanism and Tafel Lines of Electro-Oxidation of Water to Oxygen on RuO_2 (110). *J. Am. Chem. Soc.* **132**, 18214–18222 (2010).

24. Ping, Y., Nielsen, R. J. & Goddard, W. A. The Reaction Mechanism with Free Energy Barriers at Constant Potentials for the Oxygen Evolution Reaction at the IrO_2 (110) Surface. *J. Am. Chem. Soc.* **139**, 149–155 (2017).

25. Busch, M., Ahlberg, E. & Panas, I. Electrocatalytic oxygen evolution from water on a Mn(iii–v) dimer model catalyst—A DFT perspective. *Phys. Chem. Chem. Phys.* **13**, 15069 (2011).

26. Busch, M. Water oxidation: From mechanisms to limitations. *Current Opinion in Electrochemistry* **9**, 278–284 (2018).

27. Binninger, T. & Doublet, M.-L. The Ir–OOOO–Ir transition state and the mechanism of the oxygen evolution reaction on IrO_2 (110). *Energy Environ. Sci.* **15**, 2519–2528 (2022).

28. López, M., Exner, K. S., Viñes, F. & Illas, F. Computational Pourbaix Diagrams for MXenes: A Key Ingredient toward Proper Theoretical Electrocatalytic Studies. *Advcd Theory and Sims* **6**, 2200217 (2023).

29. Exner, K. S. On the mechanistic complexity of oxygen evolution: potential-dependent switching of the mechanism at the volcano apex. *Mater. Horiz.* **10**, 2086–2095 (2023).

30. Exner, K. S. A Universal Descriptor for the Screening of Electrode Materials for Multiple-Electron Processes: Beyond the Thermodynamic Overpotential. *ACS Catal.* **10**, 12607–12617 (2020).

31. Razzaq, S. & Exner, K. S. Materials Screening by the Descriptor G_{max} (η): The Free-Energy Span Model in Electrocatalysis. *ACS Catal.* **13**, 1740–1758 (2023).

32. Nørskov, J. K. *et al.* Trends in the Exchange Current for Hydrogen Evolution. *J. Electrochem. Soc.* **152**, J23 (2005).

33. Kozuch, S. & Shaik, S. How to Conceptualize Catalytic Cycles? The Energetic Span Model. *Acc. Chem. Res.* **44**, 101–110 (2011).

34. Heenen, H. H., Gauthier, J. A., Kristoffersen, H. H., Ludwig, T. & Chan, K. Solvation at metal/water interfaces: An *ab initio* molecular dynamics benchmark of common computational approaches. *The Journal of Chemical Physics* **152**, 144703 (2020).

35. Exner, K. S. Importance of the Walden Inversion for the Activity Volcano Plot of Oxygen Evolution. *Advanced Science* **10**, 2305505 (2023).

36. Lewis, D. E. *Addition, Elimination and Substitution: Markovnikov, Hofmann, Zaitsev and Walden: Discovery and Development*. (Elsevier, Amsterdam, Netherlands, 2022).

37. Yu, S., Levell, Z., Jiang, Z., Zhao, X. & Liu, Y. What Is the Rate-Limiting Step of Oxygen Reduction Reaction on Fe–N–C Catalysts? *J. Am. Chem. Soc.* **145**, 25352–25356 (2023).

38. Hansen, H. A. *et al.* Electrochemical chlorine evolution at rutile oxide (110) surfaces. *Phys. Chem. Chem. Phys.* **12**, 283–290 (2010).

39. Sumaria, V., Krishnamurthy, D. & Viswanathan, V. Quantifying Confidence in DFT Predicted Surface Pourbaix Diagrams and Associated Reaction Pathways for Chlorine Evolution. *ACS Catal.* **8**, 9034–9042 (2018).

40. Gauthier, J. A., Dickens, C. F., Chen, L. D., Doyle, A. D. & Nørskov, J. K. Solvation Effects for Oxygen Evolution Reaction Catalysis on IrO_2 (110). *J. Phys. Chem. C* **121**, 11455–11463 (2017).

41. Briquet, L. G. V., Sarwar, M., Mugo, J., Jones, G. & Calle-Vallejo, F. A New Type of Scaling Relations to Assess the Accuracy of Computational Predictions of Catalytic Activities Applied to the Oxygen Evolution Reaction. *ChemCatChem* **9**, 1261–1268 (2017).

42. Ha, M.-A. & Larsen, R. E. Multiple Reaction Pathways for the Oxygen Evolution Reaction May Contribute to IrO_2 (110)'s High Activity. *J. Electrochem. Soc.* **168**, 024506 (2021).

43. Bhattacharyya, K., Poidevin, C. & Auer, A. A. Structure and Reactivity of IrO_x Nanoparticles for the Oxygen Evolution Reaction in Electrocatalysis: An Electronic Structure Theory Study. *J. Phys. Chem. C* **125**, 4379–4390 (2021).

44. González, D., Heras-Domingo, J., Sodupe, M., Rodríguez-Santiago, L. & Solans-Monfort, X. Importance of the oxyl character on the IrO2 surface dependent catalytic activity for the oxygen evolution reaction. *Journal of Catalysis* **396**, 192–201 (2021).

45. Mou, T., Bushiri, D. A., Esposito, D. V., Chen, J. G. & Liu, P. Rationalizing Acidic Oxygen Evolution Reaction over IrO_2 : Essential Role of Hydronium Cation. *Angew Chem Int Ed* **63**, e202409526 (2024).

46. Kwon, S. *et al.* Facet-Dependent Oxygen Evolution Reaction Activity of IrO_2 from Quantum Mechanics and Experiments. *J. Am. Chem. Soc.* **146**, 11719–11725 (2024).

47. Klyukin, K., Zagalskaya, A. & Alexandrov, V. Ab Initio Thermodynamics of Iridium Surface Oxidation and Oxygen Evolution Reaction. *J. Phys. Chem. C* **122**, 29350–29358 (2018).

48. Opalka, D., Scheurer, C. & Reuter, K. Ab Initio Thermodynamics Insight into the Structural Evolution of Working IrO_2 Catalysts in Proton-Exchange Membrane Electrolyzers. *ACS Catal.* **9**, 4944–4950 (2019).

49. Exner, K. S. & Over, H. Beyond the Rate-Determining Step in the Oxygen Evolution Reaction over a Single-Crystalline IrO_2 (110) Model Electrode: Kinetic Scaling Relations. *ACS Catal.* **9**, 6755–6765 (2019).

50. Geppert, J. *et al.* Microkinetic Analysis of the Oxygen Evolution Performance at Different Stages of Iridium Oxide Degradation. *J. Am. Chem. Soc.* **144**, 13205–13217 (2022).

51. Kuo, D.-Y. *et al.* Influence of Surface Adsorption on the Oxygen Evolution Reaction on IrO_2 (110). *J. Am. Chem. Soc.* **139**, 3473–3479 (2017).

52. Kuo, D.-Y. *et al.* Measurements of Oxygen Electroadsorption Energies and Oxygen Evolution Reaction on RuO_2 (110): A Discussion of the Sabatier Principle and Its Role in Electrocatalysis. *J. Am. Chem. Soc.* **140**, 17597–17605 (2018).

53. Bockris, J. O. & Reddy, A. K. N. *Volume 2 Modern Electrochemistry*. (Springer US, Boston, MA, 1973). doi:10.1007/978-1-4613-4560-2.

54. Dhaka, K. & Exner, K. S. Degree of span control to determine the impact of different mechanisms and limiting steps: Oxygen evolution reaction over Co3O4(001) as a case study. *Journal of Catalysis* **443**, 115970 (2025).
